# An integrative climate change vulnerability index for Arctic aviation and marine transportation

**DOI:** 10.1038/s41467-019-10347-1

**Published:** 2019-06-13

**Authors:** Nathan S. Debortoli, Dylan G. Clark, James D. Ford, Jesse S. Sayles, Emilia P. Diaconescu

**Affiliations:** 1 Department of Geography, McGill University, 805 Sherbrooke Street West, Montréal, QC H3A 2T5 Canada; 2 Priestley International Centre for Climate, University of Leeds, Leeds, LS2 9JT United Kingdom; 3 Ouranos Inc., Consortium Sur La Climatologie Régionale Et L’adaptation Aux Changements Climatiques, 550 Rue Sherbrooke O, Montréal, QC H3A 1B9 Canada

**Keywords:** Projection and prediction, Governance, Geography

## Abstract

Climate change vulnerability research methods are often divergent, drawing from siloed biophysical risk approaches or social-contextual frameworks, lacking methods for integrative approaches. This substantial gap has been noted by scientists, policymakers and communities, inhibiting decision-makers’ capacity to implement adaptation policies responsive to both physical risks and social sensitivities. Aiming to contribute to the growing literature on integrated vulnerability approaches, we conceptualize and translate new integrative theoretical insights of vulnerability research to a scalable quantitative method. Piloted through a climate change vulnerability index for aviation and marine sectors in the Canadian Arctic, this study demonstrates an avenue of applying vulnerability concepts to assess both biophysical and social components analyzing future changes with linked RCP climate projections. The iterative process we outline is transferable and adaptable across the circumpolar north, as well as other global regions and shows that transportation vulnerability varies across Inuit regions depending on modeled hazards and transportation infrastructures.

## Abstract (web summary)

It is important to understand how physical changes in Polar regions influence social systems and populations. Here the authors develop an Arctic Climate Change Vulnerability Index focusing on aviation and marine infrastructure in future climate scenarios and show that transportation system vulnerability varies across the region depending on modeled hazards and transportation infrastructure types.

## Introduction

As the IPCC SREX (Managing the Risks of Extreme Events and Disasters to Advance Climate Change Adaptation) and the Arctic Council’s Adaptation Actions for a Changing Arctic assert, improved tools for projecting and estimating future vulnerability are essential to protect and improve the health and wellbeing of communities around the world^1,2^. Herein, this paper develops a new approach for assessing and projecting vulnerability to climate change, applying the tool in the Canadian Arctic. The approach responds to two challenges that affect vulnerability research in general and indicator-based in particular^3^.

Firstly, the majority of studies on vulnerability are top down, focusing on climatic conditions best captured by models and identified by researchers as being important^4^. These may or may not be relevant, and while integrated assessments have sought to capture how socio-economic-demographic shape vulnerability, this traditional approach has been critiqued as poorly representing the real-world complexities of human-environment interactions^3–8^. Secondly, a significant body of case study research has developed over the last decade, focusing on complex interactions between climate change and society in specific locations, and have been described as bottom up as they focus on locally identified and relevant conditions^9^. These approaches provide rich detail but are often too context specific for informing decision making and lack a quantitative basis for incorporating climate projections to develop future vulnerability scenarios. This disconnect between top down and bottom up approaches, rooted in different disciplinary perspectives, presents a persistent stumbling block for developing credible future vulnerability scenarios^10^.

It is with these challenges in mind that this study proposes a new approach to developing vulnerability indices. Uniquely, the study starts with community-based research to develop a vulnerability index that incorporates both social and biophysical data with linked RCP projections across a region that is over 2 million km^2^, and is thus rooted in the climatic and socio-economic conditions that matter to a specific context. The approach is intended to complement and build on qualitative case studies, providing regional estimates of vulnerability that are increasingly demanded by decision makers^11^, along with specific community projections. Indeed, both the 2018 Expert Panel on Measuring Progress on Adaptation and Climate Resilience in Canada^12^ and the 2018 Auditor General’s reports on adaptation in Canada, highlighted the importance of delineating which regions, sectors, and government services are most vulnerable^13^.

In this study, we focus on Inuit Nunangat (Inuit homeland) of the Canadian Arctic. Encompassing over 50% of Canada’s coastline and 35% of the country’s landmass, Inuit Nunangat is home to 54,000 people living in 50 communities that range in size from 103 to 7740^14^ people. The region experiences some of the most pronounced climate change globally. Further, the regions Inuit population are generally are more sensitive to climate change given socio-economic conditions and stresses^15^, as well as a reliance on the environment for livelihoods and well-being. High health burdens, including food insecurity, unintentional injury, and mental health, are linked to environmental systems and the region’s colonial history (Supplementary Note 1).

Though environmental changes are anticipated to have negative short-term impacts on health, housing, the economy, and transportation systems, communities are also recognized for their resilience—a function of Indigenous knowledge systems, diversified livelihoods, and systems of self-governance^16,17^. Indeed, communities across the Canadian Arctic have a history of adapting to environmental change, developing or shifting harvesting activities and patterns of travel and, more recently, transitioning economic systems^9,18^. In addition, over the past decade, communities and regional governments have been taking proactive steps to adapt and find synergies, although most adaptation efforts to-date are either in the planning stage or do not involve concrete actions to reduce vulnerability^1,2,17,19–23^ (Supplementary Notes 2, 3).

Focusing on transportation system characteristics and infrastructure in communities, this study finds the most substantial driver of present and future vulnerability varies widely between communities and regions across Inuit Nunangat. At present, the most vulnerable aviation systems are observed in Nunatsiavut and the most vulnerable marine systems are observed in Nunavut; this reflects both social and biophysical patterns across Inuit Nunangat. Furthermore, vulnerability for the entire Inuit Nunangat is projected to increase over the coming decades, increasing on average across regions by 58% for future scenarios compared to the baseline. Under the 4.5 and 8.5 scenarios, mean modeled vulnerability increased 56% and 60%, respectively, compared with present vulnerability.

## Results

### Exposure

**Fig. 1:**
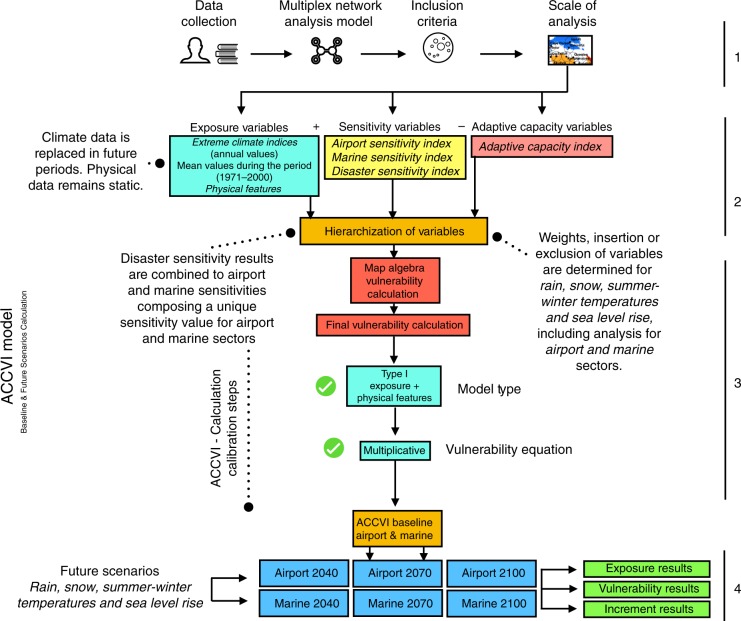
The diagram illustrates the four phases to develop the ACCVI framework. Phase 1 includes data collection and semi-structured interviews with decision makers, a systematic literature review translated into a multiplex network analysis model, inclusion criteria, and the use of buffer areas of 100-km to assess communities at local and regional scales. Phase 2 calculates exposure, sensitivity and adaptive capacity indices while hierarchizing variables. Phase 3 involves the final index calculation by applying map algebra techniques to combine indices. This phase was calibrated with two model types and two vulnerability equations. In the figure and throughout the article are only shown best calibration results symbolized by model Type I model and the Multiplicative Equation. Calculation results includes baseline and future IPCC RCP 4.5 and 8.5 scenarios for aviation and marine sectors, dissected into four climate components (rain, snow, summer-winter temperatures) and sea level rise projections. In Phase 4, three types of results were produced: (**a**) exposure, where climate and biophysical variables are calculated; (**b**) vulnerability, where exposure results are combined using the vulnerability equation which incorporate socioeconomic dimensions of sensitivity and adaptive capacity for both aviation and marine sectors; (**c**) increment change, where final vulnerability index results are represented as fractional changes

**Fig. 2:**
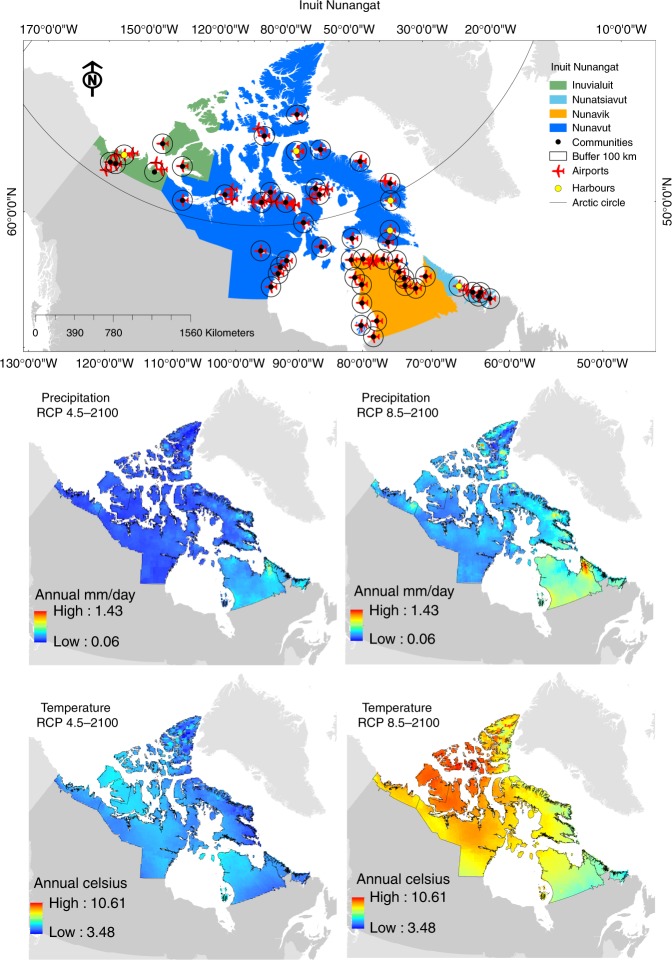
The top map shows the Inuit Nunangat region—which includes four divisions (Inuvialuit, Nunavut, Nunavik and Nunatsiavut)—and 50 inhabited communities (black dots). Buffer areas of 100-km represent areas analyzed in each community; red airplanes are airports and yellow dots harbors. The four bottom maps illustrate climate models increase values (delta) for annual mean precipitation (mm/day) and annual mean temperature (°C) for both RCP4.5 and 8.5 scenarios at the end of the century (2100)

Exposure results are calculated by combining climate model variables such as rain, snow and temperatures averages and extreme indices; and physical features, such as slope, elevation, soils, permafrost, wind, and water distance (Supplementary Notes 4–10; Supplementary Tables 1–7; Supplementary Fig. 1). Based on the Arctic Climate Change Vulnerability Index (ACCVI model–Fig. 1), projected rain, snow, winter-summer temperature and sea level rise showed substantial increase in exposure across Inuit Nunangat through 2100. Annual mean temperature was modeled to increase between 6.4 °C (RCP4.5) and 10.6 °C (RCP8.5) on average across the region, while annual mean precipitation is projected to increase by 0.80 mm/day (RCP 4.5) and 1.43 mm/day (RCP8.5) on average by 2100^24^ (Fig. 2).

**Fig. 3:**
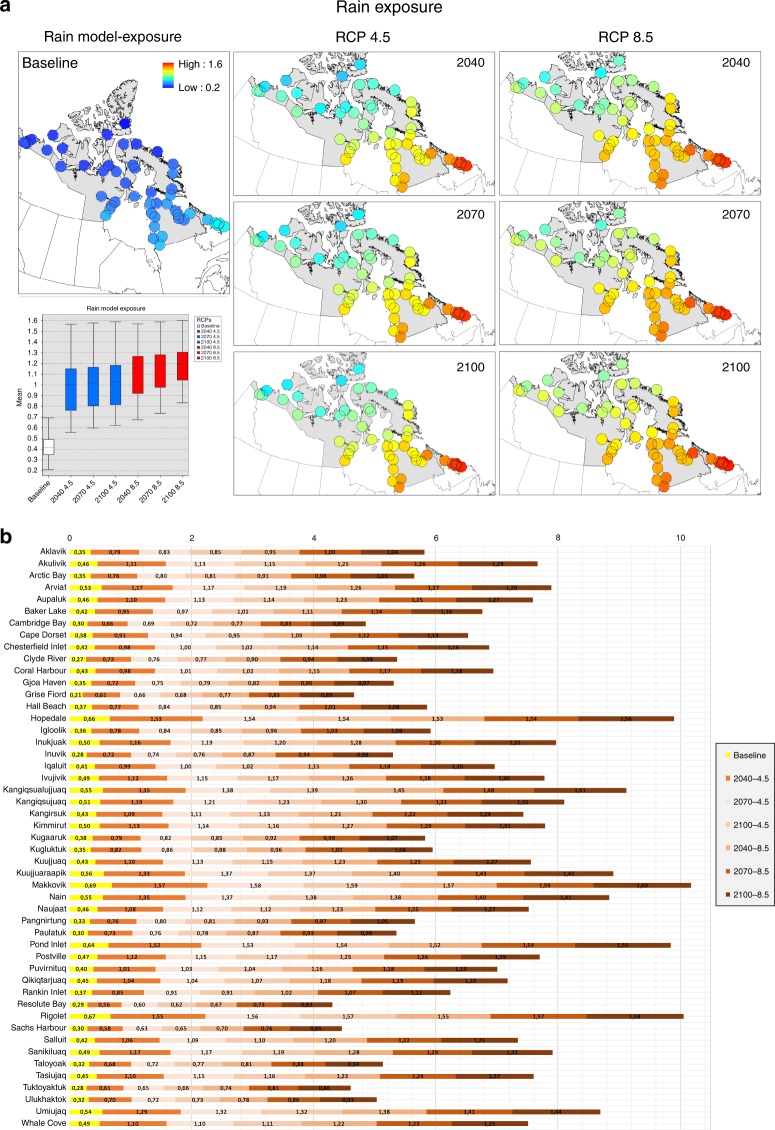
Exposure maps. **a** Rain exposure maps. Rain exposure model for 49 Inuit Nunangat communities including baseline and future RCP 4.5–8.5 scenarios (2040, 2070, 2100). Boxplot includes baseline and RCP 4.5 (blue) and RCP 8.5 (red) scenarios mean values, max-min values and outliers (when applicable). The boxplot *x*-axis represents the baseline and the subsequent scenarios and the *y*-axis the mean exposure values. The community of Quaqtaq was not included in the rain and snow models due to unavailability of physical features for its entire buffer. **b** Communities rain exposure graph. Accumulative rain model exposure for 49 communities including baseline and future scenarios for the multiplicative equation. Yellow bars represent baseline period and orange gradient bars delimit RCP 4.5 and 8.5 future scenarios. The community of Quaqtaq was not included in the rain and snow models due to unavailability of physical features for its entire buffer

Among the assessed hazards, current exposure for summer-winter temperatures and rain have the widest distribution of exposure values (close to 1) when compared to other hazards (see Table 1 for all models). Future exposure scenarios indicate that summer-winter temperatures and rain models surpass the 0–1 index window (indicating an extreme future climate). In general, the models agree that exposure will increase towards the end of the century for rain, temperatures and sea level rise across the Arctic Archipelago and North Atlantic. While both winter-summer temperatures will increase most in more southerly communities, rain and snow exposure is projected to increase the most in eastern Inuit Nunangat. Nunavik and Nunatsiavut are projected to be most exposed under the rain model (see rain model example Fig. 3a, b and other hazards in Supplementary Fig. 2 and Supplementary Data 1). The three communities that were most commonly high ranked for exposure are Makkovik, Rigolet, and Hopedale.

**Table 1:** Highest projected exposure results for all models, scenarios and top 3 regions and communities

Exposure model	Future exposure scenarios		Exposure (excluding outliers)	Future exposure scenarios (excluding outliers)		Regions exposure	Communities exposure
	Top RCP 4.5	Top RCP 8.5	Mean baseline	Top mean exposure RCP 4.5	Top mean exposure RCP 8.5	Ranked top 3	Ranked top 3
Rain	2100	2100	0.43	1.04	1.19	(1) Nunatsiavut (2) Nunavik (3) Nunavut	(1) Makkovik (2) Rigolet (3) Hopedale
Snow	2040	2040	0.27	0.59	0.59	(1) Nunatsiavut (2) Nunavik (3) Nunavut	(1) Puvirnituq (2) Naujaat (3) Grise Fiord
Temperature winter	2100	2100	0.48	1.21	1.32	(1) Nunatsiavut (2) Nunavik (3) Inuvialuit	(1) Makkovik (2) Rigolet (3) Hopedale
Temperature summer	2100	2100	0.56	1.32	1.40	(1) Nunatsiavut (2) Nunavik (3) Inuvialuit	(1) Makkovik (2) Hopedale (3) Kuujjuaraapik
Sea level rise airport	2040	2040	0.42	0.64	0.66	(1) Nunatsiavut (2) Inuvialuit (3) Nunavut	(1) Makkovik (2) Tuktoyaktuk (3) Hopedale
Sea level rise marine	2040	2040	0.13	0.81	0.82	(1) Nunatsiavut (2) Nunavik (3) Nunavut	(1) Pangnirtung (2) Qikiqtarjuaq (3) Tuktoyaktuk

Results are expressed using mean values. Snow, winter and summer temperatures and sea level rise exposure maps, boxplots, and calculated values are available in Supplementary Fig. 2 and Supplementary Data 1

Sea level rise models indicate that Nunatsiavut and Inuvialuit regions are especially sensitive to climate change. Under the sea level rise model across Inuit Nunangat, a higher magnitude of change is projected under the 8.5 scenario. Contrary to this trend, exposure decreases at the end of the century, notably in the Hudson Bay area and Canadian Archipelago, driven by isostatic rebound. This decrease in exposure could generate new challenges for ports, such as increased docking distance and potential local marine and coastal ecosystem changes.

### Vulnerability

**Fig. 4:**
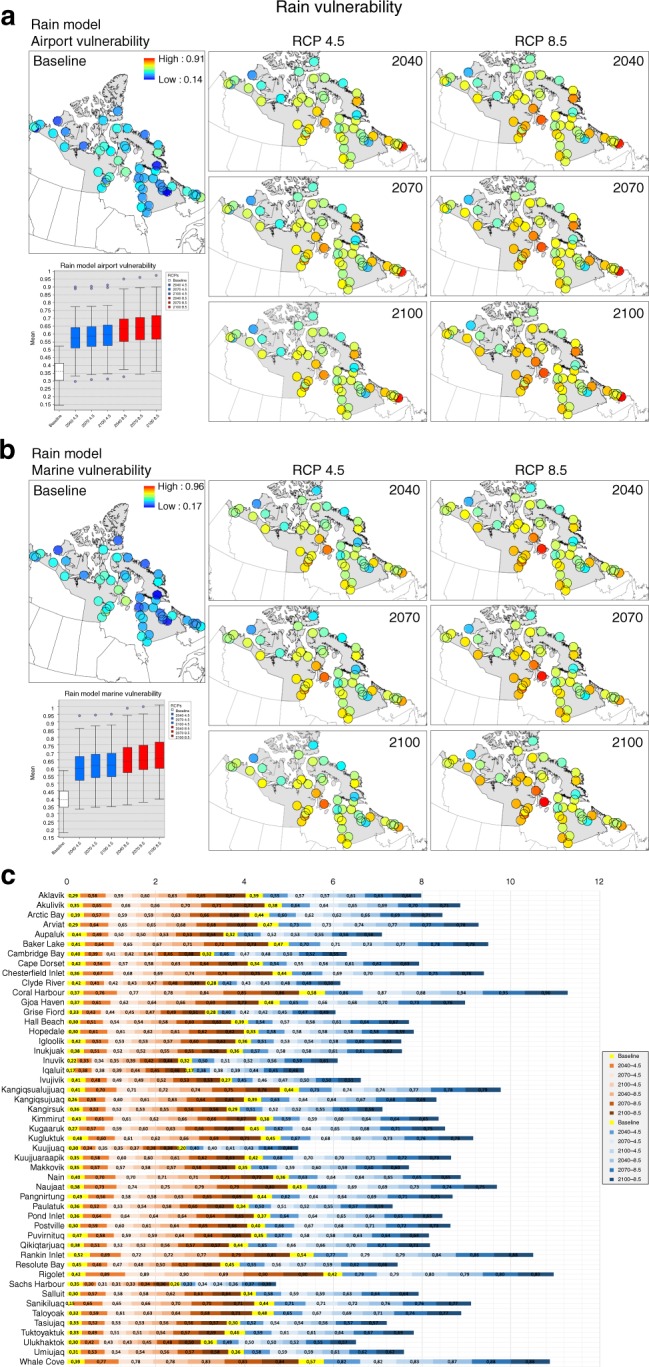
Rain vulnerability maps. **a**, **b** Rain model for airport and marine multiplicative vulnerability equations considering 49 Inuit Nunangat communities. Includes baseline and future RCP 4.5–8.5 scenarios (2040, 2070, 2100). Boxplots includes mean, max-min, and outlier values. The *x*-axis represents the baseline and the subsequent scenarios and y-axis mean vulnerability. The community of Quaqtaq was not included in the rain and snow models due to unavailability of physical features for its entire buffer. **c** Communities vulnerability graph. Accumulative rain model vulnerability including baseline and future scenarios for the multiplicative equation for 49 Inuit Nunangat communities. Yellow bars represent baseline for both airport and marine models, red bars gradient delimit airport vulnerability and blue bars gradient marine vulnerability. The community of Quaqtaq was not included in the rain and snow models due to unavailability of physical features for its entire buffer

Vulnerability results reflect coupled exposure, sensitivity, and adaptive capacity values (rain model example Fig. 4a, b and other hazards in Supplementary Fig. 3). The results illustrate the importance of socioeconomic and political factors in determining communities’ adaptive capacity and/or sensitivities related to climate change exposure and local physical features (Table 2). When considering sensitivity to airport infrastructure, the Nunatsiavut region has the highest modeled values, while marine values are higher for Inuvialuit region. Disaster sensitivity was estimated to be most pronounced in Nunavut, though the region has the highest modeled adaptive capacity of Inuit Nunangat (see Supplementary Data 2).

**Table 2:** Interpretation of ACCVI results

Model output type	Application	Policy implication
Gross value (vulnerability)	Vulnerability results compare vulnerability gross values among regions to identify the greatest impacts in a future period	There is a high probability that regions with the highest vulnerability values in the future periods require adaptations. Additionally, the communities with the highest vulnerability gross value in the future are likely presently the most vulnerable too
Incremental result (increment)	Increment results identifies locations where future vulnerability increases are the greatest compared to the baseline	Even if the net impact in future scenarios is not high compared to the baseline, a minimum increment change may bring unseen changes to the system—depending on past conditions—increasing adaptation challenges/efforts. Communities that have never experienced a hazard can be more vulnerable to a shift in exposure

Guidelines to use Gross values and Incremental results application and for policy implications

Projected changes for the four vulnerability models are affected substantially by sensitivity and adaptive capacity layers, moderating climate impacts in some regions (Table 3 and Supplementary Data 3). The spatial characteristics of vulnerability throughout Inuit Nunangat are also more distributed in comparison to exposure patterns.

**Table 3:** Highest projected vulnerability results for all models, scenarios and top 3 regions and communities

Vulnerability model	Vulnerability future scenarios		Vulnerability (excluding outliers)	Vulnerability future scenarios (excluding outliers)		Regions vulnerability (airport + marine)	Communities vulnerability (airport + marine)	Top vulnerability drivers at baseline
	Top RCP 4.5	Top RCP 8.5	Mean baseline	Top mean RCP 4.5	Top mean RCP 8.5	Ranked top 3	Ranked top 3	
Rain airport	2100	2100	0.36	0.58	0.63	(1) Nunatsiavut (2) Nunavut (3) Nunavik	(1) Coral Harbor (2) Rigolet (3) Whale Cove	(1) Marine sensitivity (2) Airport sensitivity (3) Disaster sensitivity
Rain marine	2100	2100	0.39	0.61	0.67			
Snow airport	Equal	2040	0.30	0.41	0.41	(1) Nunatsiavut (2) Nunavut (3) Nunavik/ Inuvialuit	(1) Naujaat (2) Rankin Inlet (3) Coral Harbor	(1) Marine sensitivity (2) Marine sensitivity (3) Marine sensitivity
Snow marine	2040	2040/ 2070	0.33	0.45	0.44			
Temperature DJF airport	2100	2100	0.37	0.64	0.69	(1) Nunatsiavut (2) Nunavik (3) Nunavut/ Inuvialuit	(1) Rigolet (2) Coral Harbor (3) Whale Cove	(1) Winter exposure (2) Marine sensitivity (3) Disaster sensitivity
Temperature DJF marine	2100	2100	0.40	0.67	0.72			
Temperature JJA airport	2100	2100	0.41	0.69	0.72	(1) Inuvialuit (2) Nunavut (3) Nunavik	(1) Whale Cove (2) Rigolet (3) Baker Lake	(1) Disaster sensitivity (2) Summer exposure (3) Marine sensitivity
Temperature JJA marine	2100	2100	0.44	0.71	0.75			
Sea level rise airport	2040	2040	0.35	0.43	0.44	(1) Inuvialuit (2) Nunavut (3) Nunatsiavut	(1) Tuktoyaktuk (2) Kugluktuk (3) Pangnirtung	(1) Marine sensitivity (2) Marine sensitivity (3) Disaster sensitivity
Sea level rise marine	2040	2040	0.28	0.53	0.53			

Results are expressed using mean values. Snow, summer and winter temperatures, and sea level rise vulnerability maps, boxplots and calculated values are available in Supplementary Fig. 3 and Supplementary Data 3

When considering the four Inuit Nunangat regions, Nunatsiavut and Nunavut are estimated to have the highest levels of vulnerability to projected climate impacts based on the models. Communities such as Rigolet, Coral Harbor and Whale Cove are estimated to be among the most vulnerable communities according to our calculations (Fig. 4c). This reflects the cumulative indices score taking into account exposure, sensitivity and adaptive capacity indicators for both airport and marine infrastructures (see Supplementary Data 2).

### Percentage increment

**Fig. 5:**
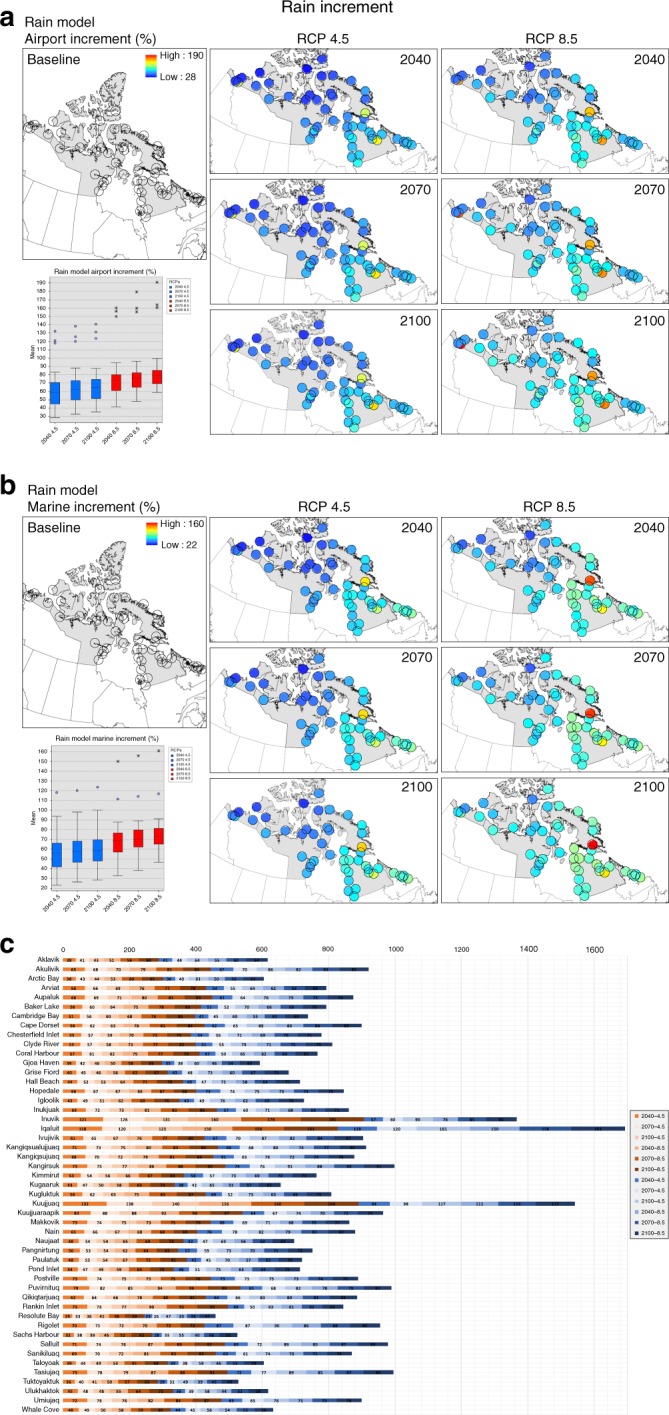
Rain increment maps. **a**, **b** Increment percentage/anomalies for the rain model (airport and marine multiplicative vulnerability equations) including 49 Inuit Nunangat communities and future RCP 4.5 (blue) and 8.5 (red) scenarios (2040, 2070, 2100). Boxplots include mean values, max-min values and outliers. The *x*-axis represents the scenarios and the *y*-axis the mean increment/anomalies values in percentage. The blank baseline map means that there are no baseline values for the increment analysis. The community of Quaqtaq was not included in the rain and snow models due to unavailability of physical features for its entire buffer. **c** Communities rain increment. Increment percentage for the rain model including future scenarios for the multiplicative equation considering 49 Inuit Nunangat communities. Red bars gradient delimits airport increment/anomalies and blue bars gradient marine increment/anomaly. The community of Quaqtaq was not included in the rain and snow models due to unavailability of physical features for its entire buffer

The percentage increment/anomalies analysis captures fractional changes more clearly within vulnerability calculations (see methods for details), identifying regions and communities with the highest percent of change in future vulnerability (Fig. 5a, b and Supplementary Fig. 4). Sea level rise for the marine sector is projected to have the highest percent change (100%), followed by rain and winter temperatures (Table 4 and Supplementary Data 4). Increments are estimated to be higher for Nunatsiavut (70%) and Nunavik regions (62%) particularly in major centers throughout Inuit Nunangat (Fig. 5c) such as: Iqaluit (high exposure), Kuujjuaq (presently low modeled adaptive capacity), and Inuvik (presently high modeled marine sensitivity). This indicates that current socioeconomic conditions and infrastructure constrain the ability to cope with future climatic exposures.

**Table 4:** Increment percentage/anomalies results for all models, scenarios and for the top 3 regions and communities

Model	Scenarios increment		Vulnerability increment		Regions vulnerability Increment (airport + marine)	Communities vulnerability Increment (airport + marine)
	Top RCP 4.5	Top RCP 8.5	Top mean RCP4.5	Top mean RCP8.5	Ranked top 3	Ranked top 3
Rain airport	2100	2100	66%	83%	(1) Nunatsiavut (2) Nunavik (3) Nunavut	(1) Iqaluit (2) Kuujjuaq (3) Inuvik
Rain marine	2100	2100	75%	75%		
Snow airport	2040	2040	41%	41%	(1) Nunavik (2) Nunatsiavut (3) Nunavut/ Inuvialuit	(1) Iqaluit (2) Kuujjuaq (3) Inuvik
Snow marine	2040/2070	2040	26%	26%		
Temperature winter airport	2100	2100	73%	85%	(1) Nunavik (2) Nunatsiavut (3) Nunavut/ Inuvialuit	(1) Iqaluit (2) Inuvik (3) Kuujjuaq
Temperature winter marine	2100	2100	67%	78%		
Temperature summer airport	2100	2100	70%	78%	(1) Nunatsiavut (2) Nunavik (3) Nunavut	(1) Iqaluit (2) Kuujjuaq (3) Inuvik
Temperature summer marine	2100	2100	65%	73%		
Sea level rise airport	2040	2040	23%	26%	(1) Nunatsiavut (2) Nunavik (3) Nunavut	(1) Iqaluit (2) Rigolet (3) Kuujjuaq
Sea level rise marine	2040	2040	104%	106%		

Results are expressed using mean values. Snow, winter and summer temperatures, and sea level rise increment maps, boxplots, and calculated values are available in Supplementary Fig. 4 and Supplementary Data 4

## Discussion

Air and marine transportation systems play invaluable roles in the health, wellbeing, and economic vitality of the Canadian Arctic^25–28^. These transportation systems are vulnerable to climate change due to persistent infrastructure inequities between the Canadian North and southern Canada^9^, constrained emergency response capacities^29^, and increasing biophysical changes driven by climate change. Potential impacts are concerning and underpin the need for continued and enhanced investment in adaptation.

Though developed in a Canadian Arctic context, ACCVI’s approach is highly adaptable and has broad relevance across circumpolar regions and other regions globally, being limited largely by environmental and socioeconomic data availability and projections. Using a new methodological approach to model vulnerability, we project the effects of various climate change scenarios on air and marine transportation systems across the Canadian Arctic. Based on the four hazards assessed we find that system vulnerability varies widely across the region and is projected to increase for at least the next 20 to 70 years. We also outline an iterative and transferable approach to model future climate change risks and delineate adaptation needs.

Developing complex and integrated vulnerability assessments comes with challenges and limitations. The authors acknowledge that several metrics available in the literature are absent of this analysis due to data availability or an inability to incorporate data into spatial assessments of aviation and marine sectors (Supplementary Discussion). Further, it was beyond the scope of this study to project potential socioeconomic pathways or changes to physical infrastructure. These limitations are highlighted as persistent scientific gaps in linked climate change socioeconomic and environmental modeling^25,30,31^. Additional work is needed to address these gaps, with potential for continued advances in the vulnerability science and adaptation planning. In this context, community scale results are to be understood as relative and reflective only of variables and systems evaluated.

Finally, it is important to note that vulnerability indexes are approximate methods of explaining how much a given system can be impacted by an external threat. In climate change science, the cause and effect between threats (exposure vector) and the end product (vulnerability) is a non-linearity. This non-linear relationship is produced by a condition that leads the system to a destabilizing threshold point which ultimately affects the entire system interrelationships. Such relationships between vulnerability drivers are so complex that is practically impossible to formulate indices that can unequivocally measure the vulnerability of a system (with progressive difficulty towards large areas)^32–34^. There are also indirect hazards, such as an oil spill’s impact on local food security, that were not specifically assessed or captured in the model. These indirect vulnerabilities are highly important, and while select variables, such as emergency response capacity and adaptive capacity, have wide implications, additional research is needed to more fully incorporate indirect scenarios accurately into models.

Projecting the vulnerability of social systems to biophysical changes is a necessary foundation to adaptation policies and building resilience across the Canadian Arctic, and more broadly in vulnerable regions around the world^35,36^. This study is the first of its kind to assess Arctic climate change system vulnerability at such vast scale (over 2 million km^2^ land area) and resolution (∼25-km^2^) while promoting anticipatory adaptation through the identified vulnerabilities in air and marine transportation sectors. Based on our findings, we have outlined key areas needing adaptation.

First, under present conditions, air transportation systems are inhibiting timely access to definitive medical care, as well as reliable and regular access to many communities. To reduce sensitivity, weather observations and forecasts need to be reliably and regularly available, runways length needs to be appropriate for community needs and growing transport demand, and instrument approaches need to be improved and more widely available given that weather conditions and surrounding terrain often limit the ability of aircraft to safely land in communities across Inuit Nunangat^13^.

Second, improvements in the coverage of detailed marine charting and navigational information is needed in vulnerable and heavy traffic regions to improve safety of shipping. Investments are specifically needed in the Arctic Archipelago, Hudson Bay, and Inuvialuit regions where vulnerability is projected to increase the most. Additionally, small ports have potential to reduce risks, especially in Inuvialuit^29^. Development of new ports should account for sea level decreases or rise.

Third, there is a need for large investments in infrastructure across the region in order to meet formal Canadian Federal Agency Standards^19^ as well to provide equity between south and north amenities and operational safety.

Fourth, adaptive capacity is influenced by a variety of social, political, economic, technological, and institutional factors^37^. Based on the adaptive capacity model (Supplementary Note 11 and Supplementary Data 2), we noted the majority of estimated highly adaptive communities are located in Nunatsiavut and Nunavik. Communities in Nunavik had high scores due to generally higher high school graduation and trade certificate attainment, as well as high presence of Inuktitut in households. For communities in Nunatsiavut, attainment of high school and trades certificates in the five communities increased relative scores. The lowest levels of adaptive capacity, as captured in the model, are distributed across the Central Arctic and Kivalliq region. This was due to lower levels of high school and trade certificate attainment and lower CWI scores.

There are multiple opportunities for capacity building and adaptation across the Canadian Arctic^9,16,38^. As highlighted by low estimated adaptation capacity in many communities, policies are needed to improve education, health, and income inequity, with many studies demonstrating the importance of Indigenous knowledge in building resilience^16^. Initiatives that address these social challenges will also likely improve a community’s ability to plan for and respond to climate change or disasters.

In such cases, first, it is essential that vulnerability assessments and adaptation plans look at various spatial scales to target at-risk populations. We highlight wide variation in exposure, sensitivity, and adaptive capacity across Inuit Nunangat. While we did not assess intra-community variation, there is likely high variation between households. Adaptation planning should reflect various scales of needs and vulnerability. Second, emergency managers and public health officials should prepare for increasing probabilities of small and large disasters due to climate change, shifts in marine traffic, and changing demographics. Emergency and disaster response across the Canadian Arctic is limited by a lack of interagency training, a lack of community-based resources, knowledge, preparedness, and minimal resources beyond DND capacities^29^. Capacity could be built through additional multi-agency training for both small (multi-day power plant failure) and large (airplane crash) incidents. Improvements in airport infrastructure where runways are too short for CC-130 or C17 to land and where instrument minimums frequently limit aeromedical access is essential to improving response.

Additional methodological developments and the application of coupled social and biophysical vulnerability modeling is necessary to further our understanding of where, when, and how communities and populations are vulnerable to climate change, both in the Canadian Arctic and globally. Uncertainties related to the static geospatial datasets currently available (e.g., poor understanding of the distribution of ground ice and permafrost) and identifying other data (e.g., historical storm surge, flooding, erosion, permafrost, and landslide risk-classification products and/or future projections made by process-based models) would be useful for developing other vulnerability models and assessments such as this.

It is essential that research to model vulnerability meaningfully incorporates local knowledge and/or Indigenous Knowledge and community perspectives^10^. Further, it is important that research teams are multidisciplinary and responsive to community experiences. Our approach was bolstered by using iterative steps to understand social and biophysical interactions, particularly a multiplex network analysis (Supplementary Note 12; Supplementary Tables 8–10; Supplementary Fig. 5). Areas for future growth include linking dynamic social scenarios or pathways and analyzing additional livelihood sectors.

Research has demonstrated that anticipatory adaptations are often more cost effective than reactive adaptation. It is essential that decision makers can anticipate future risks, can visualize where risks are highest, and understand how social and biophysical factors are interacting to create risk. The Arctic Climate Change Vulnerability Index approach developed in the paper helps to address these knowledge gaps for the transportation sector across Inuit Nunangat and outlines a method for future studies in other fields of vulnerability research.

## Methods

### Background

Across much of the Canadian Arctic, infrastructure and transportation systems are not sufficiently meeting basic needs^22,39–42^. Transportation systems and community infrastructure across Inuit Nunangat are keystones to human security^43^. Health care delivery, food security, potable water access, electricity, communications, and economic activities often rely on a community’s single diesel generator, one gravel runway, several petrol/diesel storage tanks, and a few satellite dishes and cell towers.

### Modeling framework

Focusing specifically on aviation and marine transportation vulnerability to climate change – due the availability of data and high importance of these sectors to communities – we develop a four-step process to assess current and future vulnerability in Inuit Nunangat (Fig. 1). We term this model the Arctic Climate Change Vulnerability Index (ACCVI). Results are presented at a community scale since the vulnerability lens applied in this work focuses on impacts to social and economic systems.

Results from the ACCVI model include both projected gross relative changes (gross value) in vulnerability from baseline to future scenario, as well as the relative increase in vulnerability (increment results). Both model outputs are insightful and contribute to a more holistic understanding of projected vulnerability change (Table 2). Since results are relative values, they must be interpreted by compared values among communities and regions or over time under a given model.

For vulnerability analysis with the ACCVI, sensitivity and adaptive capacity indices are held constant for future projections due to data and knowledge constraints; this is consistent with other studies^44,45^ and represents a key methodological challenge for developing future vulnerability indicators. Therefore, vulnerability results should be interpreted as how Inuit Nunangat airport and marine infrastructure would be affected if projected climate change were part of today’s social conditions. Hence, future changes at the sensitivity and adaptive capacity indicators would engender different vulnerability outcomes. Qualitative data were used to assist with interpretation of model outputs and development of policy relevant recommendations (Supplementary Notes 13–18).

### Air transportation

Unlike municipalities in southern Canada, interruptions to air transit for a few days can severely reduce food availability in most communities^46^. With the exception of Inuvik and Tuktoyaktuk, every community across Inuit Nunangat depends solely on air transportation to bring in perishable goods, food, mail, on a year around basis^47^. Aviation is also depended on for the majority of intercommunity travel across the region, including movement of technicians, mine employees, municipal and territory employees, and health care providers. Further, aviation infrastructure is a key component of the Canadian Department of Defense’s (DND) regional readiness and is relied upon for communication and emergency rerouting options of over 10,000 annual trans-polar flights^48^.

In most communities, an interruption to air travel also has potential to obstruct any means of patients accessing definitive healthcare during medical emergencies^29^. The Office of the Auditor General reported that one prominent aeromedical evacuation company in the region had to cancel 29% of emergency medical evacuations annually due to a lack of reliable weather reporting^43^. This is of particular concern, given most health centers across Inuit Nunangat do not have full-time physicians and lack resources to provide advanced life support to more than one patient; life-saving measures for most emergencies requires timely aeromedical access.

### Marine transportation

Marine transportation plays a critical role in the movement of goods to communities across the Canadian Arctic. For the majority of Inuit Nunangat communities, sealifts are the most cost-effective means of delivering non-perishable food, jet fuel, diesel for power production, gasoline, vehicles and construction equipment, and building supplies. Although the majority of communities rely on small docks and boat ramps, there are ongoing discussions of deep-sea ports in Pond Inlet and Iqaluit. A small craft harbor also exists in Pangnirtung. Though conventional shipping infrastructure is limited across much of the Canadian Arctic, navigational telex (provision of navigational and meteorological warnings and forecasts) are available along some of Baffin Island and the Hudson Bay. Additionally, electronic navigational charts are available around most communities. Canadian Coast Guard presence and icebreaking, oceanographic hazard mapping, satellite-based navigation systems, and Arctic shipping regulations are also key resources and systems that improve shipping safety across the region.

Marine access has historically been restricted by ice conditions to a window of one to three-month between July and September; however, shipping windows have become increasingly dynamic due to longer open-water season and the expanding presence of icebergs in some areas^49,50^. Changes are also leading to increases in Arctic marine traffic regionally and through the Northwest Passage. Between 1990 and 2015, distance traveled by vessels in the Arctic increased by over 150%^51^.

### Vulnerability approach

We use a vulnerability approach to assess both physical and social factors that influence exposure, sensitivity, and adaptive capacity to climate change impacts^6,35,36,52,53^, formally expressing vulnerability as a function of^35,54^:

1 $$V_{{{ist}}} = {\int} {(E_{{{ist}}} + S_{{{ist}}} - {\mathrm{AC}}_{{{ist}}})}$$

where vulnerability (*V*) is a function of exposure (*E*), sensitivity (*S*), and adaptive capacity (AC), given a community *i*, to stimulus *s*, in time *t*.

### Vulnerability indices

Over the past two decades, methods and approaches have been refined to better capture and analyze long-term climate change vulnerability through use of targeted variables which rely on accurate and well parametrized models^5,55^. Common methods for quantifying vulnerability currently include use of score cards, quantitative indices, and targeted models or tools^56,57^. Vulnerability indices require appropriate indicators to integrate social and environmental regional data that reflects spatial and temporal patterns of vulnerability^58^. Indices demand variables that are easily applicable, measurable, accessible, transferable, and non-redundant and depending on the vulnerability being assessed, they should integrate both quantitative and qualitative criteria while defining a system needs and boundaries^59–61^.

**Fig. 6:**
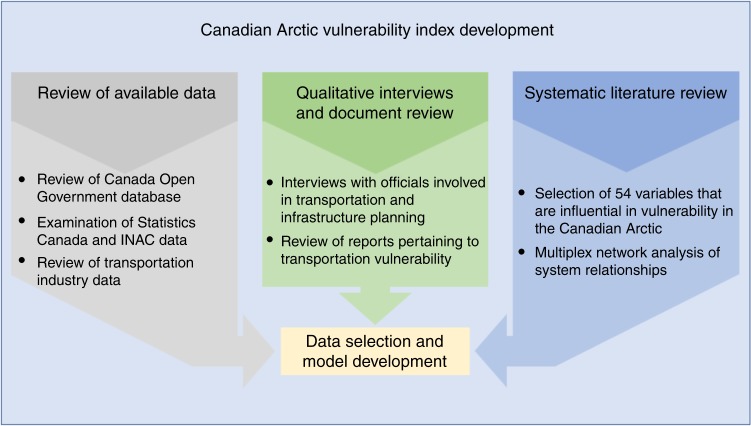
Development of the Arctic Climate Change Vulnerability Index for Northern Aviation, Shipping, and Infrastructure followed an iterative and parallel process. The method also emphasized incorporating regional and local knowledge from the large region through a systematic literature review and select key informant interviews

In the Canadian Arctic, it is essential that an index, or any other means of quantifying vulnerability, also reflects Inuit values and is sensitive to the way in which communities are labeled and represented^62^. As widely described throughout the literature, assessment of climate change vulnerability in the North American Arctic must be informed by, and rooted in, the regional history^16,63^. This includes accounting for rapid socioeconomic and cultural changes that have occurred over the last half-century, as well as trauma associated with economic and social policies, oppression, and colonization^9,64,65^ (Supplementary Note 1). Therefore, this study is informed by the substantial body of Arctic human dimensions of climate change scholarship, much of which builds upon community voices and observations (Fig. 6).

Variable selection for the ACCVI model is based in two main steps. Step 1, key informant interviews (*n* = 24) across the region and systematic literature review (*n* = 155 documents) of vulnerability case-studies to iteratively develop a model of livelihood and transportation systems in the Canadian Arctic. Step 2, development of a multiplex network analysis model^66^ to assess variables relationships in the exposure, sensitivity, and adaptive capacity dimensions (Supplementary Notes 12–18; Supplementary Tables 8–10; Supplementary Fig. 5).

### Merging data

Based on the multiplex network analysis results of Freeman’s degree centrality C_D_ and variables ranking, interviews, and the document review, we identified key variables and characteristics that are likely influential or highly correlated with transportation vulnerability across the region and would ideally be tracked. Qualitative data sources were used to triangulate exposures, sensitivities, and adaptive capacity components that were most important (Supplementary Notes 13–16; Supplementary Tables 8 and 9). Available data that could represent key areas were then filtered using the following inclusion criteria. First, Data that might change over time needed to be regularly updated (at least every 5 years) so that indices could be continually updated; Second, exposure data needed to be linked to RCP scenarios 4.5 and 8.5 and be available for 2040, 2070, and 2100 with a resolution of ∼25-km or smaller; Third, sensitivity and adaptive capacity data needed to be available for all communities and be comparable across Inuit Nunangat regions. Based on this process, we selected 8 exposure variables, 18 sensitivity variables, and 10 variables to quantify adaptive capacity (Supplementary Table 10).

### Buffer areas

Because of the wide variation across the 50 populated communities in Inuit Nunangat and the dearth of accurate social and environmental data that would be needed to conduct assessments at a neighborhood or sub-community scale the ACCVI focuses on analysis of 100-km buffers surrounding each community.

### Exposure calculation

Exposure indices were used to project characteristics of climate change that may influence future vulnerability in the Canadian Arctic. We selected three climate variables (rain, snow and temperature winter-summer) that are known to be changing in magnitude or frequency–examined through mean values and extreme indices (Supplementary Note 4; Supplementary Table 1, 2; Supplementary Fig. 1). These weather variables play a role in infrastructure exposure and potentially affect vulnerability. As an example, precipitation and winds may cause low visibility and navigability for aviation or permafrost changes and snow accumulation may impact airport operations. We also included projected sea level rise data to develop a sea level rise vulnerability model^67^. Relative sea level rise to impact ports infrastructure and communities, with links to storm surges and coastal flooding.

**Fig. 7:**
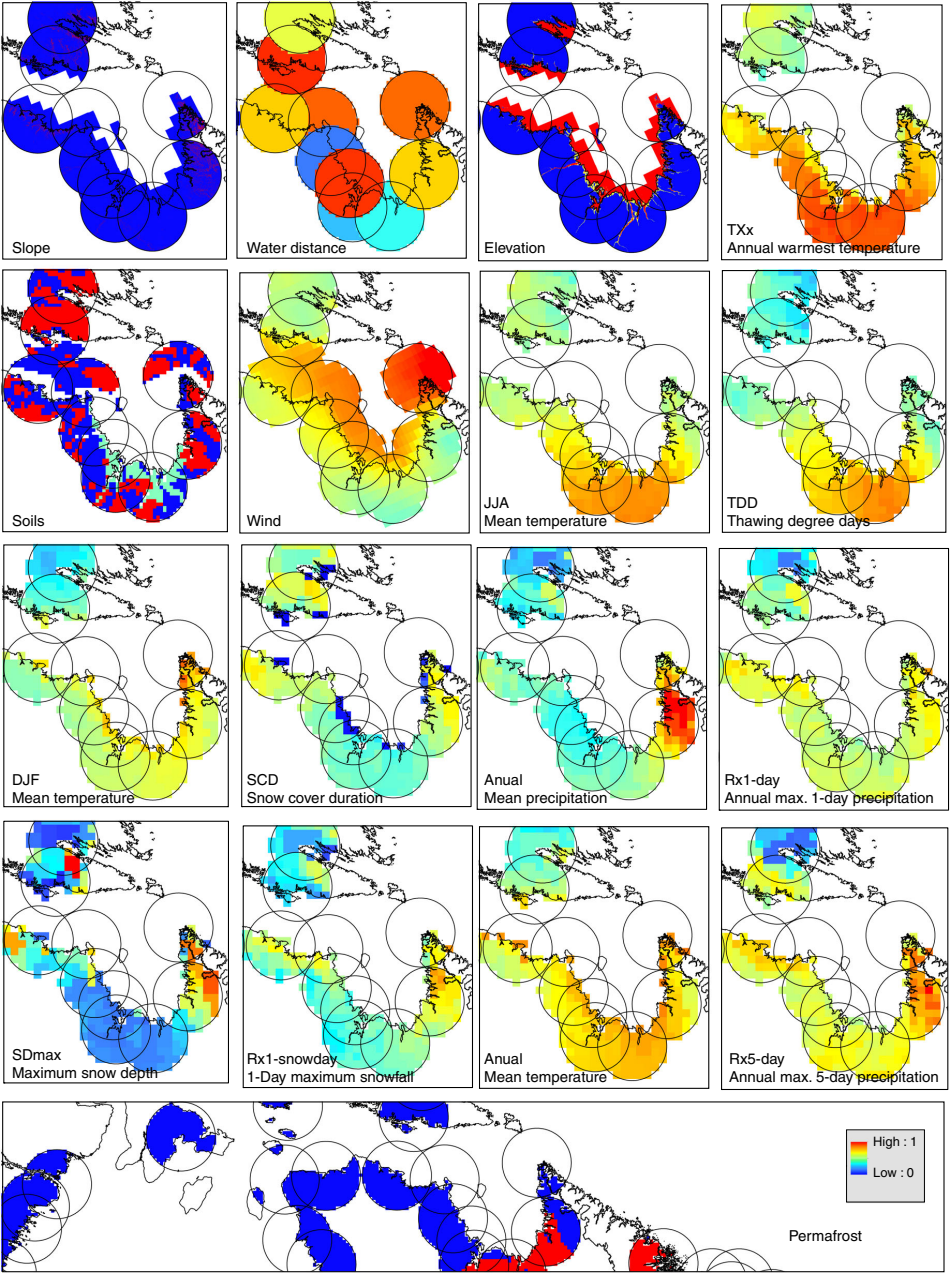
Normalized climate variables, extreme indices and physical features. Values were rescaled from 0–1 to combine with other indices

Exposure was calculated by combining climate variables and extreme indices such as temperature or precipitation projections and physical features such as permafrost, soils, elevation, slope, wind and water distance (Fig. 7). A detailed example of the exposure rain model calculation where climate variables are added to physical features can be found in Supplementary Fig. 1. All variables used in the index’s calculations were normalized from 0–1 to allow comparison.

### Exposure projection calculation

We calculated baseline and future exposure scenarios using a two-step process. First, we summed the delta value for each climate model variable at both RCP 4.5 and 8.5 scenarios in three timeframes (2040, 2070, 2100) and divided by *n* climate variables used.

2 $$\begin{array}{*{20}{c}} {\left( {\mathrm{i}} \right)\,{\mathrm{Exposure}}\,{\mathrm{\Delta }}} \\ { = \left( {\frac{{{\mathrm{\Delta }}\,x\,{\mathrm{variable}}_{2040{\mathrm{RCP}}4.5|8.5} + {\mathrm{\Delta }}\,y\,{\mathrm{variable}}_{2040{\mathrm{RCP}}4.5|8.5} + {\mathrm{\Delta }}\,z\,{\mathrm{variable}}_{2040{\mathrm{RCP}}4.5|8.5}}}{n}} \right)} \\ { = \left( {\frac{{{\mathrm{\Delta }}\,x\,{\mathrm{variable}}_{2070{\mathrm{RCP}}4.5|8.5} + {\mathrm{\Delta }}\,y\,{\mathrm{variable}}_{2070{\mathrm{RCP}}4.5|8.5} + {\mathrm{\Delta }}\,z\,{\mathrm{variable}}_{2070{\mathrm{RCP}}4.5|8.5}}}{n}} \right)} \\ { = \left( {\frac{{{\mathrm{\Delta }}\,x\,{\mathrm{variable}}_{2100{\mathrm{RCP}}4.5|8.5} + {\mathrm{\Delta }}\,y\,{\mathrm{variable}}_{2100{\mathrm{RCP}}4.5|8.5} + {\mathrm{\Delta }}\,z\,{\mathrm{variable}}_{2100{\mathrm{RCP}}4.5|8.5}}}{n}} \right)} \end{array}$$

As a second step, the resulting delta from Eq. 2 was added to the exposure baseline which included physical features. Results were rescaled considering the lowest and highest values between the baseline and the future scenarios.

3 $$\begin{array}{*{20}{c}} {\left( {{\mathrm{ii}}} \right)\,{\mathrm{Future}}\,{\mathrm{exposure}}} \\ { = {\mathrm{Exposure}}_{{\mathrm{\Delta }}\,2040{\mathrm{RCP}}4.5|8.5} + {\mathrm{Exposure}}_{{\mathrm{baseline}}\,({\mathrm{include}}\,{\mathrm{physical}}\,{\mathrm{features}})}} \\ { = {\mathrm{Exposure}}_{{\mathrm{\Delta }}\,2070{\mathrm{RCP}}4.5|8.5} + {\mathrm{Exposure}}_{{\mathrm{baseline}}\,({\mathrm{include}}\,{\mathrm{physical}}\,{\mathrm{features}})}} \\ { = {\mathrm{Exposure}}_{{\mathrm{\Delta }}\,2100{\mathrm{RCP}}4.5|8.5} + {\mathrm{Exposure}}_{{\mathrm{baseline}}\,({\mathrm{include}}\,{\mathrm{physical}}\,{\mathrm{features}})}} \end{array}$$

The future exposure value is placed at the ACCVI vulnerability equation. This operation was performed sequentially for all climate variables with variations according to the model (Supplementary Notes 7–10).

### Sensitivity indices

Sensitivity indices included Airport Sensitivity, Marine Sensitivity and Disaster Sensitivity. These were used to better assess distinct transportation sectors and better understand regional dynamics for the various transportation systems. In the vulnerability calculation Disaster Sensitivity results are combined to Airport and Marine Sensitivities composing a unique sensitivity value for airport and marine sectors (Supplementary Notes 19–23; Supplementary Figs. 6–8).

### Airport sensitivity calculation

Sensitivity of airport infrastructure was assessed using Civil Aviation Daily Occurrence Reporting system (CADORs), reported occurrences of power outages, missing weather, and average minutes that airports were closed (Supplementary Table 11). The ability of an airport to reliably provide aviation weather observations and forecasts is critical to flight planning and safe operations, particularly given the remoteness of many of the Arctic airports^41,68^. Additional information was obtained from the Canadian Flight Supplement (CFS) and Statistics Canada CANSIM database on air transportation to measure ability to land in low visibility conditions, amount of aviation traffic, runway size and lighting, and reported hazards around the airport. Regional gridded wind data was also used to assess variance between prevalent wind directions and runway heading^69,70^ (Supplementary Fig. 9).

4 $$\begin{array}{*{20}{c}} {{\mathrm{Aviation}}_{{\mathrm{sensitivity}}\,{\mathrm{index}}}} \\ { = \left( {{\mathrm{power}}\,{\mathrm{failure}}_{{\mathrm{minutes}}} + {\mathrm{missing}}\,{\mathrm{weather}}_{{\mathrm{minutes}}}} \right.} \\ {\, + \,{\mathrm{closed}}\,{\mathrm{during}}\,{\mathrm{normal}}\,{\mathrm{hours}}_{{\mathrm{minutes}}} + {\mathrm{average}}\,{\mathrm{of}}\,{\mathrm{wind}}\,{\mathrm{direction}}\,{\mathrm{off}}\,{\mathrm{runway}}\,{\mathrm{heading}}_{{\mathrm{minutes}}}} \\ {\left. {\, + {\mathrm{CFS}}_{{\mathrm{cautions}}} + {\mathrm{visibility}}\,{\mathrm{of}}\,{\mathrm{approach}}_{{\mathrm{minimum}}} + {\mathrm{descent}}\,{\mathrm{altitude}}_{{\mathrm{minimum}}}} \right) - \left( {{\mathrm{runway}}_{{\mathrm{area}}}} \right.} \\ {\left. {\, - {\mathrm{runway}}_{{\mathrm{surface}}} - {\mathrm{TAF}}_{{\mathrm{auto}}} - {\mathrm{normal}}\,{\mathrm{operation}}_{{\mathrm{hours}}} - {\mathrm{if}}\,{\mathrm{night}}\,{\mathrm{ops}}_{{\mathrm{allowed}}} - {\mathrm{IFR}}_{{\mathrm{ranking}}}} \right)} \end{array}$$

### Marine sensitivity calculation

The mechanical and navigational equipment of vessels traveling in the Canadian Arctic, as well as crews’ experience levels, varies widely. Since this is a community index, we focused on variables that are community dependent. The Marine Sensitivity Index was based on variables that represented areas of past risks and the availability of risk reducing information and resources across the region. Department of Fisheries and Oceans (DFO) provided data on locations of navigational aids (NAVTEX), electronic navigational chart coverage, reported anchoring grounds, and marine traffic were available across the region and applied. The presence of marine navigational aids and electronic navigational charts was discussed in numerous documents and by key informants as being an important factor in how safe an area is to travel through or off-load cargo in ref. ^46^. NAVTEX, which transmit information on weather and marine conditions, were seen as reducing susceptibility to incidents in the coverage areas. The amount of marine traffic in an area was seen as both a sensitivity, due to potential shoreline erosion, impacts from pollution or potential spills, and disruptions to local harvesters^27,51^:

5 $$\begin{array}{*{20}{c}} {{\mathrm{Marine}}_{{\mathrm{sensitivity}}\,{\mathrm{index}}}} \\ { = \left( {\frac{{{\mathrm{marine}}_{{\mathrm{indicents}}}}}{{{\mathrm{marine}}_{{\mathrm{traffic}}}}}} \right) - {\mathrm{ENC}}\,{\mathrm{chart}}_{{\mathrm{count}}} - {\mathrm{NAVTEX}}_{{\mathrm{presence}}} - {\mathrm{anchoring}}\,{\mathrm{grounds}}_{{\mathrm{count}}}} \end{array}$$

### Disaster sensitivity calculation

The Disaster Sensitivity Index was designed to capture present rates of search and rescues and major disasters, as well as estimate response speed from DOD for a major event.

Search and rescue rates were calculated based on data from Public Safety Canada’s Knowledge Management System for years 2013 and 2014. More recent data was not available. Using ArcGIS spatial analysis tools, each event was joined with the closest proximal community. Incidence rates were calculated using Statistics Canada 2016 population data. The majority of search and rescue events were related to subsistence hunting and traditional travel on the land^71^. High search and rescue rates were seen as being an indicator of sensitivity for a variety of reasons. Research has demonstrated that communities with high search and rescue rates likely place high demands on emergency volunteers in the community and have high volunteer burnout rates^29,72^. There is also likely a connection between lower traditional knowledge levels and/or higher community hazards and elevated search and rescue rates.

Data on disasters near or in communities was also used as an indicator of sensitivity. Similar to tracking of marine incidents, it was assumed that communities that had previous disasters have more hazards. While it is possible that these communities have learned and adapted to reduce risks, the tracking of past disasters is often used to assess future risks^57,68,73^.

Public Safety maintains a database of qualifying disasters across Canada. To qualify an event has to meet one of more of the following criteria. First, 10 or more people killed; Second, 100 or more people affected/injured/infected/evacuated or homeless; Third, an appeal for national/international assistance; Fourth, historical significance; or Fifth, significant damage/interruption of normal processes such as that the community affected cannot recover on its own. Between 1900 and 2018, there were 6 qualifying disasters reported in Inuit Nunangat^74^. Three events were due to flooding, one due to storm surge, one due to an avalanche, and one due to a major aviation disaster.

Response to disasters and search and rescue needs across Inuit Nunangat is usually handled by local and territorial/provincial resources. However, response time by federal partners is important factor in sensitivity to large scale disasters. It is assumed that the longer a community would have to wait for assistance during a major disaster or search and rescue operation, the higher the probability of damage and life loss^12^. Additionally, response time to disasters is a recommended indicator in the Expert Panel on Climate Change Adaptation and Resilience Results Report^12^. We chose to look at response times and costs for the designated SAR assets CC-130, CH149, and CH-146. While CC-138s are used, they are not in a designated SAR Wing. Data for aircraft performance was obtained from the RCAF website and reports^75^. Variables used to calculate response time were our best estimates given publicly accessible data.

6 $$\begin{array}{*{20}{c}} {{\mathrm{Disaster}}_{{\mathrm{sensitivity}}\,{\mathrm{index}}}} \\ \begin{array}{l} = \left( {{\mathrm{search}}\,{\mathrm{and}}\,{\mathrm{rescue}}_{{\mathrm{rate}}} + {\mathrm{qualifying}}\,{\mathrm{disasters}}_{{\mathrm{number}}} + {\mathrm{RCAF}}\,{\mathrm{to}}\,{\mathrm{reach}}\,{\mathrm{community}}_{{\mathrm{combined}}\,{\mathrm{hours}}}} \right)\\ \quad \quad \quad \quad \quad \quad - {\mathrm{physicians}}_{{\mathrm{per}}\,{\mathrm{capita}}}\end{array} \end{array}$$

### Adaptive capacity calculation

Adaptive capacity was estimated using data from Statistics Canada and the Indigenous and Northern Affairs Canada (INAC) Community Wellbeing Index (CWI). This combination of data sources allowed us to weight socioeconomic conditions, housing conditions, education attainment, traditional knowledge, and demographics (Supplementary Fig. 10).

Developed by INAC, the CWI uses Statistics Canada census data to score communities based on socio-economic wellbeing, education attainment, labor force activities, income, and housing. Aggregated 2011 CWI scores were used in this study as the 2016 CWI had not yet been published by INAC. The CWI score was missing for 3 communities, which were given the mean regional score. The average CWI score across the region was 61, while the standard deviation was 11.7. Iqaluit had the highest CWI score, while Naujaat had the lowest reported CWI score.

To supplement the CWI, we used data from the 2016 Canadian census. We looked at education attainment by using percent of high school diplomas, percent of trades certificates, and percent of the community that spoke Inuktitut. In combining western markers of education attainment and one element of Inuit Traditional Knowledge (language), we hoped to better reflect the diverse types of knowledge that build resilience^16^. However, we acknowledge that an individual can have high levels of Traditional Knowledge without speaking Inuktitut and someone can have lower levels of Traditional Knowledge and speak Inuktitut. We also examined the percent of the community that was new immigrants as a new immigrant population likely has fewer land skills, limited knowledge of the Arctic, and no Inuit Traditional Knowledge. The percent of the population that was younger than 14 and older than 65 was also assessed. Knowing that language is only one of the many aspects of Inuit identity and culture, we strongly agree that future work needs to develop ways of measuring indicators for Inuit Traditional Knowledge for use in developing indices like those developed here.

The average community percent population that had graduated from high school was reported at 10%. The community with the highest graduation rate was Ulukhaktok (19%) and the lowest was Taloyoak (4%). The average community percent population that had a trade certificate was 4%. Postville had the highest percent of individuals with a trade certificate (14%) while five communities reported 0%.

Communities with the highest percentage of recent immigrants were Quaqtaq, Iqaluit, Inuvik, and Coral Harbor. Statistics Canada defines recent immigrant as individuals who have immigrated to Canada between 2011 and 2016. Naujaat had the youngest population with the median age of 18 and 43.1% of the population under 14. Postville had the oldest population with an average age of 41.2 and over 17.1% of the population over 65.

7 $$\begin{array}{*{20}{c}} {{\mathrm{Adaptive}}\,{\mathrm{capacity}}_{{\mathrm{index}}}} \\ \begin{array}{l} = \left( {{\mathrm{CWI}} + {\mathrm{speaking}}\,{\mathrm{Inuktitute}}\,{\mathrm{at}}\,{\mathrm{home}}\% + {\mathrm{high}}\,{\mathrm{school}}\,{\mathrm{diploma}}\% + {\mathrm{trade}}\,{\mathrm{certificate}}\% } \right)\\ \quad \quad \quad \quad \quad \quad \quad - \left( {{\mathrm{recent}}\,{\mathrm{immigrants}}\% - {\mathrm{under}}\,14\% - {\mathrm{over}}\,65\% } \right)\end{array} \end{array}$$

### ACCVI vulnerability models

**Fig. 8:**
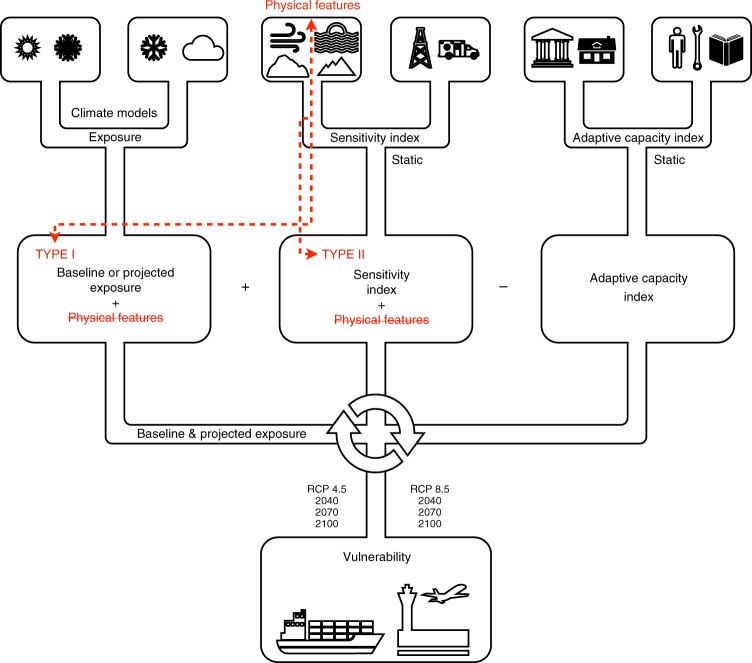
Vulnerability model type I was calculated for all 50 communities for present, 2040, 2070, and 2100 timeframes under RCP 4.5 and 8.5 scenarios. Model was run using climate variables and extreme indices and for each sensitivity indices pairing (airport-disasters or marine-disasters). Under the Type I model, exposure projections and physical features were calculated together, then added to sensitivity (marine or airport)

Four vulnerability hazards (rain, snow, winter-summer temperatures, and sea level rise) included Type I model and a Multiplicative equation. The model was structured to identify existing spatial trends in vulnerability. Societal factors are emphasized over climate and physical features, and marine and airport sensitivity and adaptive capacity are calculated separately from exposure. This resulted in a quasi-weight of two-thirds weight on societal factors, while climate and physical features were allotted one-third of the weight. In this model, climate and physical features were coupled then added to airport or marine sensitivity (Fig. 8).

Outputs were assessed and verified by cross comparison with previous research and data from key informant interviews, indicating that Type I model was far more representative of the current vulnerabilities outlined in research and reports. Further, we found that the Type I model produced projections that were more congruent with some anticipated changes described by key informants and most in line with contextual vulnerability approach.

More information on the calibration phase and the Type II model can be found in the Supplementary Notes 17, 19 and 20 (Type II results are not described in this article).

### ACCVI vulnerability equations

The additive Eq. 9 is a raw version of the original vulnerability equation which defines how vulnerability is numerically calculated (as described in Eq. 1). Eq. 8 is a multiplicative version of Eq. 9 which has been used in similar frameworks assessing climate change vulnerability indices at large scales^44,45,76^. The multiplicative version assumes that exposure and sensitivity create an impact, and one unit of this impact can be offset by one unit of adaptive capacity.

**Multiplicative**

8 $$\begin{array}{*{20}{c}} {{\mathrm{Vulnerability}}_X} \\ { = \left( {\frac{{\left( {{\mathrm{Exposure}}_{{\mathrm{baseline}}\,{\mathrm{or}}\,{\mathrm{projection}}} + {\mathrm{Sensitivity}}_{{\mathrm{airport}}\,{\mathrm{or}}\,{\mathrm{marine}}}} \right)}}{2}} \right) \ast \left( {0.50 + \frac{{\left( {1 - {\mathrm{Adaptive}}\,{\mathrm{capacity}}} \right)}}{2}} \right)} \end{array}$$

**Additive**

9 $$\begin{array}{*{20}{c}} {{\mathrm{Vulnerability}}_X} \\ { = \left( {{\mathrm{Exposure}}_{{\mathrm{baseline}}\,{\mathrm{or}}\,{\mathrm{projection}}} + {\mathrm{Sensitivity}}_{{\mathrm{airport}}\,{\mathrm{or}}\,{\mathrm{marine}}}} \right) - {\mathrm{Adaptive}}\,{\mathrm{capacity}}} \end{array}$$

Both equations designated a magnitude of increase or decrease in vulnerability depending on the RCP projection being performed. When Eq. 9 was applied to the method, output values scaled differently, extrapolating and inflating the indices beyond the 0–1 scale (for both baseline and future scenarios) not aligning with case study and field observations data. Eq. 8 correctly normalized values with results ranging from 0–1. For this reason, we only discuss and present results from the multiplicative equation.

Calculations results and figures for each exposure, vulnerability and increment projections (rain, snow, temperatures and sea level rise) under the multiplicative equation can be found in Supplementary Data 1, 3 and 4 and Supplementary Figs. 2, 3.

### Increment equation percentage increase

Future vulnerability maps, at times, only show slight regional differences. For example, if in future periods the same grid point had a weak increase, the vulnerability class will remain visually the same as in the baseline, or if a grid point belongs to some of the extreme classes of vulnerability (too low or too high) and this same grid point vulnerability has changed, it will remain visually in the same class, independent of the magnitude of change. This type of occurrence is undesirable for depicting future periods particularly in highly vulnerable regions. In these cases, it would not be possible to identify how the vulnerability will intensify. Therefore, we produced maps of vulnerability increase which are represented as a fractional change (Supplementary Fig. 4).

10 $$\begin{array}{*{20}{c}} {{\mathrm{Increment}}_{{\mathrm{percentage}}\,(\% )}} \\ { = \left( {\frac{{{\mathrm{future}}\,{\mathrm{vulnerability}}_{{\mathrm{airport}}\,{\mathrm{or}}\,{\mathrm{marine}}} - {\mathrm{baseline}}\,{\mathrm{vulnerability}}_{{\mathrm{airport}}\,{\mathrm{or}}\,{\mathrm{marine}}}}}{{{\mathrm{baseline}}\,{\mathrm{vulnerability}}_{{\mathrm{airport}}\,{\mathrm{or}}\,{\mathrm{marine}}}}}} \right)} \end{array}$$

Finally, when comparing mean and median results values for the exposure, vulnerability and increment equations and finding them alike for each case we proceed and present the mean result values for all models.

## Supplementary information

Supplementary Information

Description of Additional Supplementary Files

Supplementary Data 1

Supplementary Data 2

Supplementary Data 3

Supplementary Data 4

## Data Availability

The datasets generated during the calibration phases and/or analyzed during the current study, if not in the Supplementary Information, are available from the corresponding author on reasonable request.
