# Supplementary material for: An integrative climate change vulnerability index for Arctic aviation and marine transportation
Source: Nat Commun. 2019 Jun 13;10:2596. doi: 10.1038/s41467-019-10347-1 (PMC6565733; doi:10.1038/s41467-019-10347-1)
Supplement: Supplementary file 1 — Supplementary Information [file 41467_2019_10347_MOESM1_ESM.pdf]

Supplementary Information

An Integrative Climate Change Vulnerability Index for Arctic Aviation and Marine Transportation

by Debortoli et al.

Supplementary Figure 1. Rain model exposure baseline calculation example. Rain extreme indices (Rx1-day and Rx5-day) and mean annual precipitation are combined to physical features (wind, slope, soils and water distance) to assess rain exposure for the communities. Such method was also applied to assess other models considering its specificities.

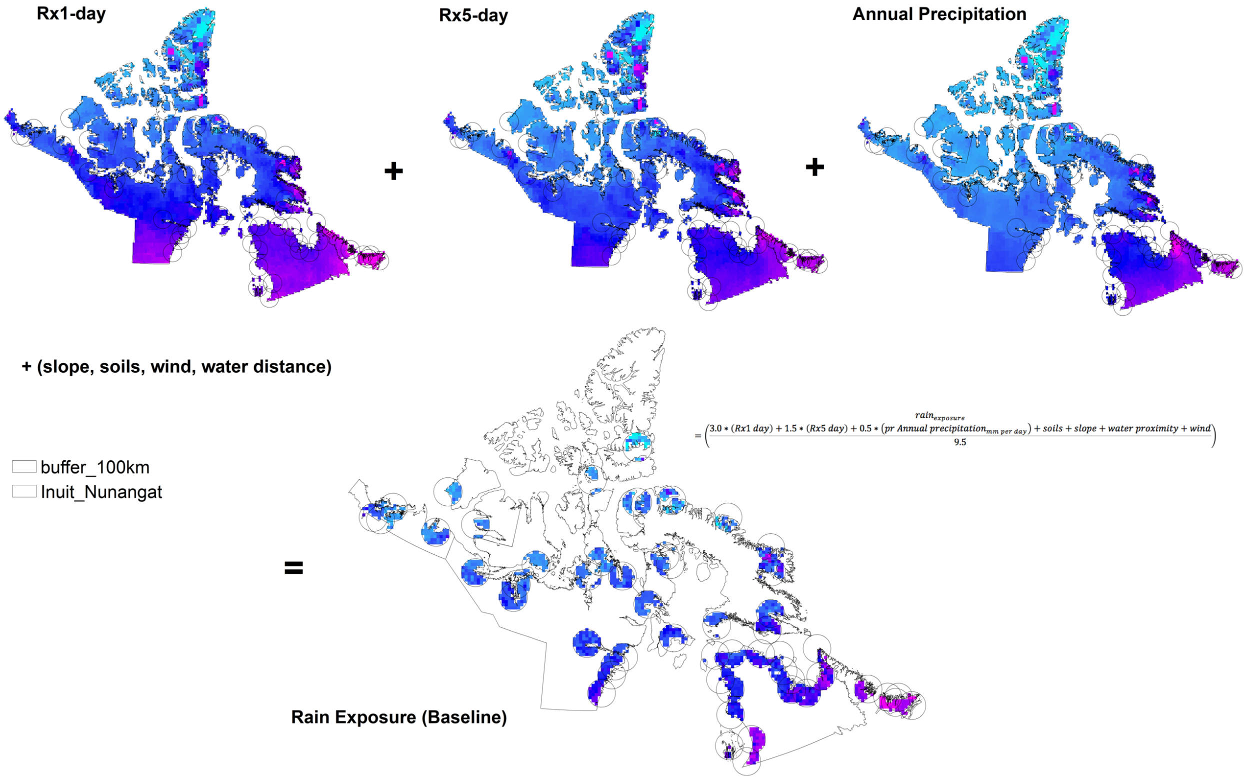

Supplementary Figure 2.

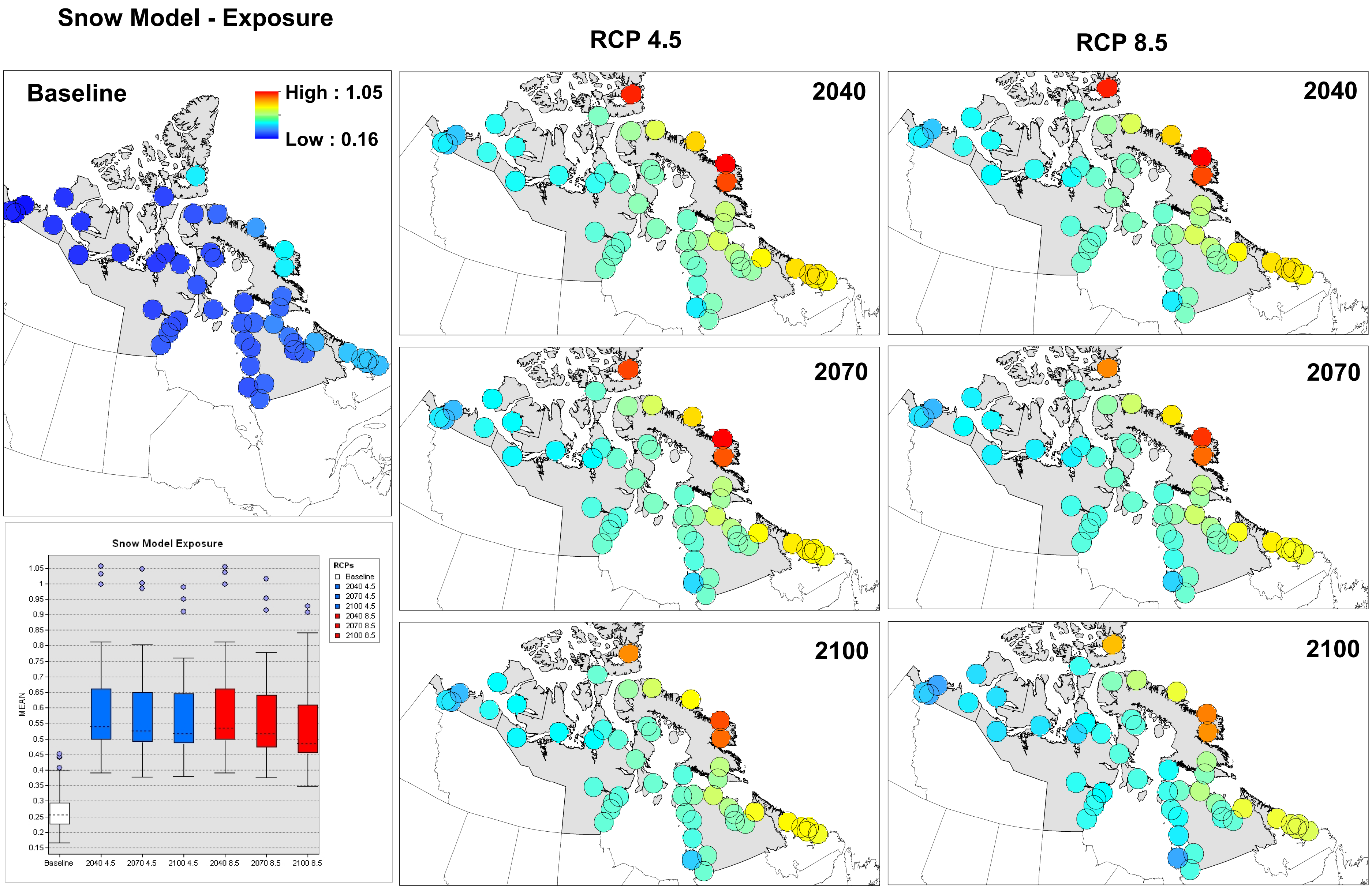

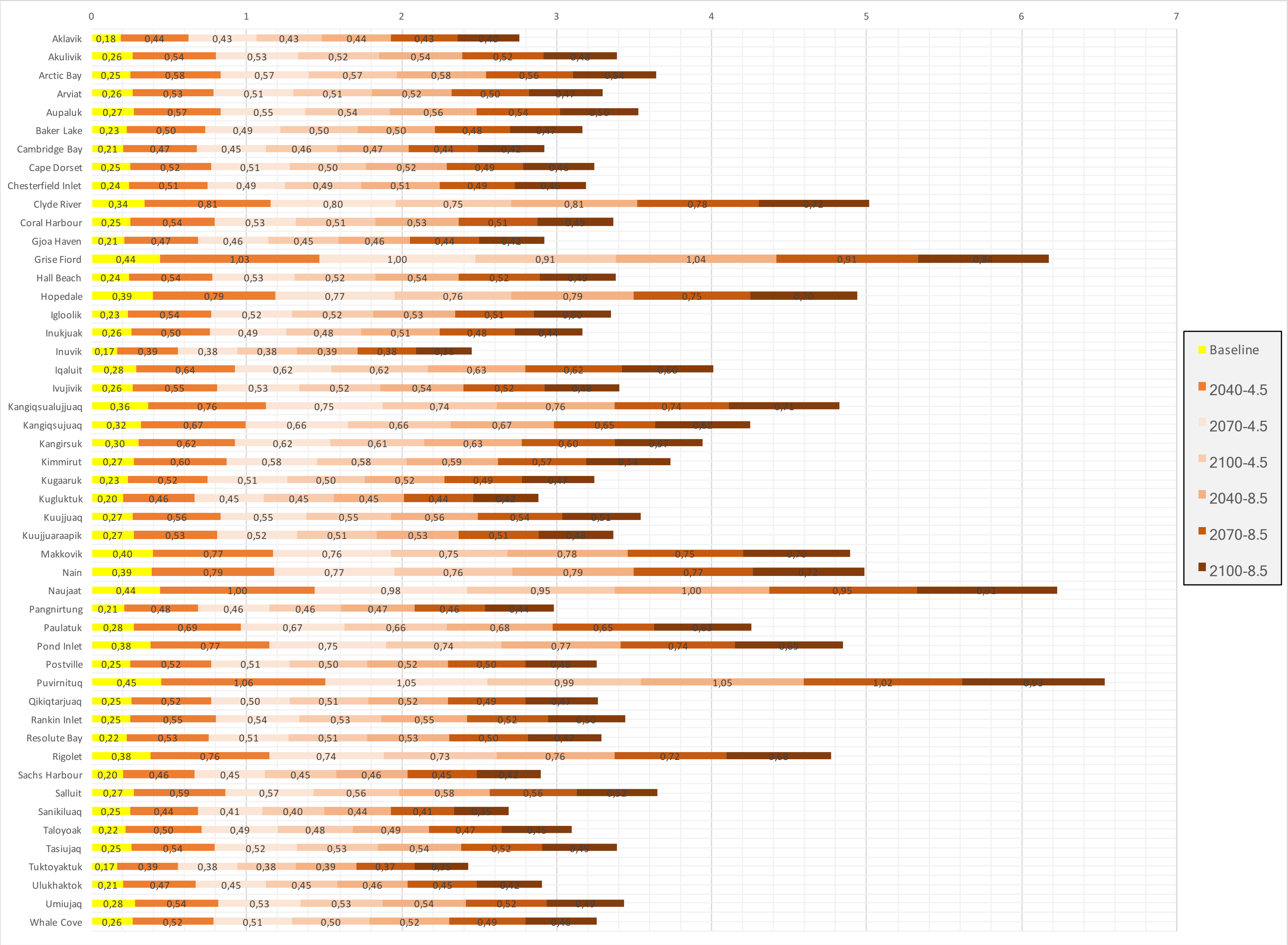
0
1
2
3
4
5
6
7
Aklavik
Akulivik
Arctic Bay
Arviat
Aupaluk
Baker Lake
Cambridge Bay
Cape Dorset
Chesterfield Inlet
Clyde River
Coral Harbour
Gjoa Haven
Grise Fiord
Hall Beach
Hopedale
Igloolik
Inukjuak
Inuvik
Iqaluit
Ivujivik
Kangiqsualujjuaq
Kangiqsujuaq
Kangirsuk
Kimmirut
Kugaaruk
Kugluktuk
Kuujjuaq
Kuujjuaraapik
Makkovik
Nain
Naujaat
Pangnirtung
Paulatuk
Pond Inlet
Postville
Puvirnituq
Qikiqtarjuaq
Rankin Inlet
Resolute Bay
Rigolet
Sachs Harbour
Salluit
Sanikiluaq
Taloyoak
Tasiujaq
Tuktoyaktuk
Ulukhaktok
Umiujaq
Whale Cove
Baseline
2040-4.5
2070-4.5
2100-4.5
2040-8.5
2070-8.5
2100-8.5

Temperature Winter Model - Exposure

RCP 4.5 | RCP 8.5

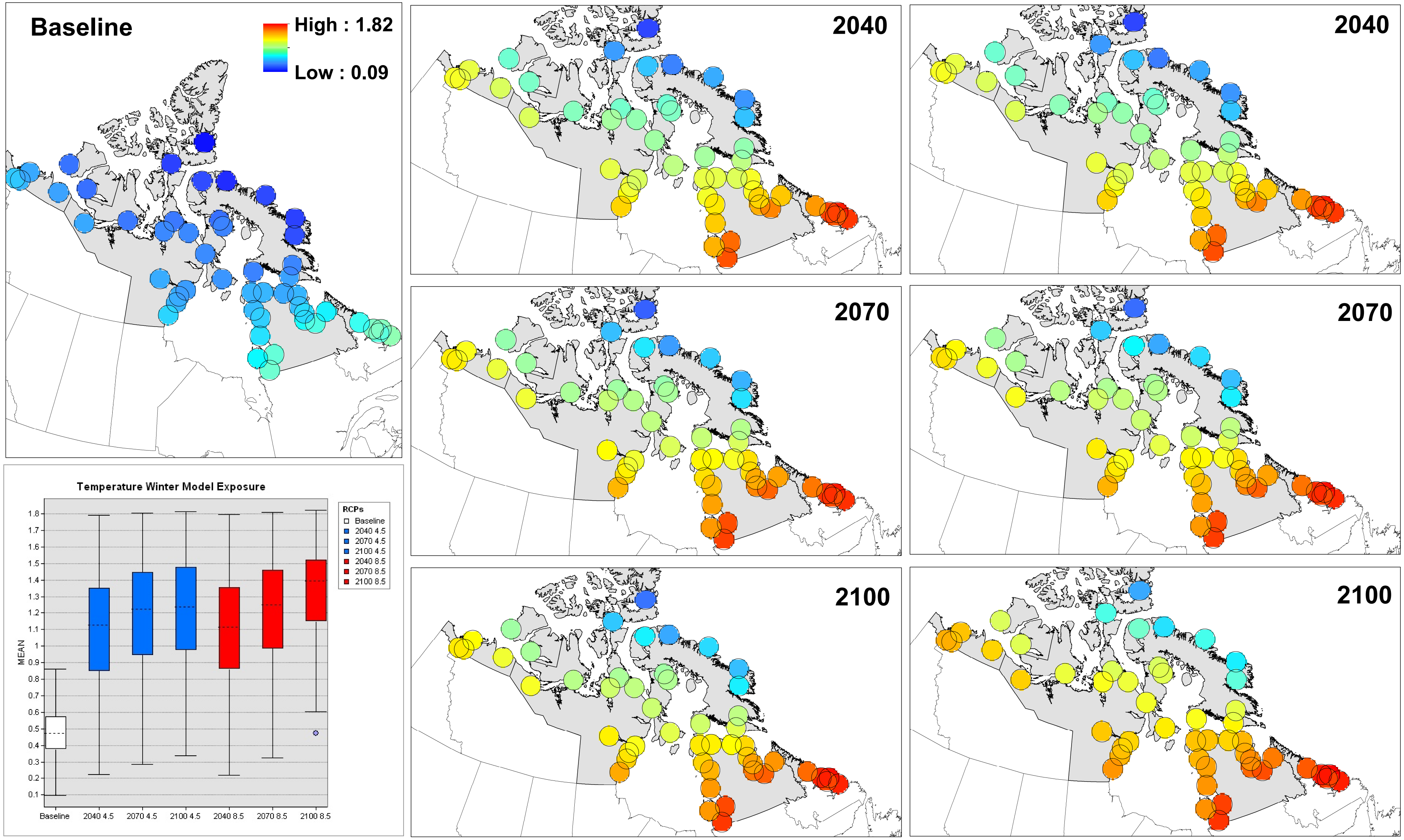

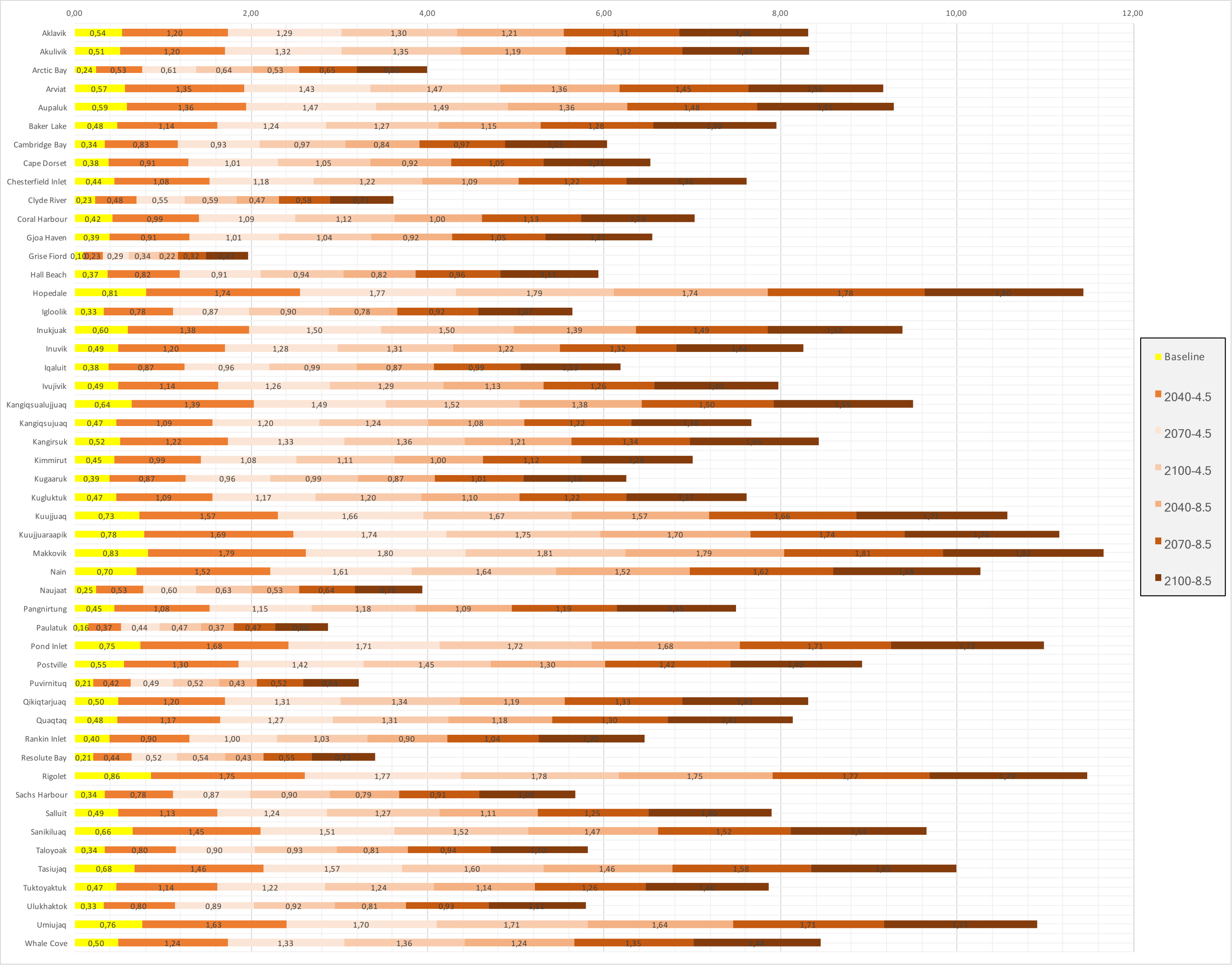
0,00
2,00
4,00
6,00
8,00
10,00
12,00
Aklavik
Akulivik
Arctic Bay
Arviat
Aupaluk
Baker Lake
Cambridge Bay
Cape Dorset
Chesterfield Inlet
Clyde River
Coral Harbour
Gjoa Haven
Grise Fiord
Hall Beach
Hopedale
Igloolik
Inukjuak
Inuvik
Iqaluit
Ivujivik
Kangiqsualujjuaq
Kangiqsujuaq
Kangirsuk
Kimmirut
Kugaaruk
Kugluktuk
Kuujjuaq
Kuujjuaraapik
Makkovik
Nain
Naujaat
Pangnirtung
Paulatuk
Pond Inlet
Postville
Puvirnituq
Qikiqtarjuaq
Quaqtaq
Rankin Inlet
Resolute Bay
Rigolet
Sachs Harbour
Salluit
Sanikiluaq
Taloyoak
Tasiujaq
Tuktoyaktuk
Ulukhaktok
Umiujaq
Whale Cove
Baseline
2040-4.5
2070-4.5
2100-4.5
2040-8.5
2070-8.5
2100-8.5

Temperature Summer Model - Exposure

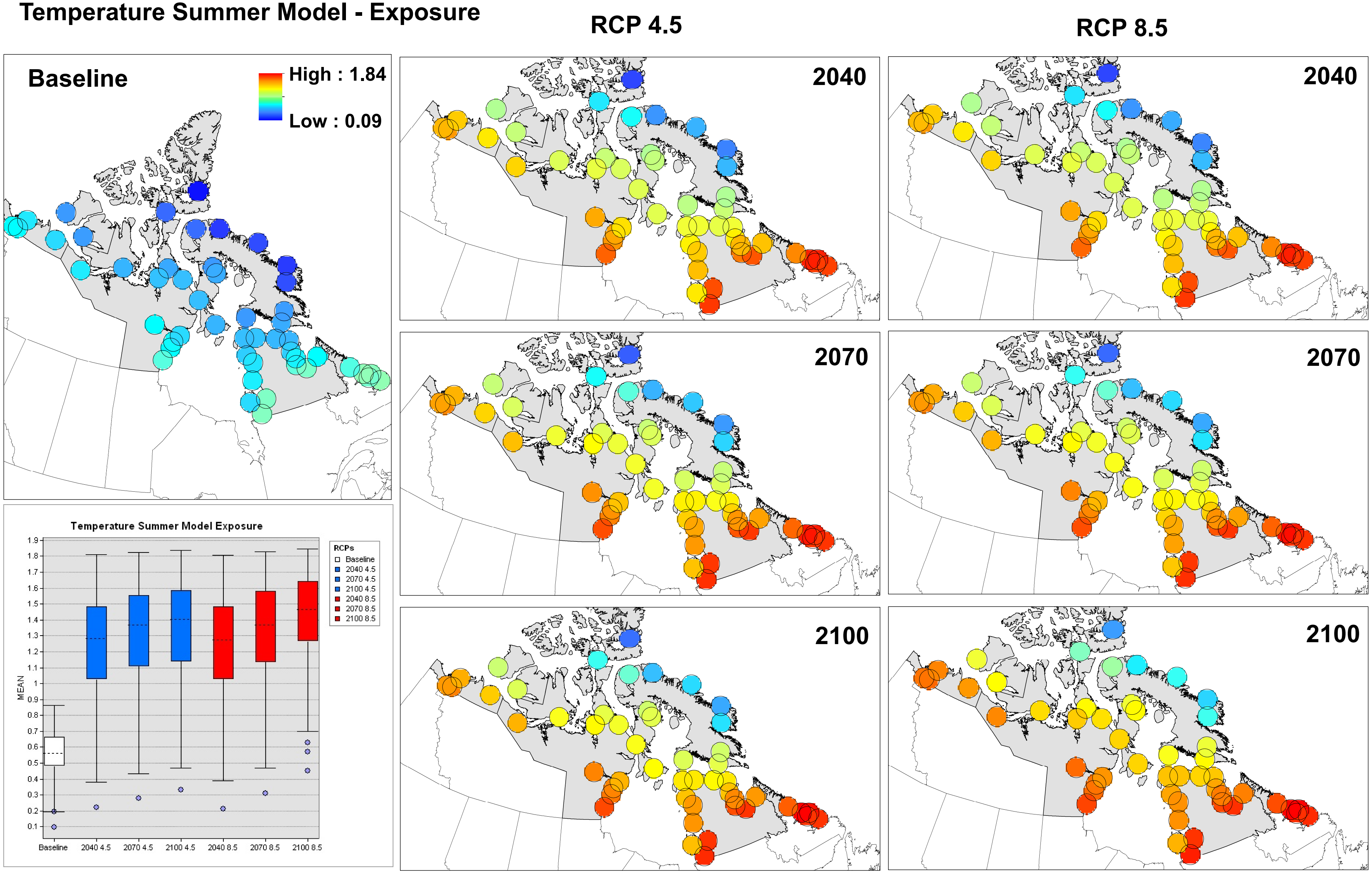

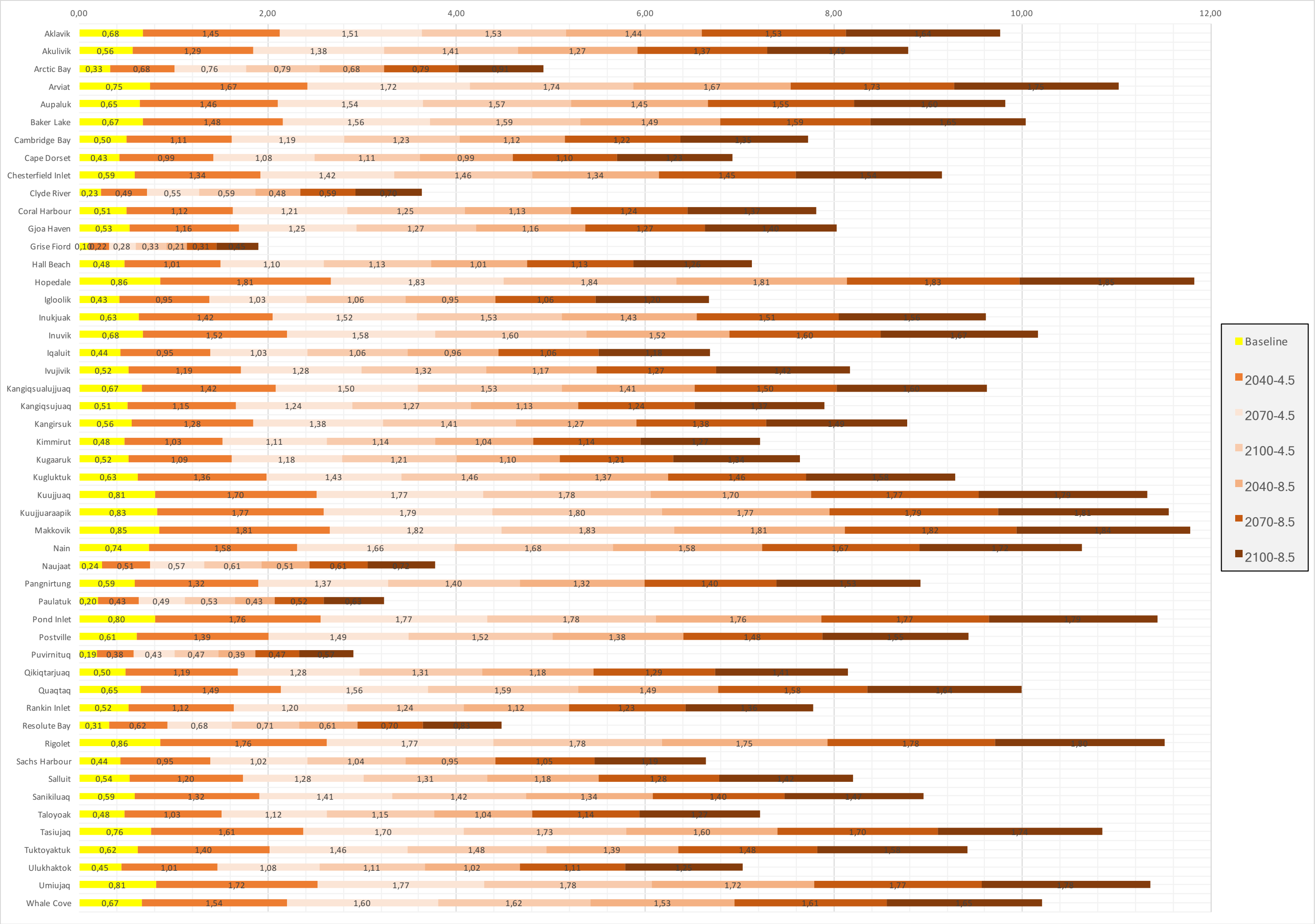
0,00
2,00
4,00
6,00
8,00
10,00
12,00
Aklavik
Akulivik
Arctic Bay
Arviat
Aupaluk
Baker Lake
Cambridge Bay
Cape Dorset
Chesterfield Inlet
Clyde River
Coral Harbour
Gjoa Haven
Grise Fiord
Hall Beach
Hopedale
Igloolik
Inukjuak
Inuvik
Iqaluit
Ivujivik
Kangiqsualujjuaq
Kangiqsujuaq
Kangirsuk
Kimmirut
Kugaaruk
Kugluktuk
Kuujjuaq
Kuujjuaraapik
Makkovik
Nain
Naujaat
Pangnirtung
Paulatuk
Pond Inlet
Postville
Puvirnituq
Qikiqtarjuaq
Quaqtaq
Rankin Inlet
Resolute Bay
Rigolet
Sachs Harbour
Salluit
Sanikiluaq
Taloyoak
Tasiujaq
Tuktoyaktuk
Ulukhaktok
Umiujaq
Whale Cove
Baseline
2040-4.5
2070-4.5
2100-4.5
2040-8.5
2070-8.5
2100-8.5

Sea Level Model Airport Exposure

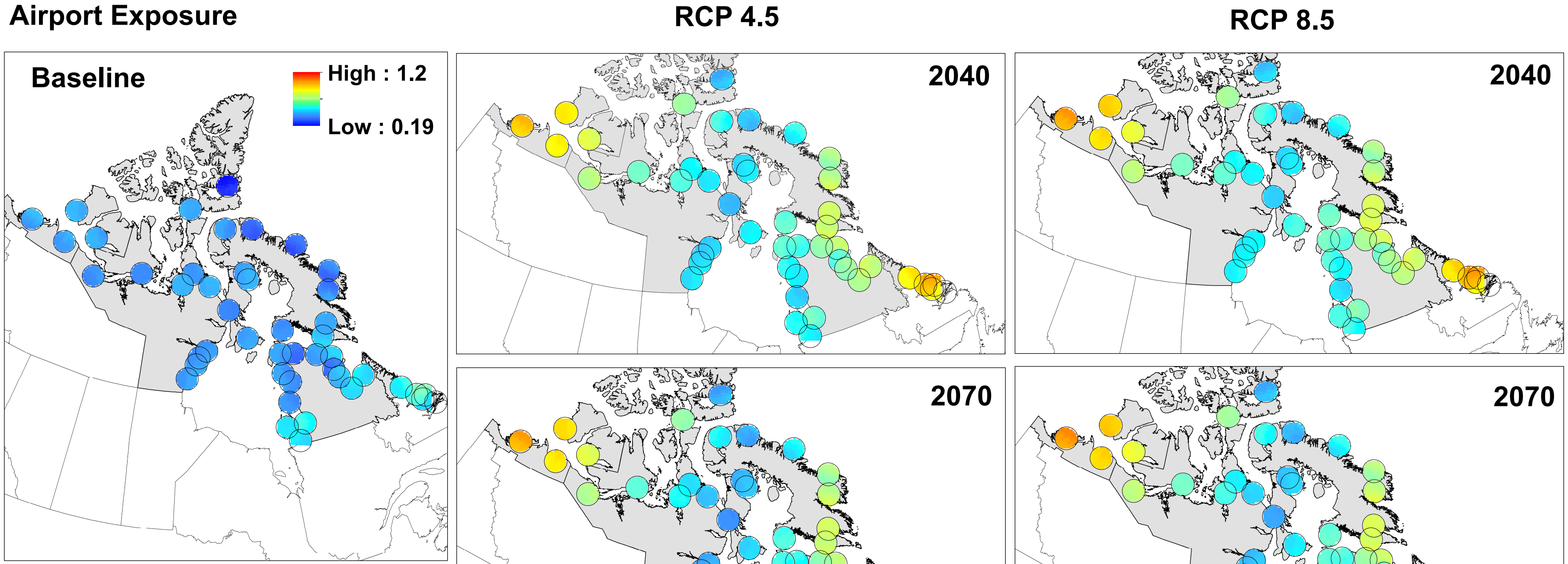

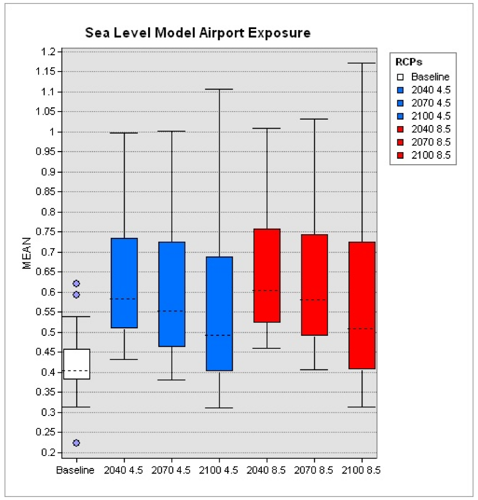

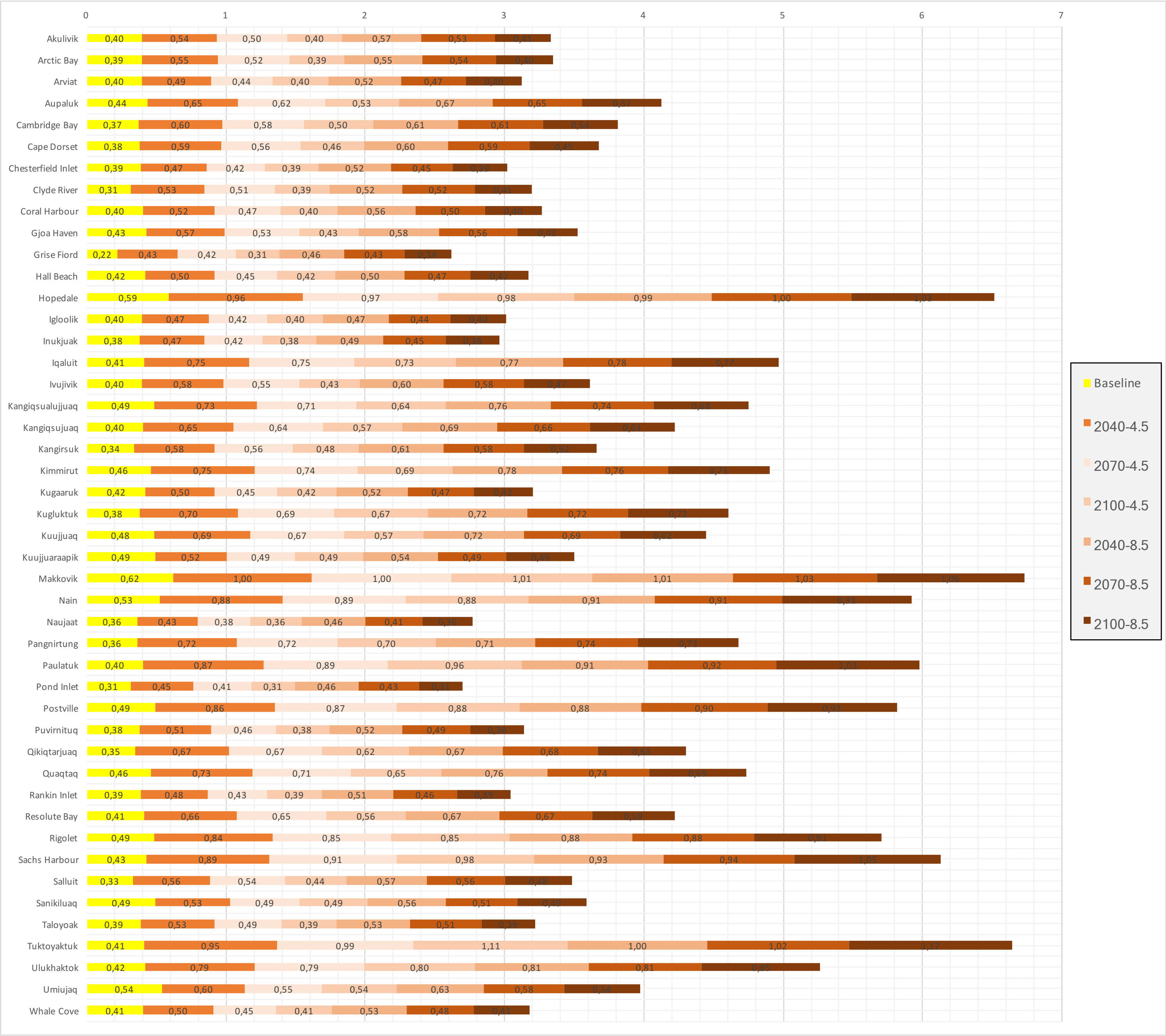
0
1
2
3
4
5
6
7
Akulivik
Arctic Bay
Arviat
Aupaluk
Cambridge Bay
Cape Dorset
Chesterfield Inlet
Clyde River
Coral Harbour
Gjoa Haven
Grise Fiord
Hall Beach
Hopedale
Igloolik
Inukjuak
Iqaluit
Ivujivik
Kangiqsualujjuaq
Kangiqsujuaq
Kangirsuk
Kimmirut
Kugaaruk
Kugluktuk
Kuujjuaq
Kuujjuaraapik
Makkovik
Nain
Naujaat
Pangnirtung
Paulatuk
Pond Inlet
Postville
Puvirnituq
Qikiqtarjuaq
Quaqtaq
Rankin Inlet
Resolute Bay
Rigolet
Sachs Harbour
Salluit
Sanikiluaq
Taloyoak
Tuktoyaktuk
Ulukhaktok
Umiujaq
Whale Cove
Baseline
2040-4.5
2070-4.5
2100-4.5
2040-8.5
2070-8.5
2100-8.5

Sea Level Model
Marine Exposure

RCP 4.5 | **RCP 8.5**

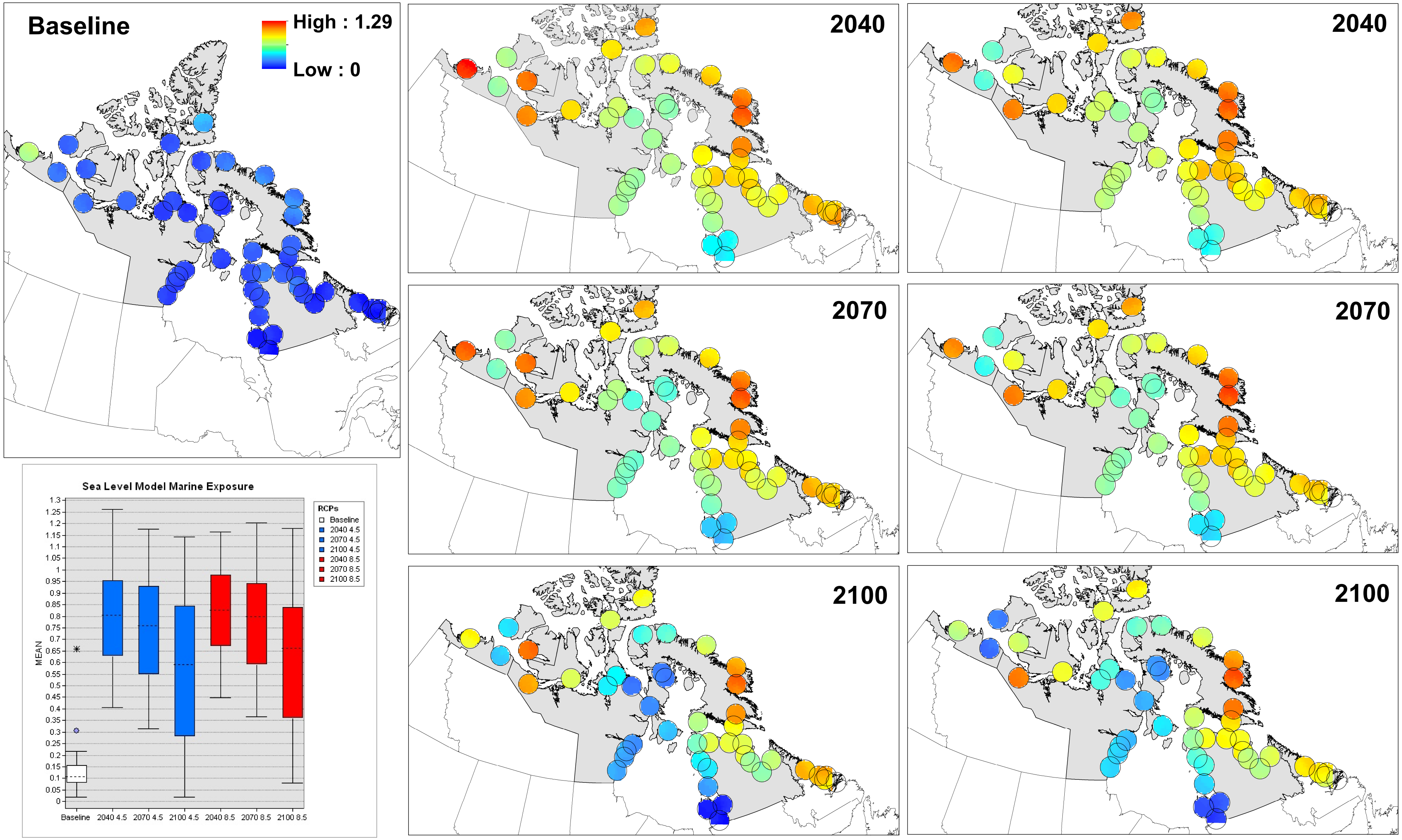

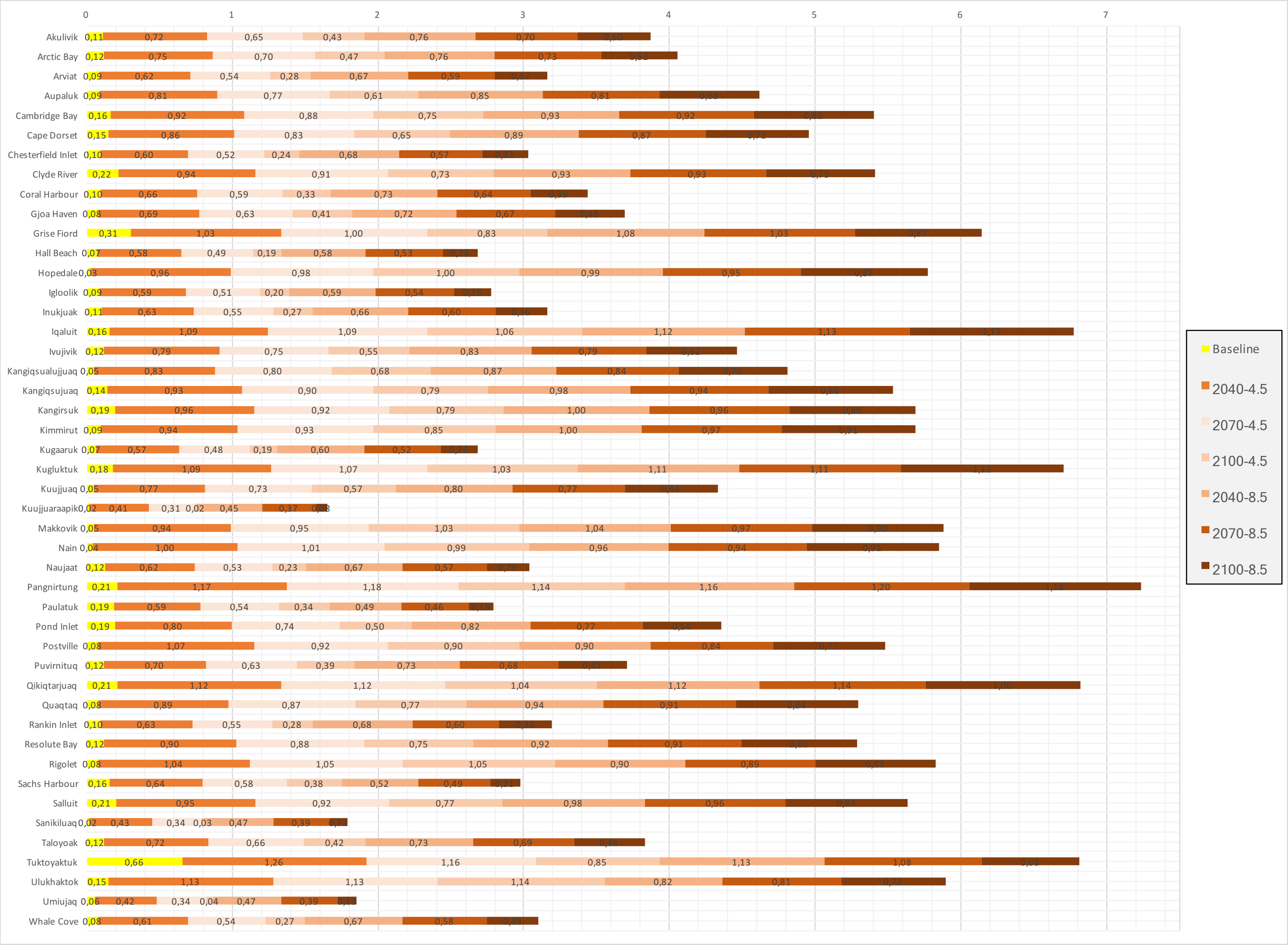
0
1
2
3
4
5
6
7
Akulivik
Arctic Bay
Arviat
Aupaluk
Cambridge Bay
Cape Dorset
Chesterfield Inlet
Clyde River
Coral Harbour
Gjoa Haven
Grise Fiord
Hall Beach
Hopedale
Igloolik
Inukjuak
Iqaluit
Ivujivik
Kangiqsualujjuaq
Kangiqsujuaq
Kangirsuk
Kimmirut
Kugaaruk
Kugluktuk
Kuujjuaq
Kuujjuaraapik
Makkovik
Nain
Naujaat
Pangnirtung
Paulatuk
Pond Inlet
Postville
Puvirnituq
Qikiqtarjuaq
Quaqtaq
Rankin Inlet
Resolute Bay
Rigolet
Sachs Harbour
Salluit
Sanikiluaq
Taloyoak
Tuktoyaktuk
Ulukhaktok
Umiujaq
Whale Cove
Baseline
2040-4.5
2070-4.5
2100-4.5
2040-8.5
2070-8.5
2100-8.5

Supplementary Figure 3.

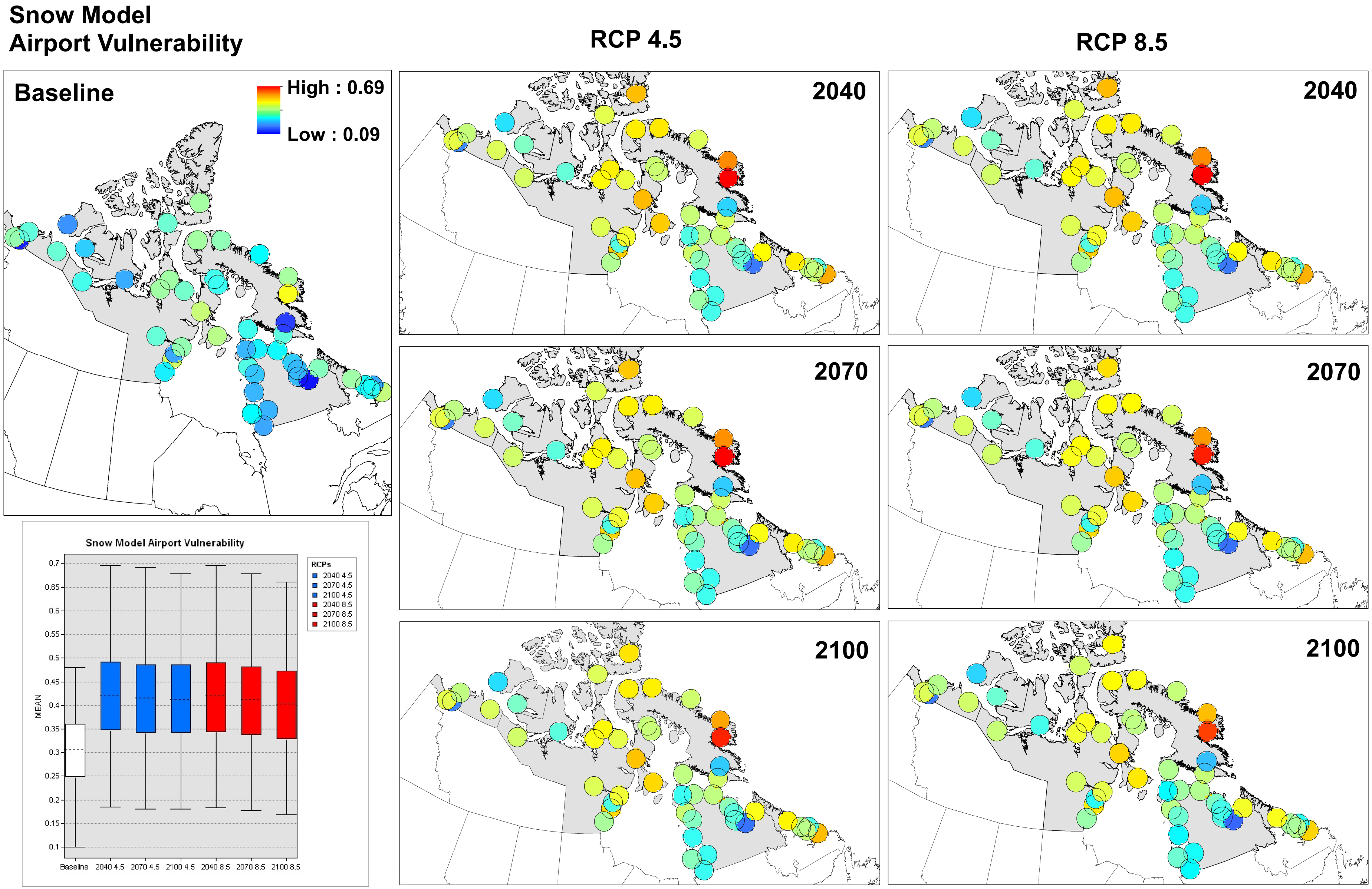

Snow Model
Marine Vulnerability

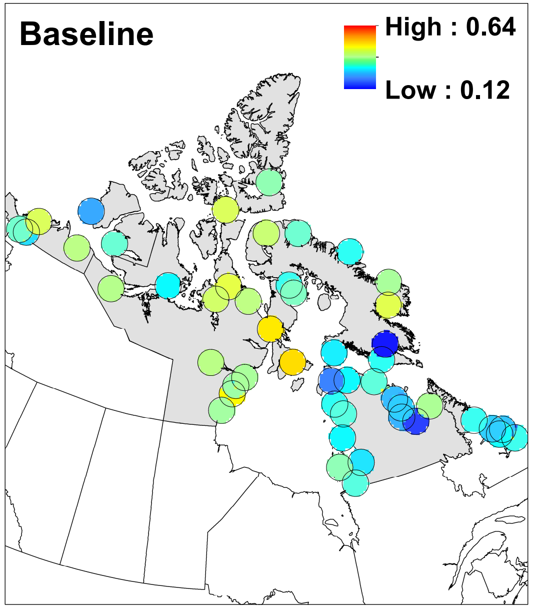

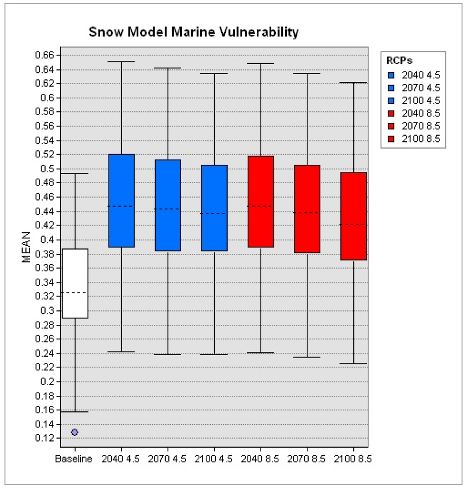

RCP 4.5 RCP 8.5

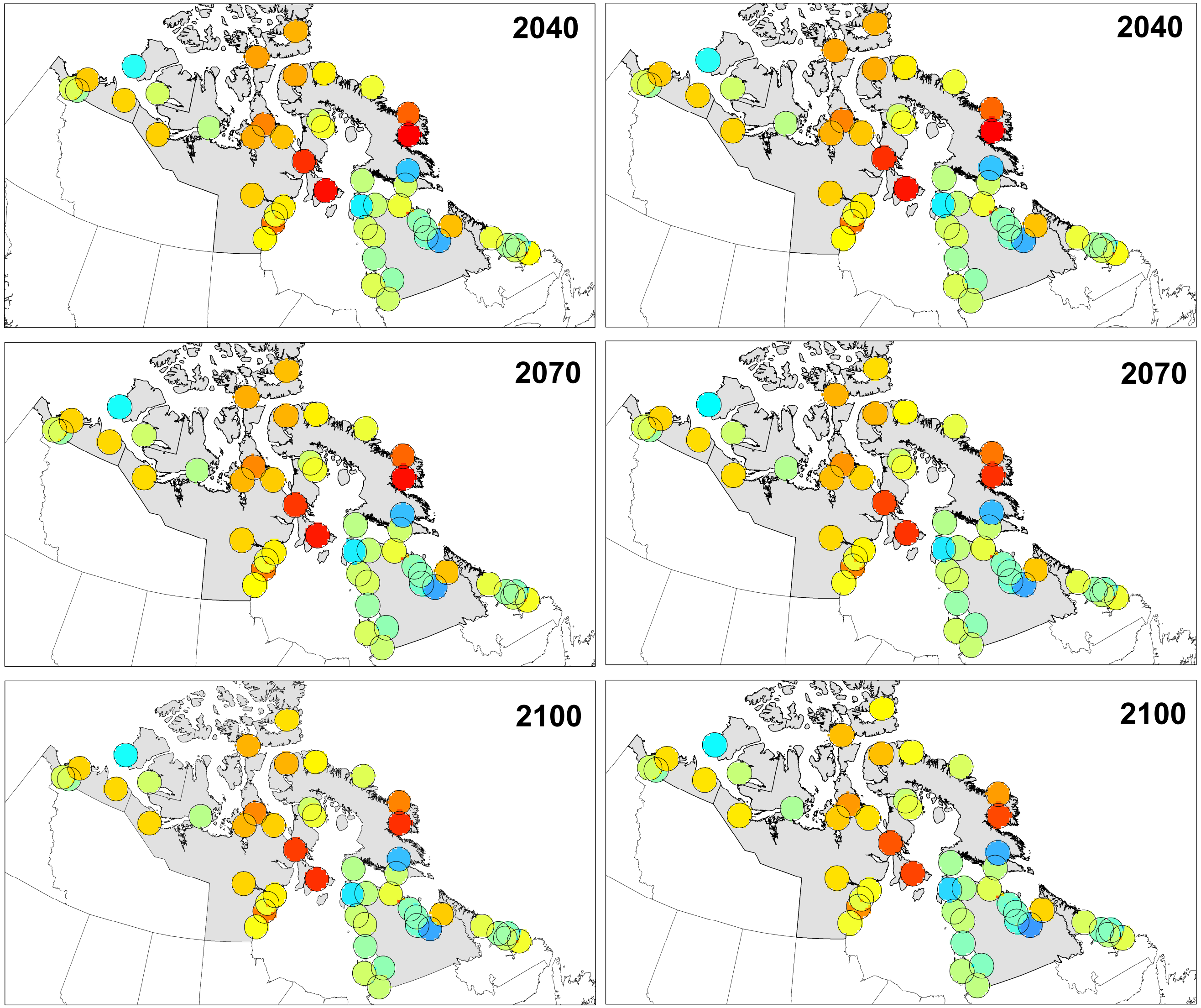

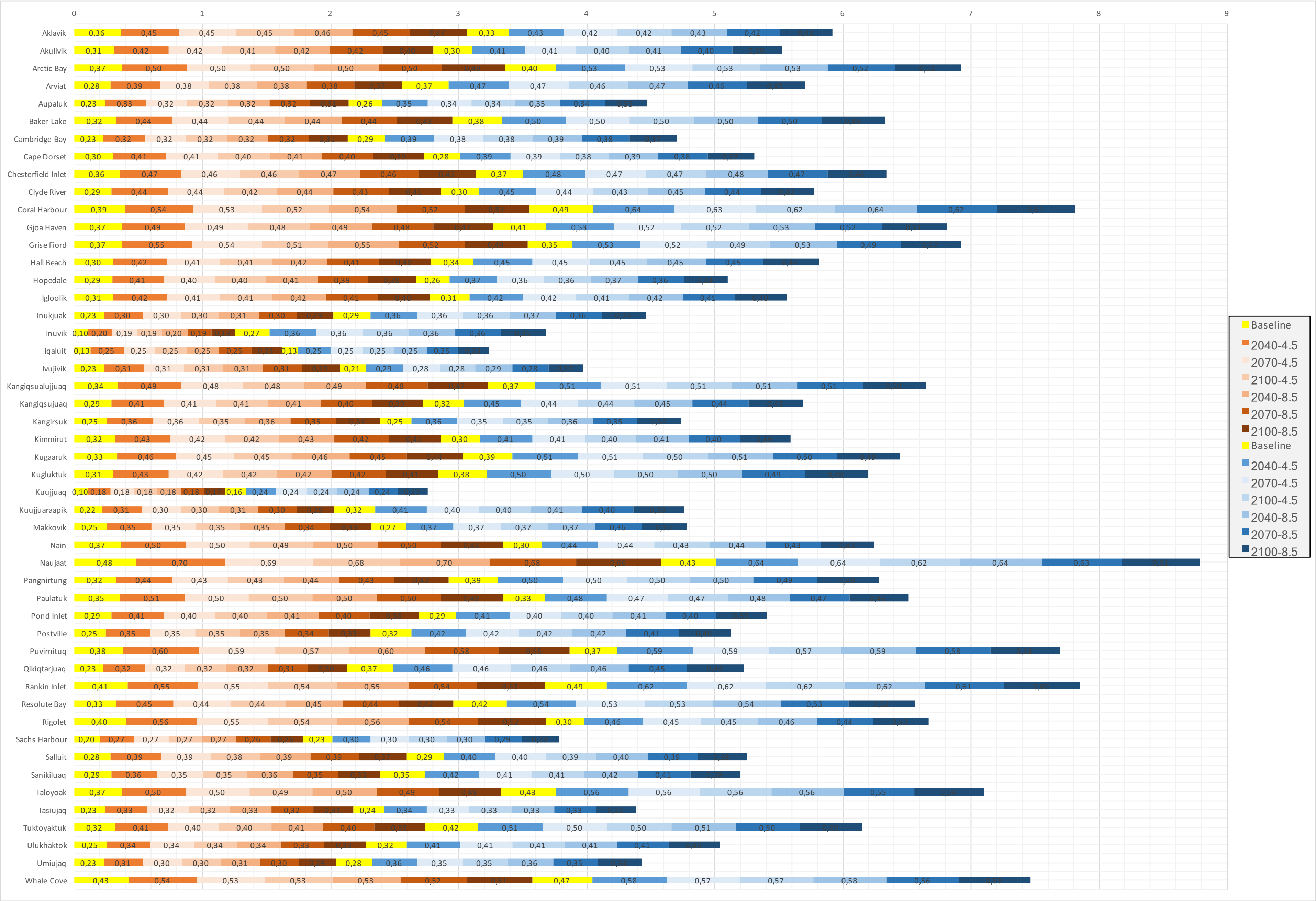
0
1
2
3
4
5
6
7
8
9
Aklavik
Akulivik
Arctic Bay
Arviat
Aupaluk
Baker Lake
Cambridge Bay
Cape Dorset
Chesterfield Inlet
Clyde River
Coral Harbour
Gjoa Haven
Grise Fiord
Hall Beach
Hopedale
Igloolik
Inukjuak
Inuvik
Iqaluit
Ivujivik
Kangiqsualujjuaq
Kangiqsujuaq
Kangirsuk
Kimmirut
Kugaaruk
Kugluktuk
Kuujjuaq
Kuujjuaraapik
Makkovik
Nain
Naujaat
Pangnirtung
Paulatuk
Pond Inlet
Postville
Puvirnituq
Qikiqtarjuaq
Rankin Inlet
Resolute Bay
Rigolet
Sachs Harbour
Salluit
Sanikiluaq
Taloyoak
Tasiujaq
Tuktoyaktuk
Ulukhaktok
Umiujaq
Whale Cove
Baseline
2040-4.5
2070-4.5
2100-4.5
2040-8.5
2070-8.5
2100-8.5
Baseline
2040-4.5
2070-4.5
2100-4.5
2040-8.5
2070-8.5
2100-8.5

Temperature Winter Model
Airport Vulnerability

RCP 4.5 | RCP 8.5

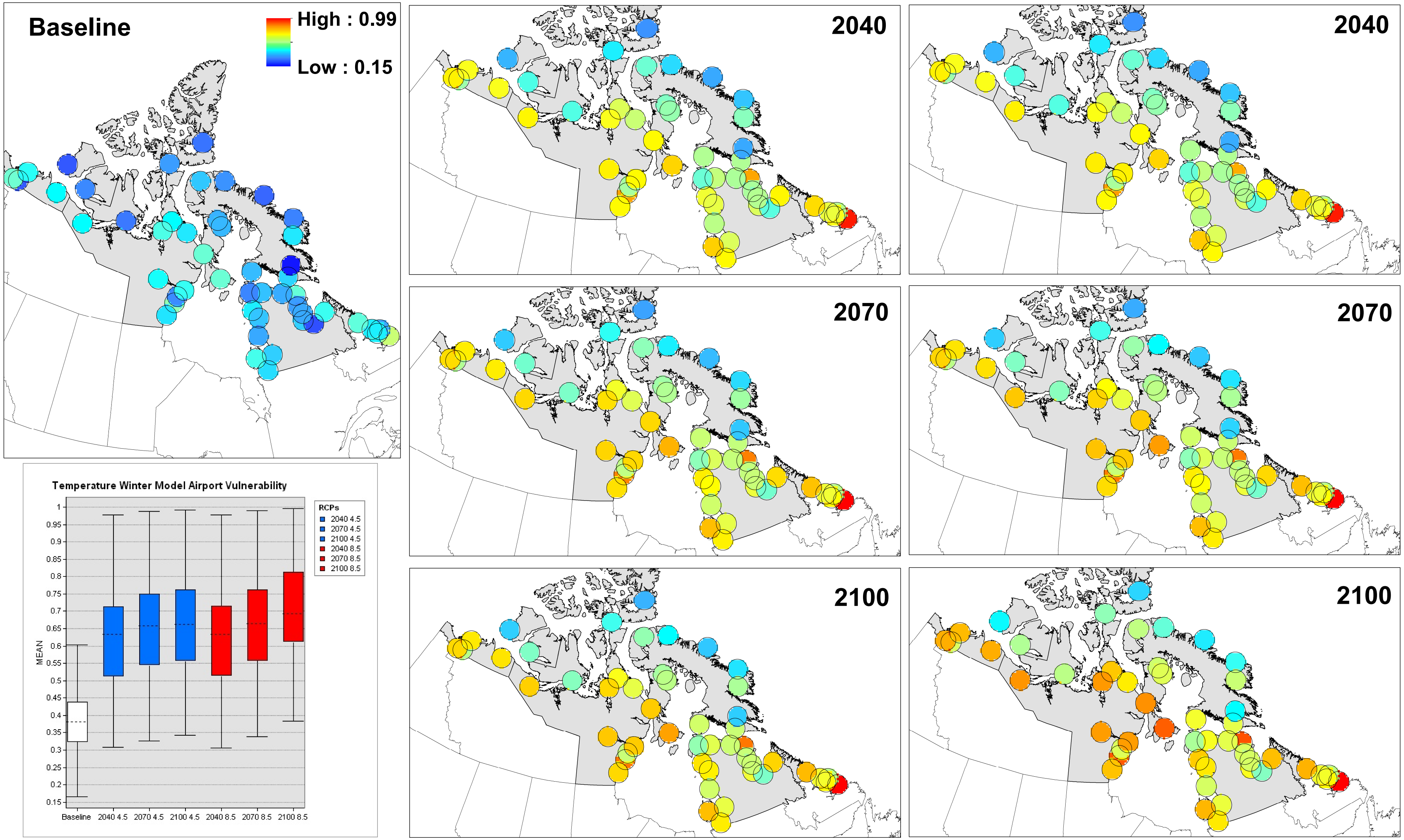

Temperature Winter Model
Marine Vulnerability

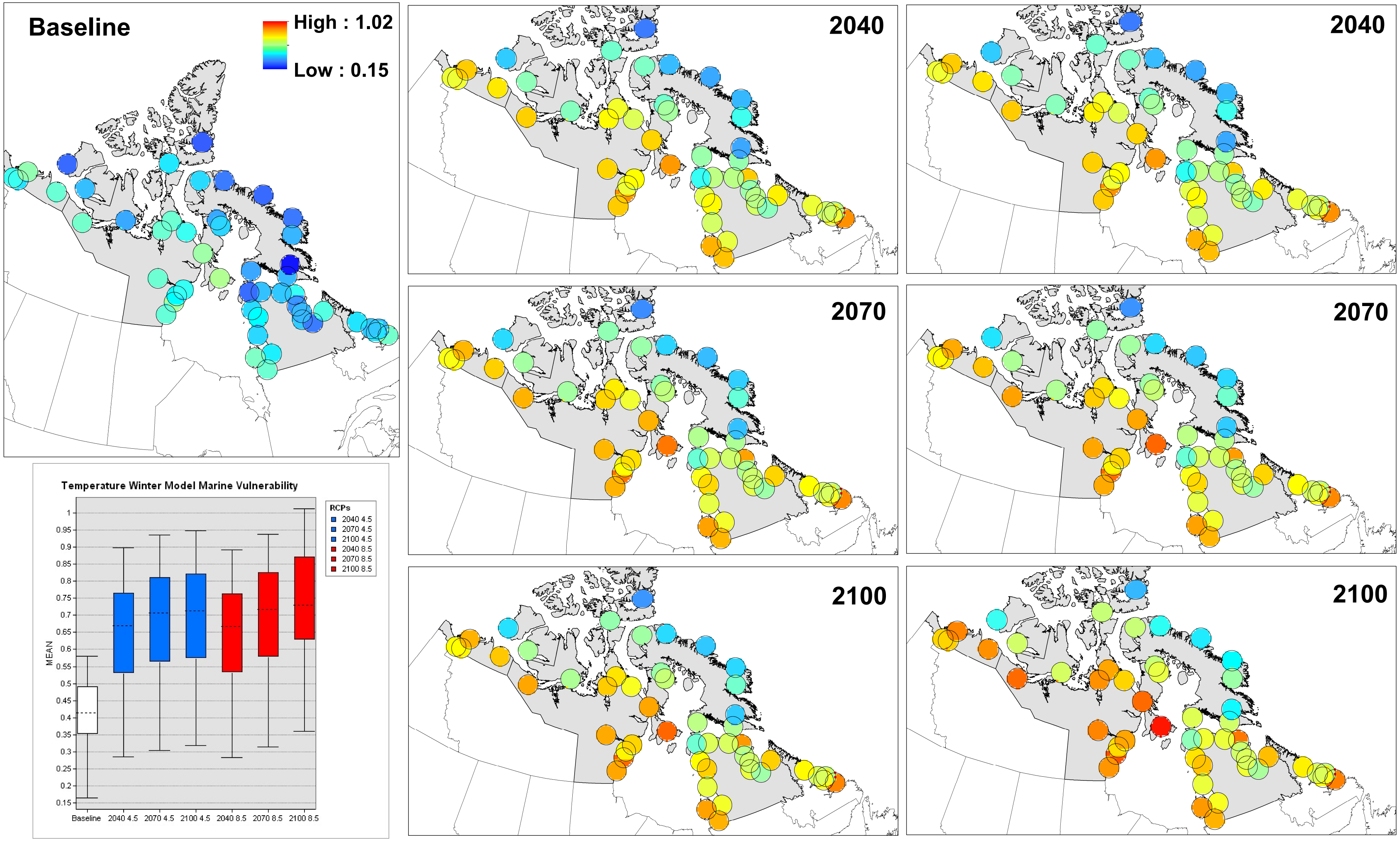

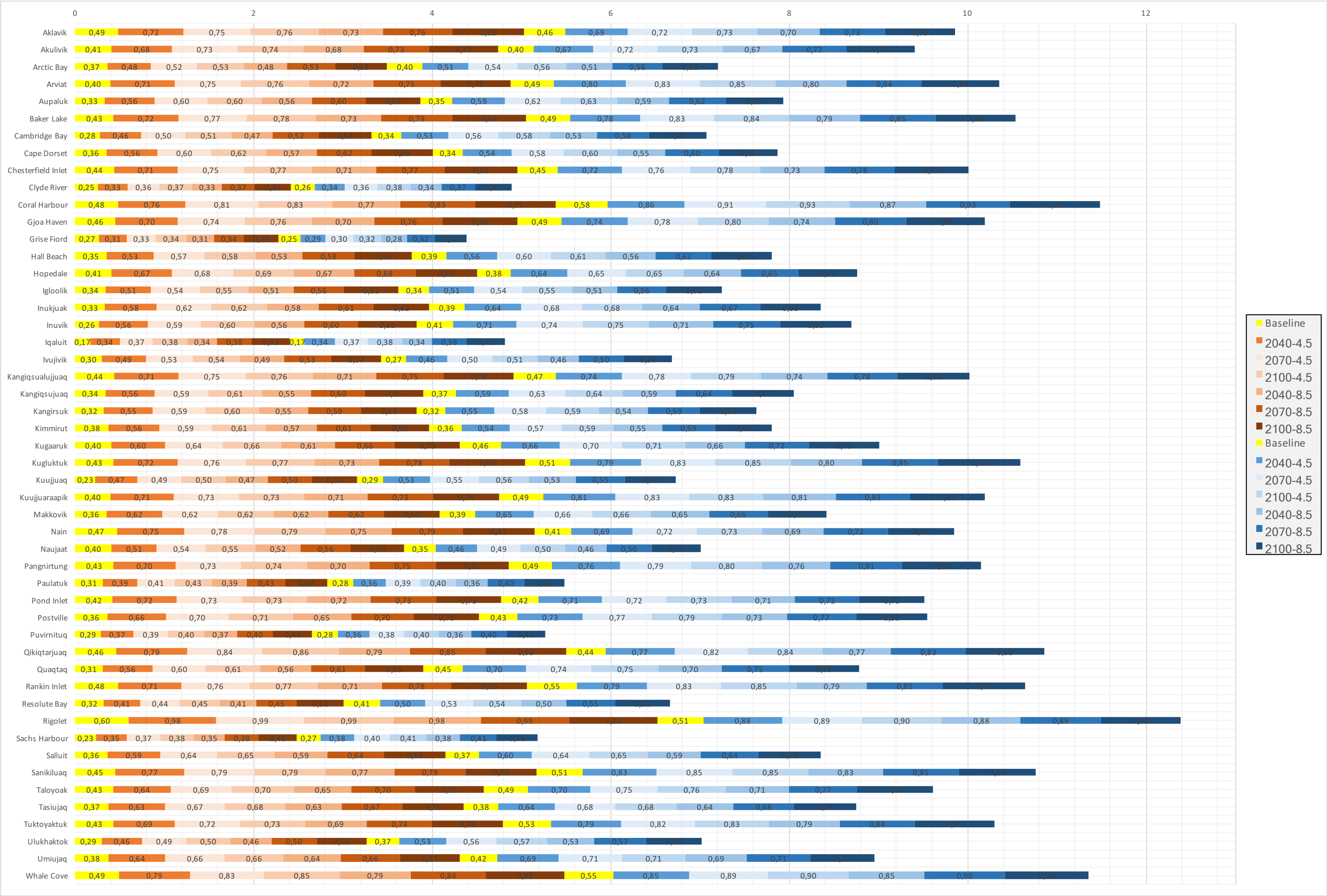
0
2
4
6
8
10
12
Aklavik
Akulivik
Arctic Bay
Arviat
Aupaluk
Baker Lake
Cambridge Bay
Cape Dorset
Chesterfield Inlet
Clyde River
Coral Harbour
Gjoa Haven
Grise Fiord
Hall Beach
Hopedale
Igloolik
Inukjuak
Inuvik
Iqaluit
Ivujivik
Kangiqsualujjuaq
Kangiqsujuaq
Kangirsuk
Kimmirut
Kugaaruk
Kugluktuk
Kuujjuaq
Kuujjuaraapik
Makkovik
Nain
Naujaat
Pangnirtung
Paulatuk
Pond Inlet
Postville
Puvirnituq
Qikiqtarjuaq
Quaqtaq
Rankin Inlet
Resolute Bay
Rigolet
Sachs Harbour
Salluit
Sanikiluaq
Taloyoak
Tasiujaq
Tuktoyaktuk
Ulukhaktok
Umiujaq
Whale Cove
Baseline
2040-4.5
2070-4.5
2100-4.5
2040-8.5
2070-8.5
2100-8.5
Baseline
2040-4.5
2070-4.5
2100-4.5
2040-8.5
2070-8.5
2100-8.5

Temperature Summer Model
Airport Vulnerability

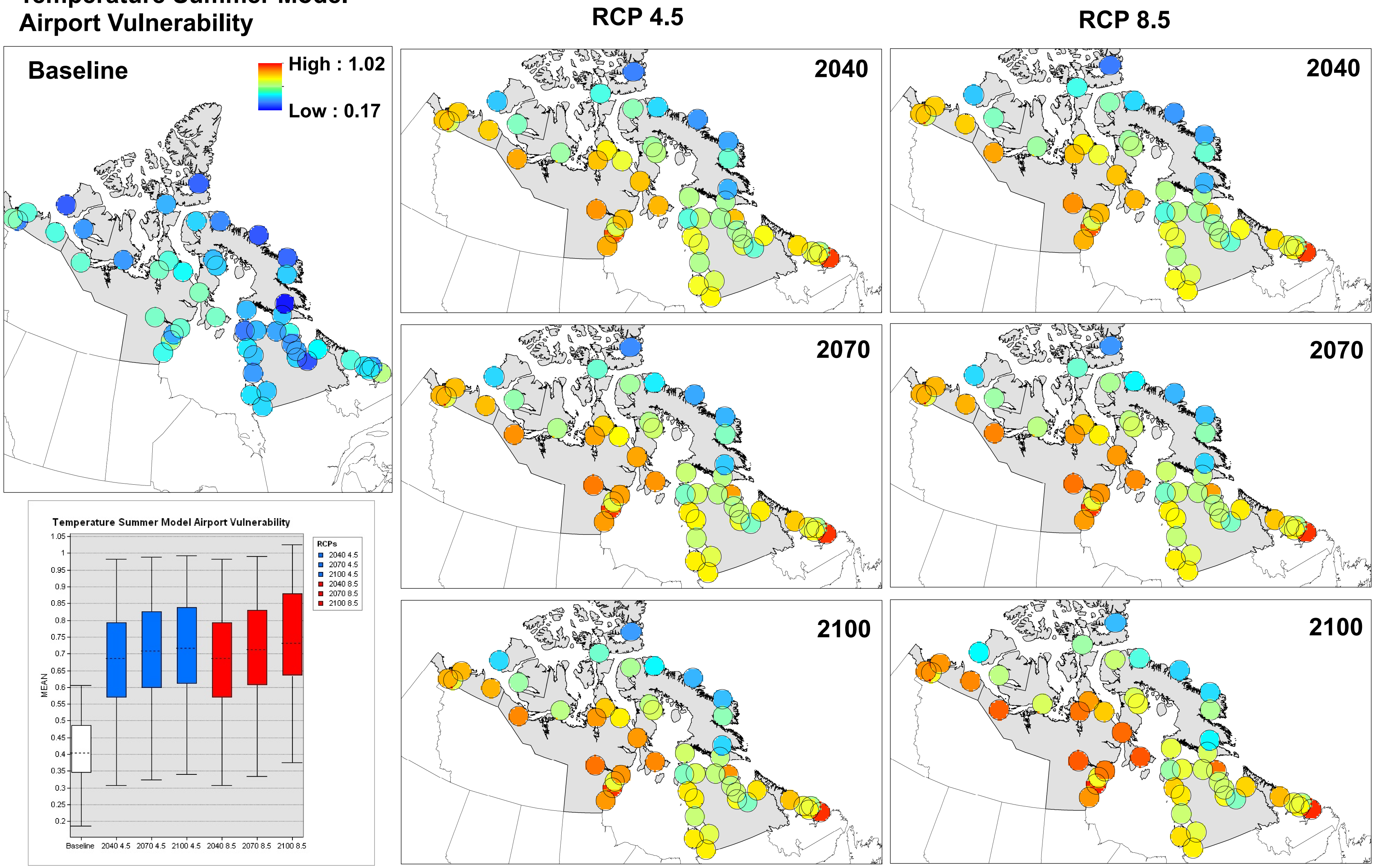

Temperature Summer Model
Marine Vulnerability

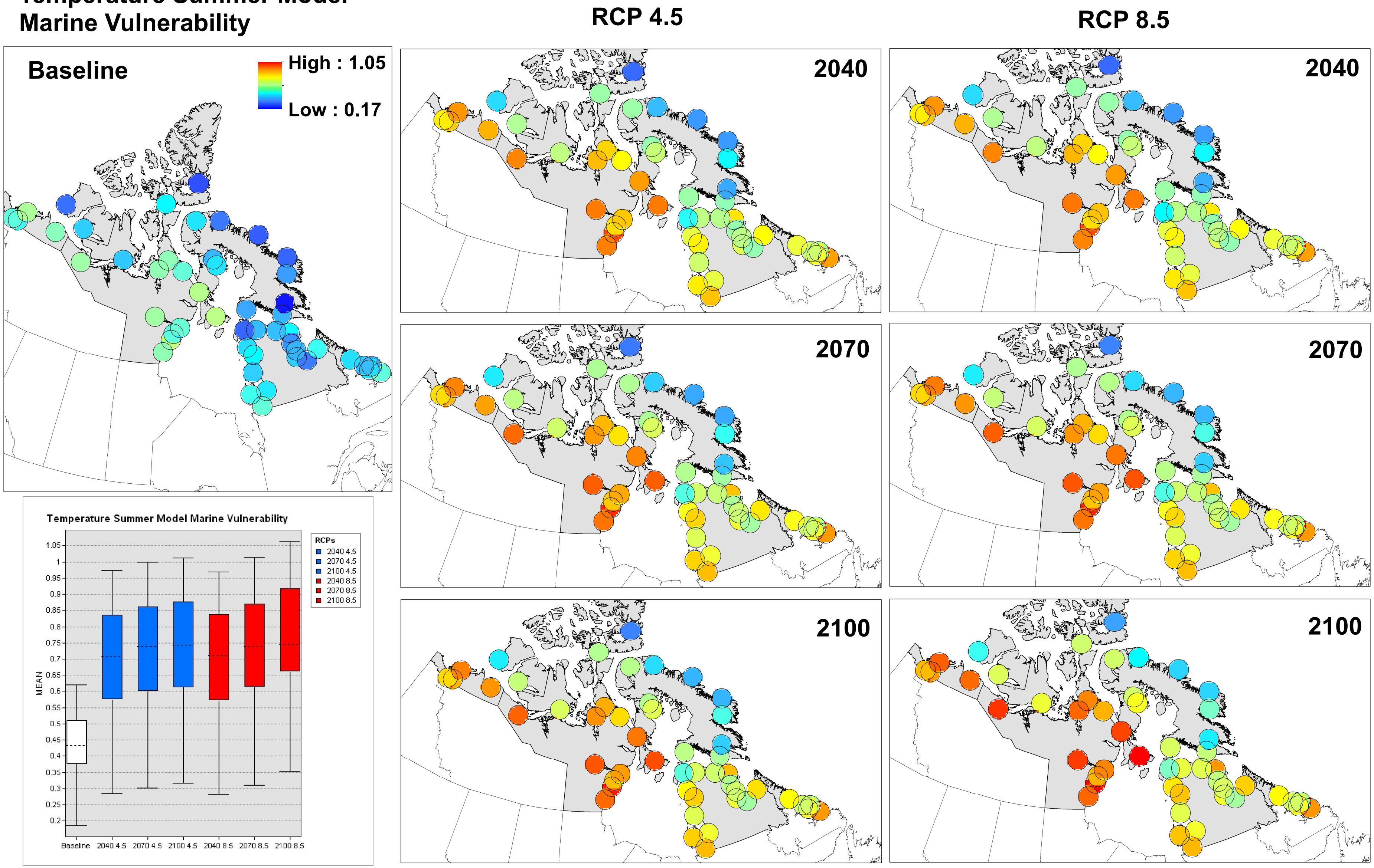

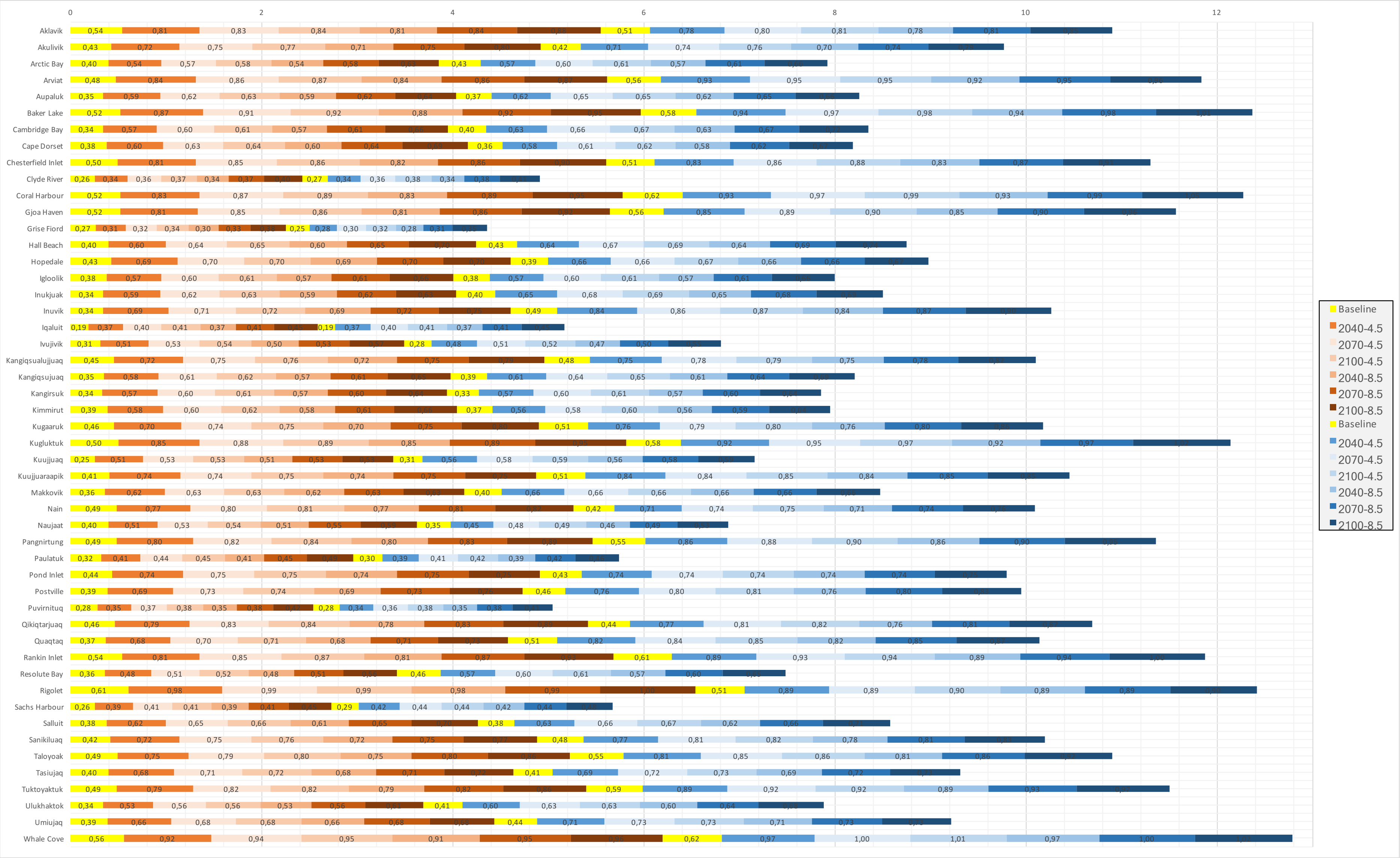
0
2
4
6
8
10
12
Aklavik
Akulivik
Arctic Bay
Arviat
Aupaluk
Baker Lake
Cambridge Bay
Cape Dorset
Chesterfield Inlet
Clyde River
Coral Harbour
Gjoa Haven
Grise Fiord
Hall Beach
Hopedale
Igloolik
Inukjuak
Inuvik
Iqaluit
Ivujivik
Kangiqsualujjuaq
Kangiqsujuaq
Kangirsuk
Kimmirut
Kugaaruk
Kugluktuk
Kuujjuaq
Kuujjuaraapik
Makkovik
Nain
Naujaat
Pangnirtung
Paulatuk
Pond Inlet
Postville
Puvirnituq
Qikiqtarjuaq
Quaqtaq
Rankin Inlet
Resolute Bay
Rigolet
Sachs Harbour
Salluit
Sanikiluaq
Taloyoak
Tasiujaq
Tuktoyaktuk
Ulukhaktok
Umiujaq
Whale Cove
Baseline
2040-4.5
2070-4.5
2100-4.5
2040-8.5
2070-8.5
2100-8.5
Baseline
2040-4.5
2070-4.5
2100-4.5
2040-8.5
2070-8.5
2100-8.5

Sea Level Model
Airport Vulnerability

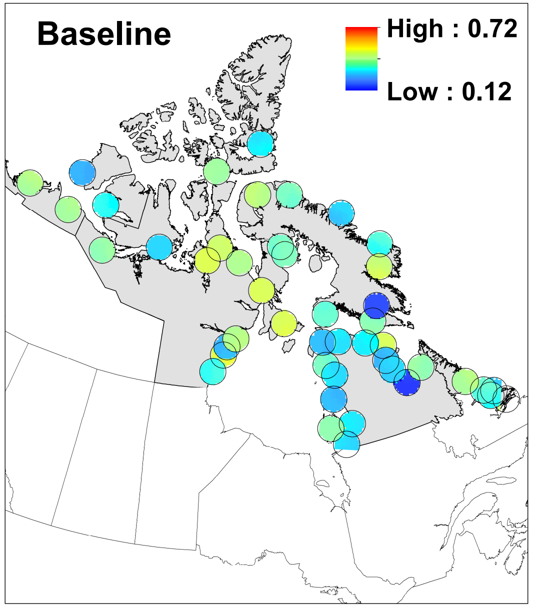

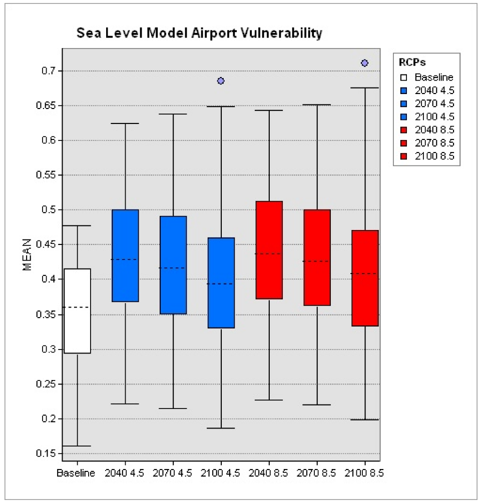

RCP 4.5 **RCP 8.5**

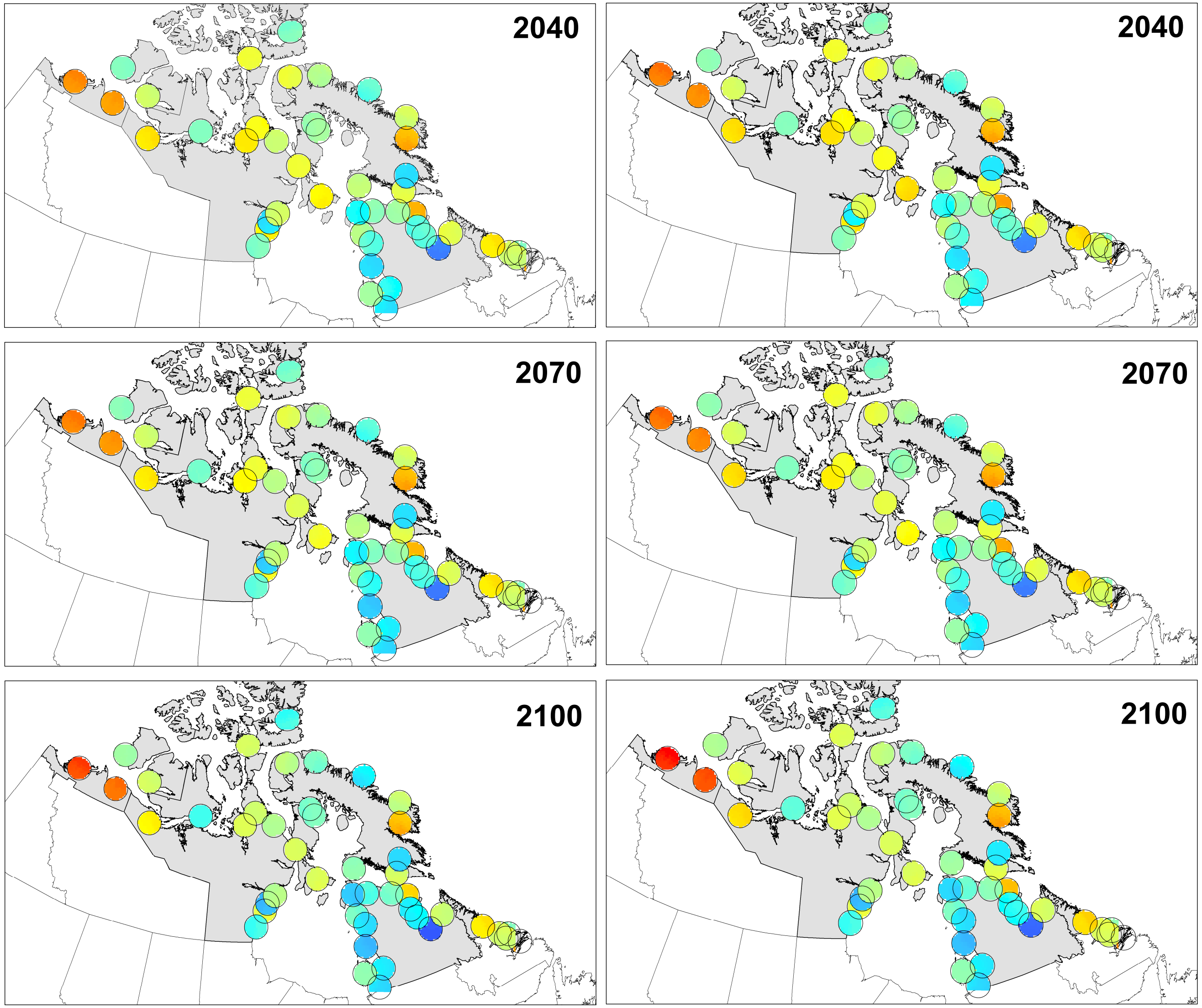

Sea Level Model
Marine Vulnerability

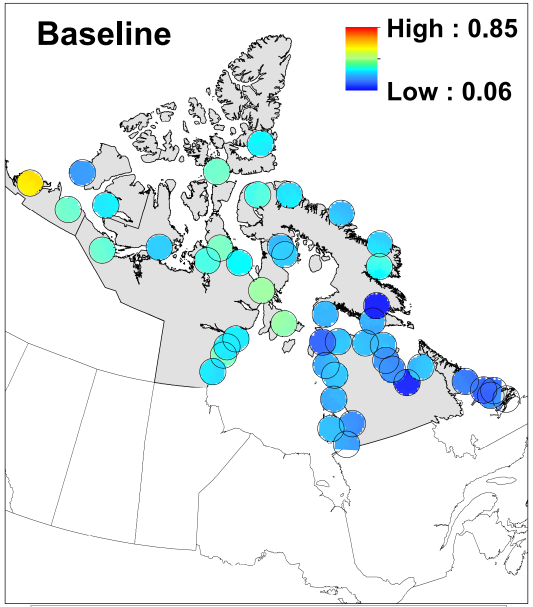

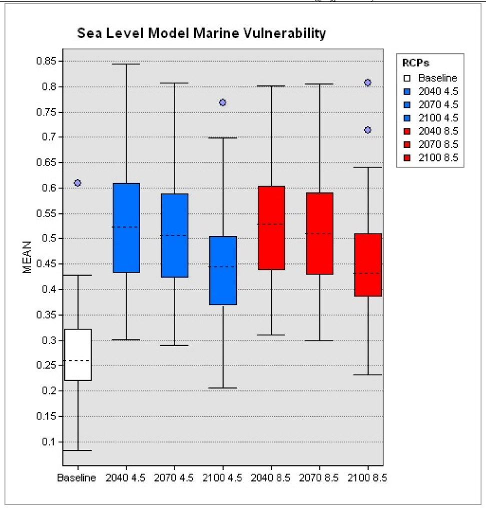

RCP 4.5

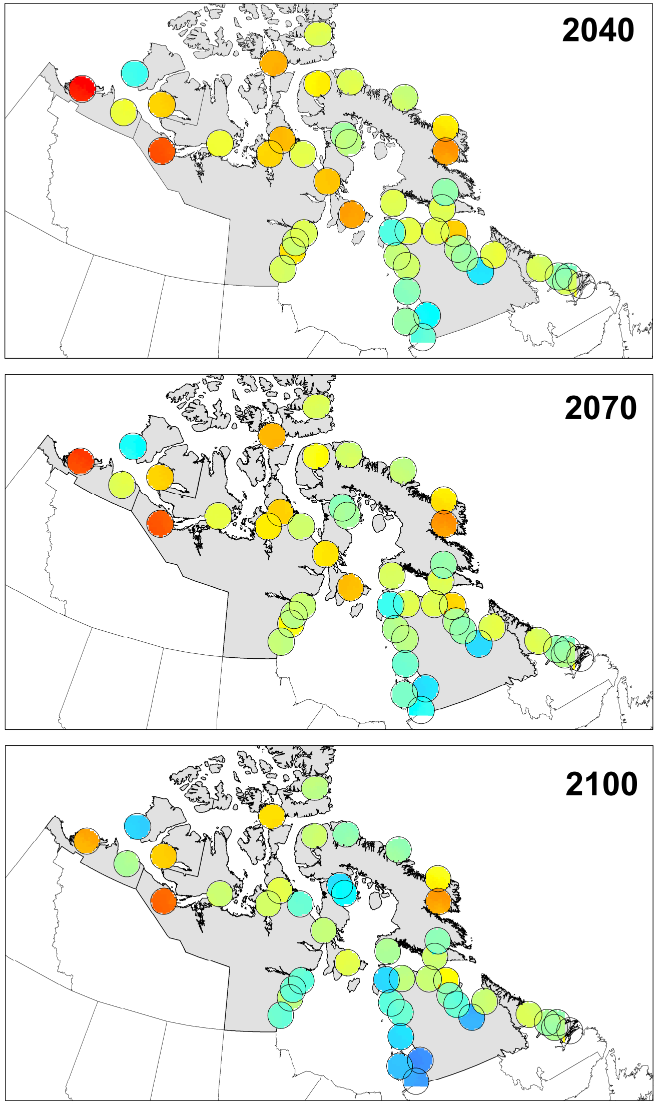

RCP 8.5

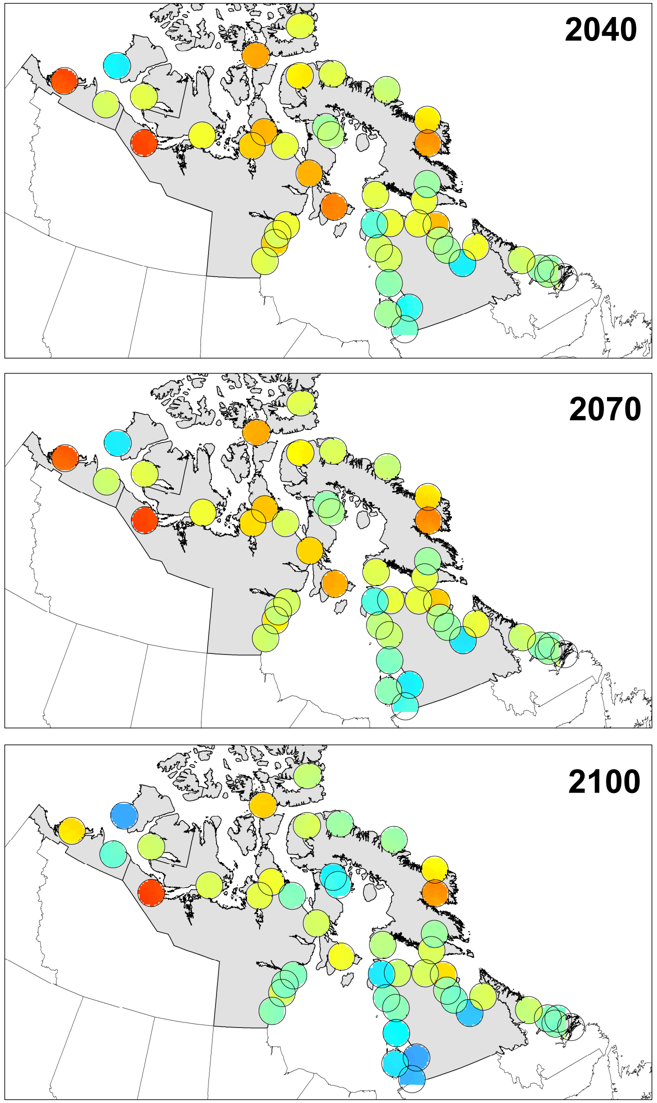

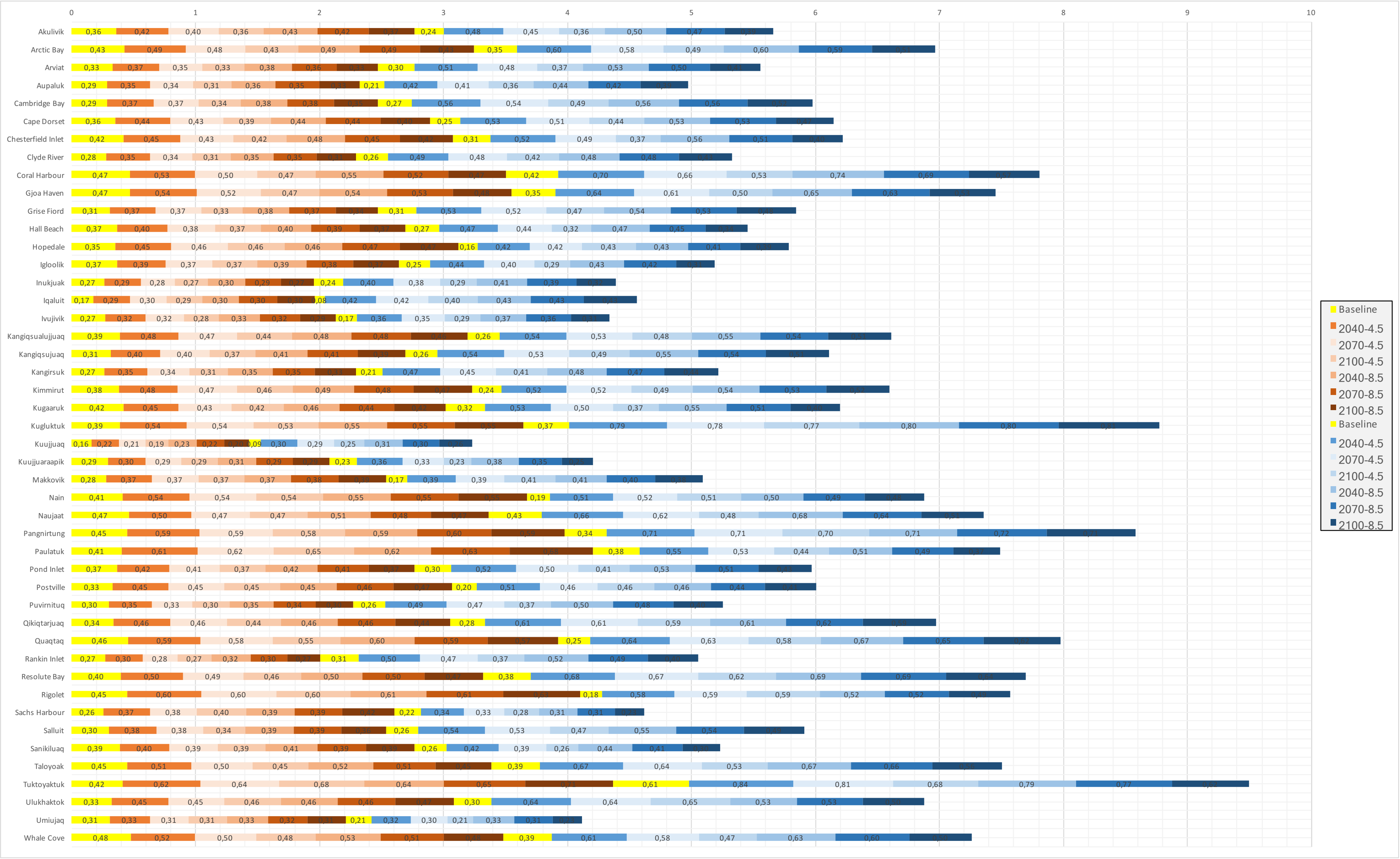
0
1
2
3
4
5
6
7
8
9
10
Akulivik
Arctic Bay
Arviat
Aupaluk
Cambridge Bay
Cape Dorset
Chesterfield Inlet
Clyde River
Coral Harbour
Gjoa Haven
Grise Fiord
Hall Beach
Hopedale
Igloolik
Inukjuak
Iqaluit
Ivujivik
Kangiqsualujjuaq
Kangiqsujuaq
Kangirsuk
Kimmirut
Kugaaruk
Kugluktuk
Kuujjuaq
Kuujjuaraapik
Makkovik
Nain
Naujaat
Pangnirtung
Paulatuk
Pond Inlet
Postville
Puvirnituq
Qikiqtarjuaq
Quaqtaq
Rankin Inlet
Resolute Bay
Rigolet
Sachs Harbour
Salluit
Sanikiluaq
Taloyoak
Tuktoyaktuk
Ulukhaktok
Umiujaq
Whale Cove
Baseline
2040-4.5
2070-4.5
2100-4.5
2040-8.5
2070-8.5
2100-8.5
Baseline
2040-4.5
2070-4.5
2100-4.5
2040-8.5
2070-8.5
2100-8.5

Supplementary Figure 4.

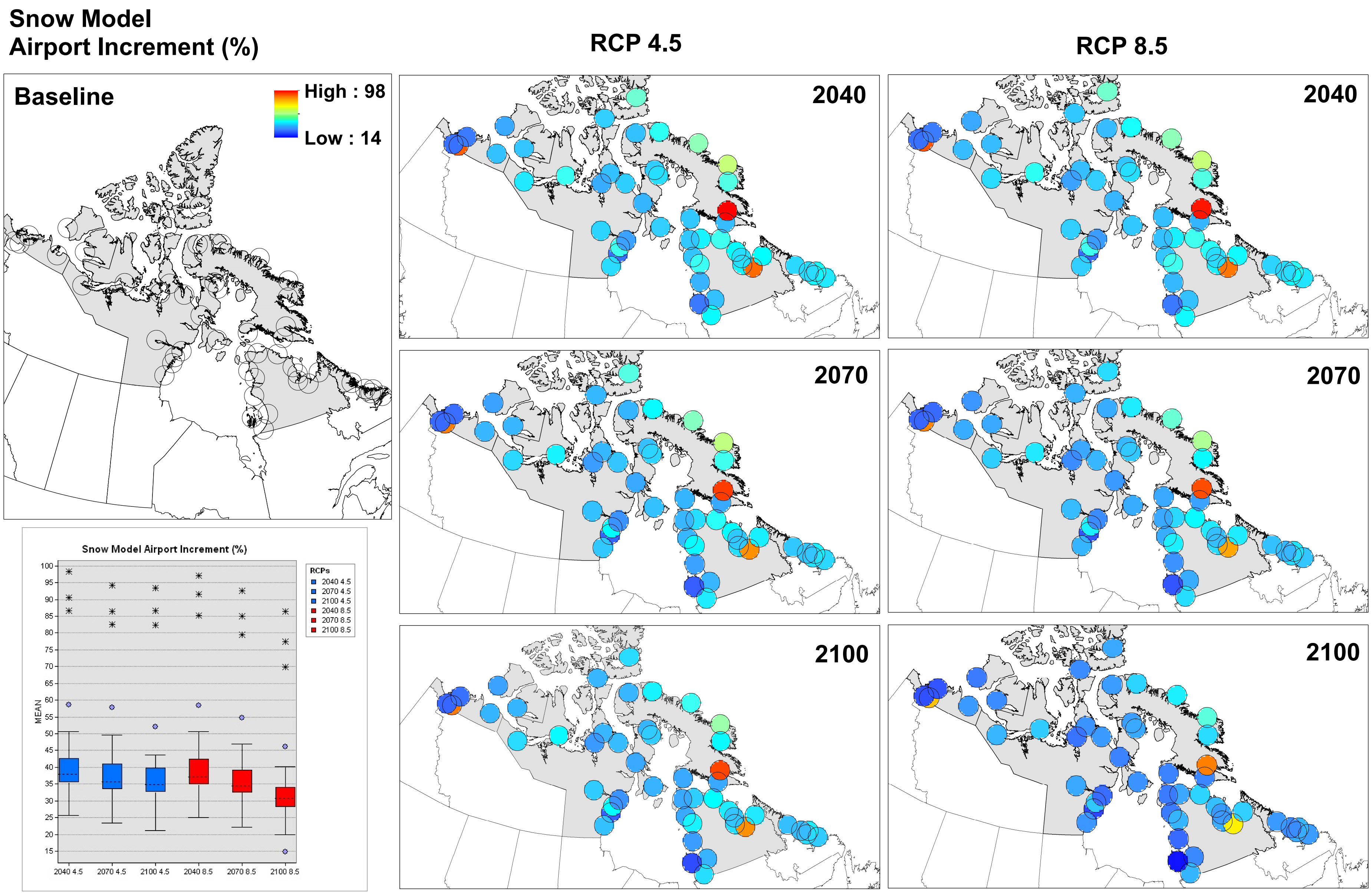

Snow Model
Marine Increment (%)

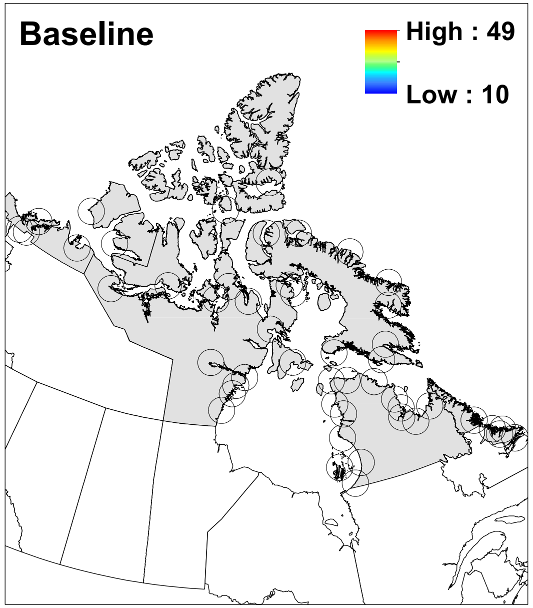

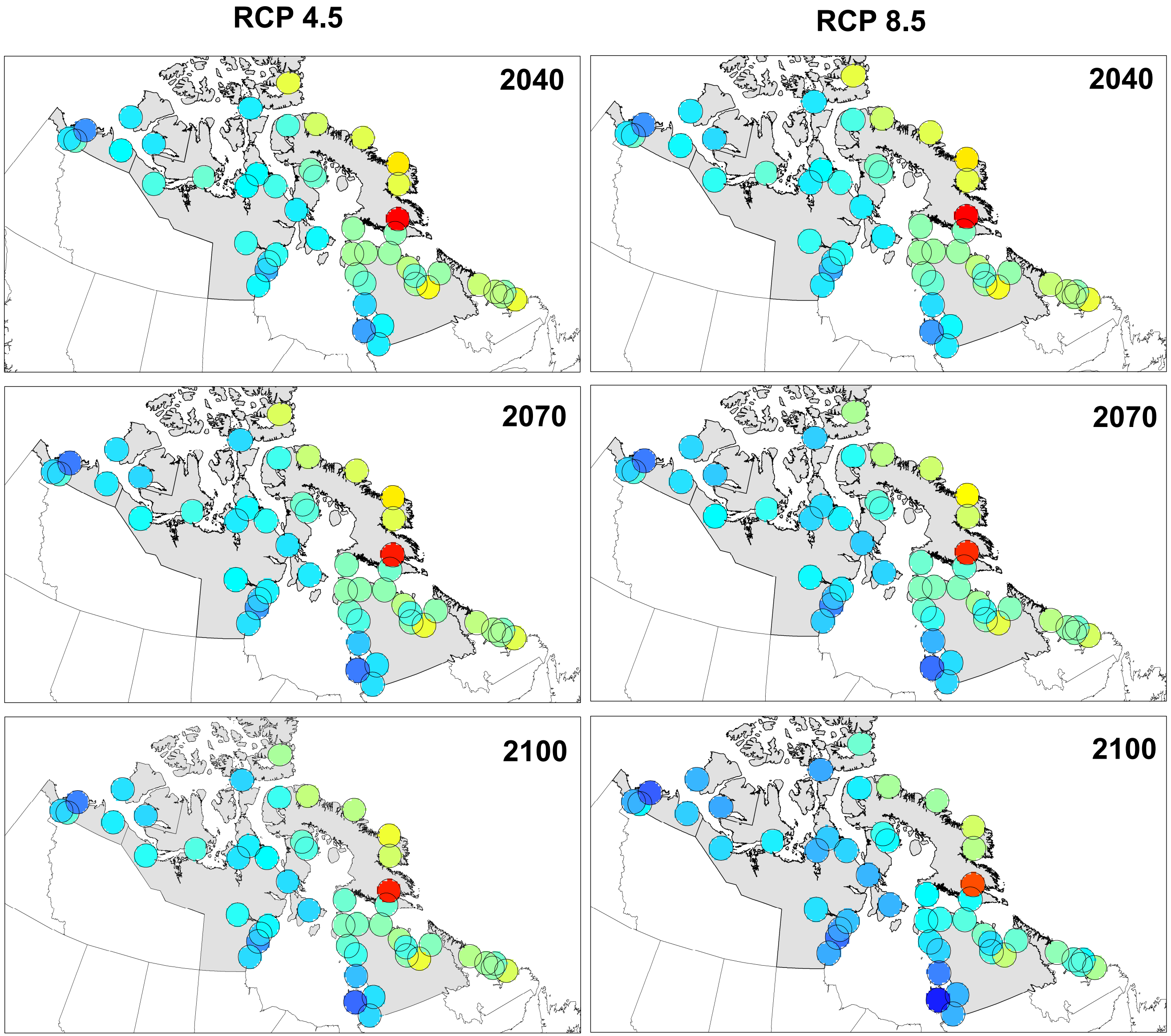

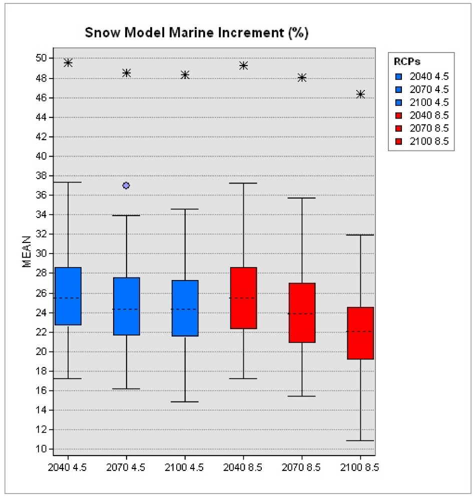

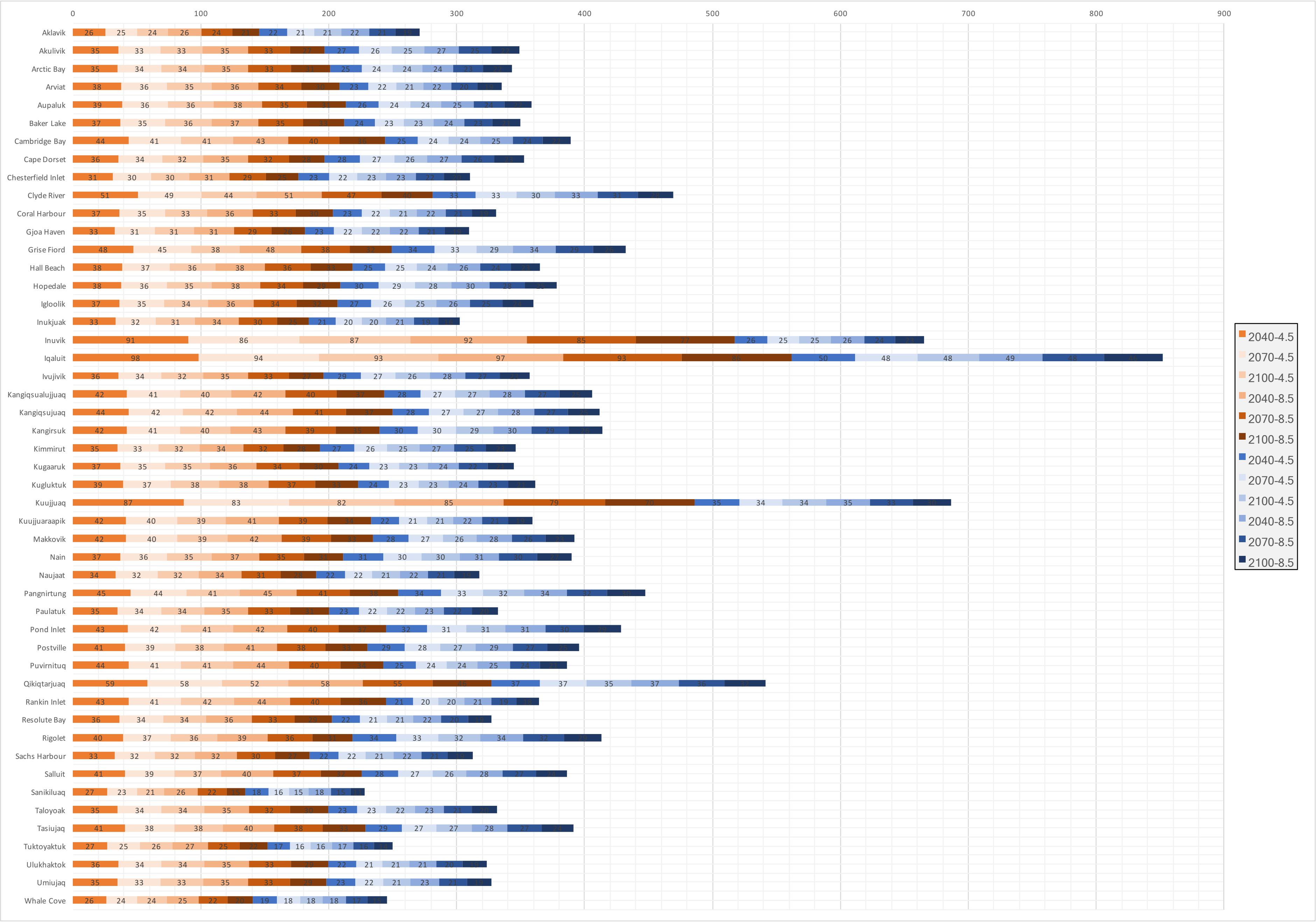
Aklavik
Akulivik
Arctic Bay
Arviat
Aupaluk
Baker Lake
Cambridge Bay
Cape Dorset
Chesterfield Inlet
Clyde River
Coral Harbour
Gjoa Haven
Grise Fiord
Hall Beach
Hopedale
Igloolik
Inukjuak
Inuvik
Iqaluit
Ivujivik
Kangiqsualujjuaq
Kangiqsujuaq
Kangirsuk
Kimmirut
Kugaaruk
Kugluktuk
Kuujjuaq
Kuujjuaraapik
Makkovik
Nain
Naujaat
Pangnirtung
Paulatuk
Pond Inlet
Postville
Puvirnituq
Qikiqtarjuaq
Rankin Inlet
Resolute Bay
Rigolet
Sachs Harbour
Salluit
Sanikiluaq
Taloyoak
Tasiujaq
Tuktoyaktuk
Ulukhaktok
Umiujaq
Whale Cove
2040-4.5
2070-4.5
2100-4.5
2040-8.5
2070-8.5
2100-8.5
2040-4.5
2070-4.5
2100-4.5
2040-8.5
2070-8.5
2100-8.5

Temperature Winter Model Airport Increment (%)

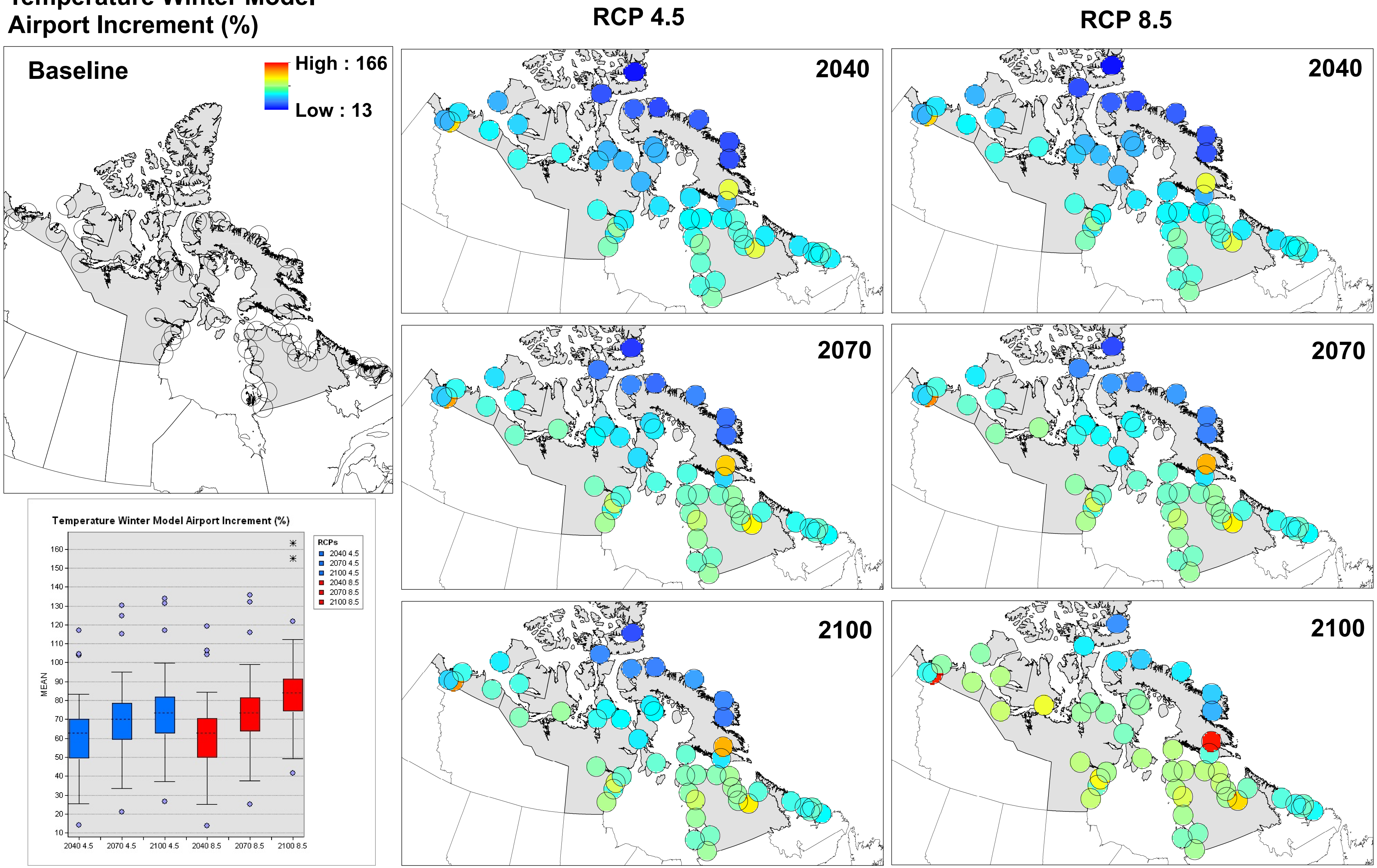

Temperature Winter Model Marine Increment (%)

RCP 4.5

RCP 8.5

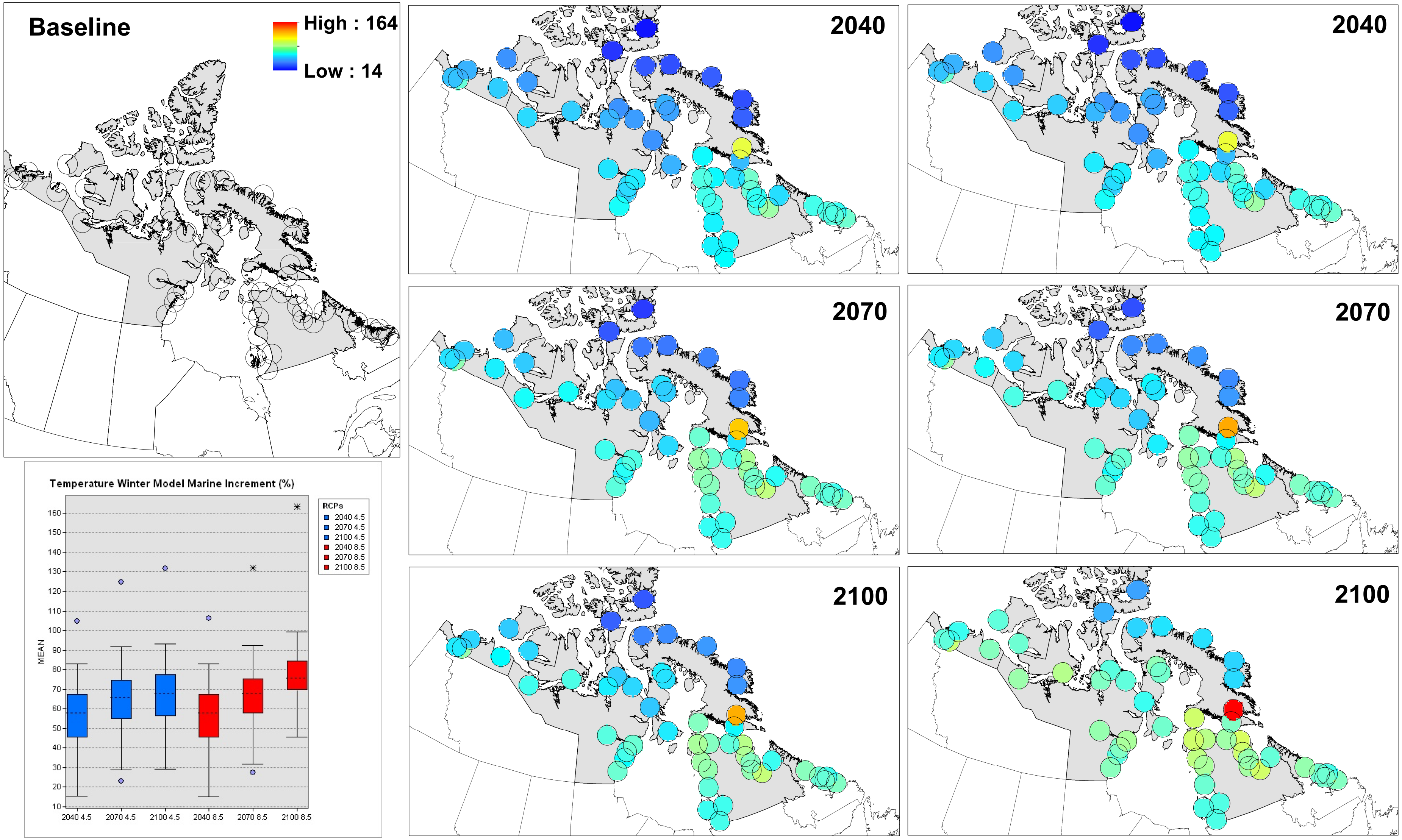

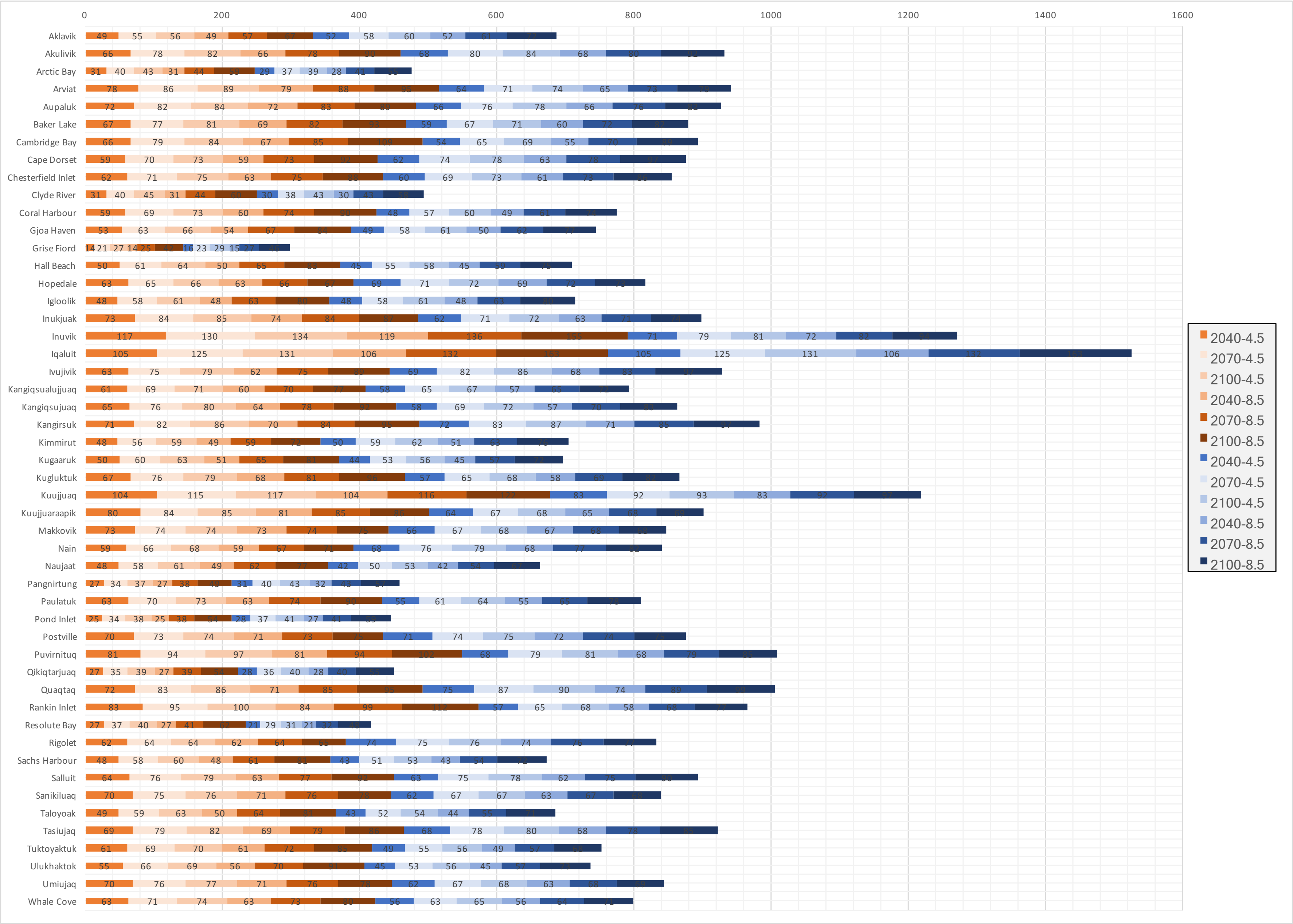
2040-4.5
2070-4.5
2100-4.5
2040-8.5
2070-8.5
2100-8.5
2040-4.5
2070-4.5
2100-4.5
2040-8.5
2070-8.5
2100-8.5

Temperature Summer Model Airport Increment (%)

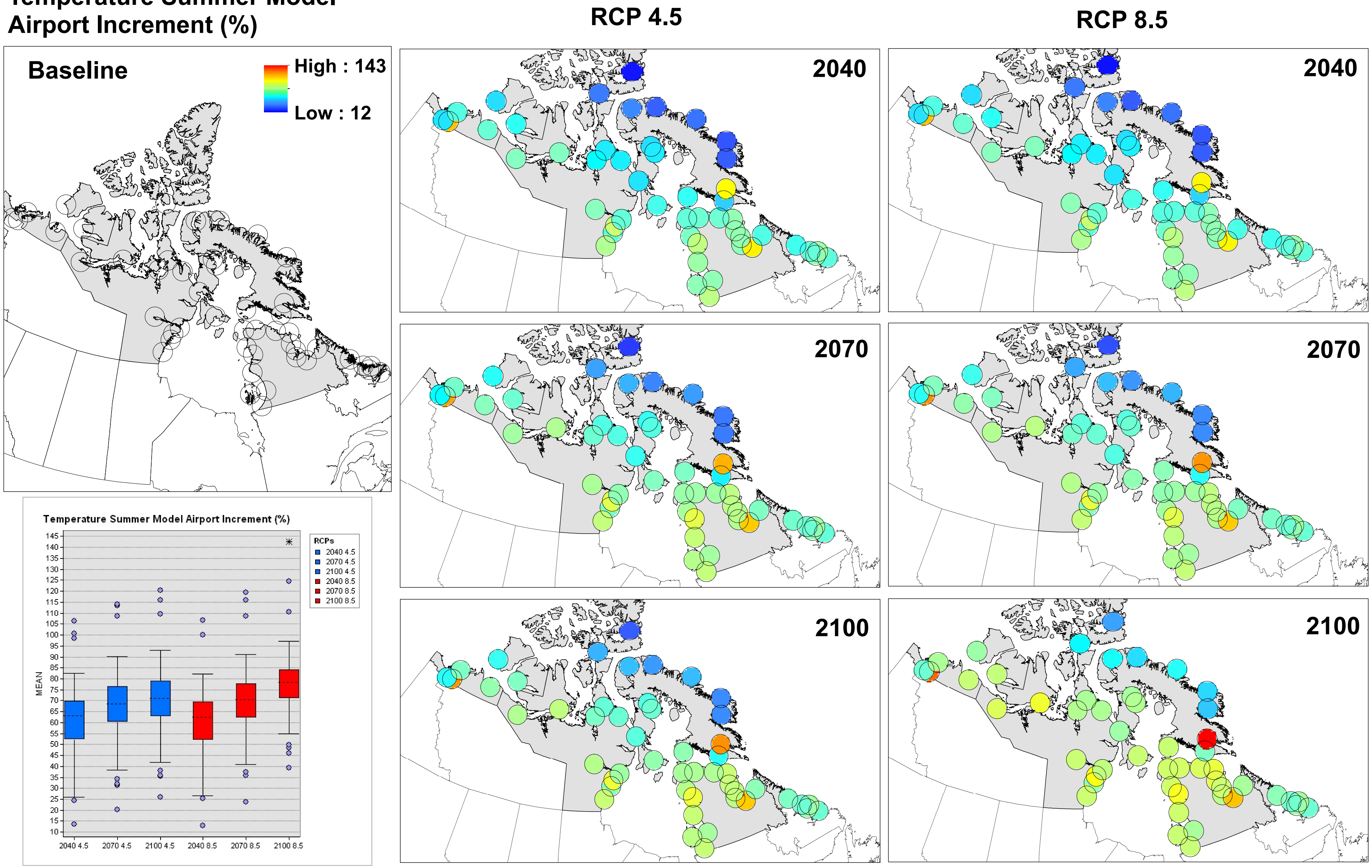

Temperature Summer Model Marine Increment (%)

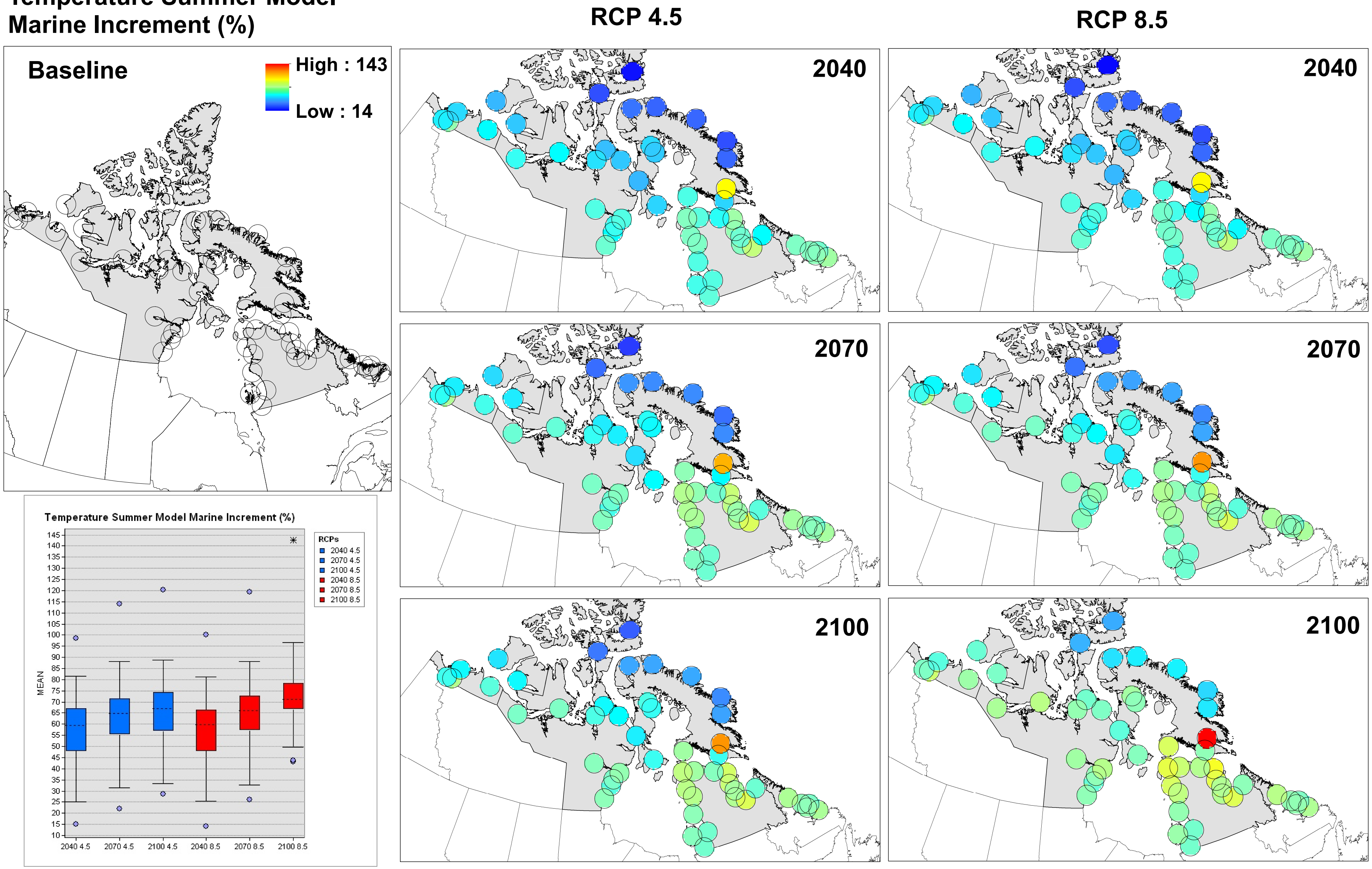

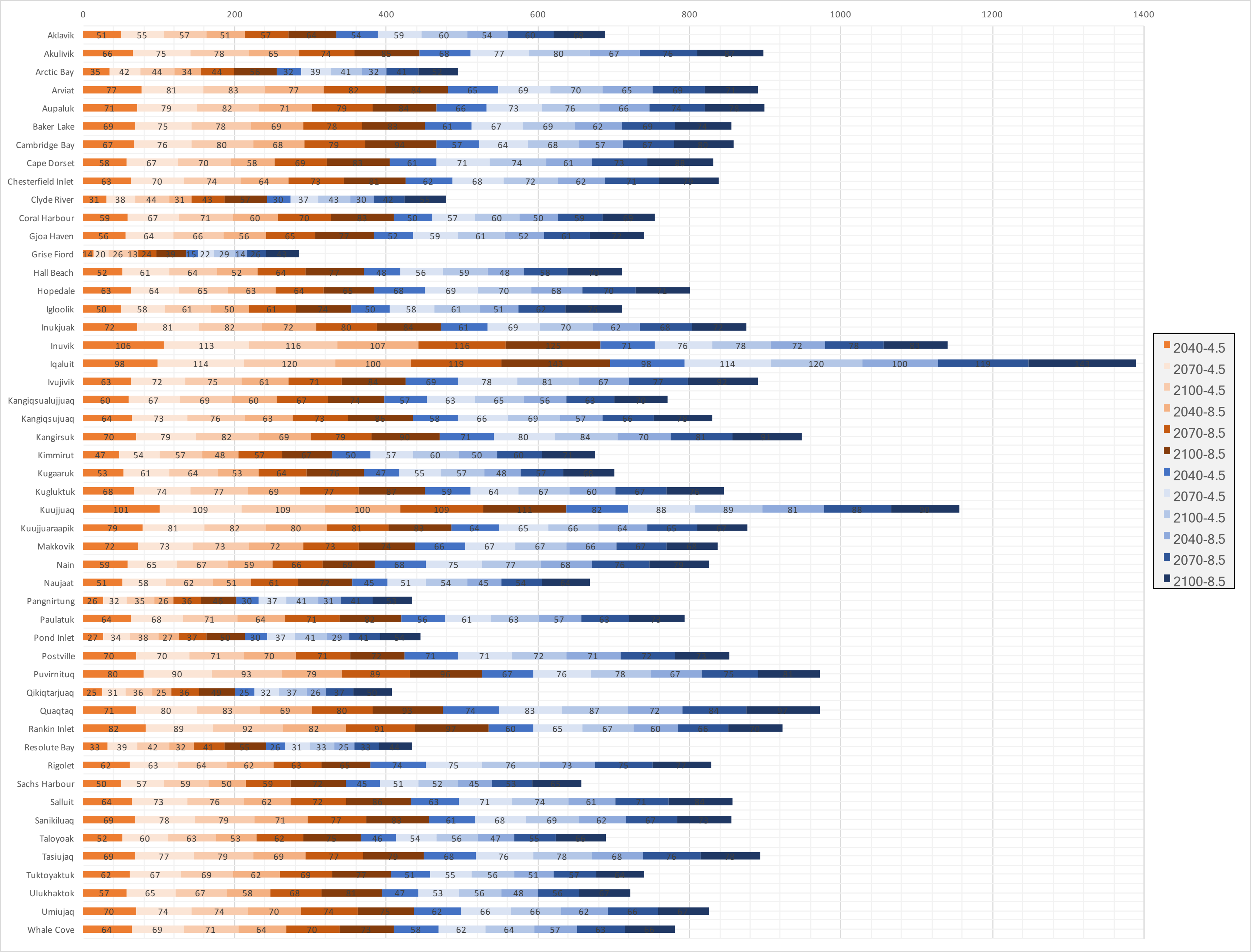
0
200
400
600
800
1000
1200
1400
Aklavik
Akulivik
Arctic Bay
Arviat
Aupaluk
Baker Lake
Cambridge Bay
Cape Dorset
Chesterfield Inlet
Clyde River
Coral Harbour
Gjoa Haven
Grise Fiord
Hall Beach
Hopedale
Igloolik
Inukjuak
Inuvik
Iqaluit
Ivujivik
Kangiqsualujjuaq
Kangiqsujuaq
Kangirsuk
Kimmirut
Kugaaruk
Kugluktuk
Kuujjuaq
Kuujjuaraapik
Makkovik
Nain
Naujaat
Pangnirtung
Paulatuk
Pond Inlet
Postville
Puvirnituq
Qikiqtarjuaq
Quaqtaq
Rankin Inlet
Resolute Bay
Rigolet
Sachs Harbour
Salluit
Sanikiluaq
Taloyoak
Tasiujaq
Tuktoyaktuk
Ulukhaktok
Umiujaq
Whale Cove
2040-4.5
2070-4.5
2100-4.5
2040-8.5
2070-8.5
2100-8.5
2040-4.5
2070-4.5
2100-4.5
2040-8.5
2070-8.5
2100-8.5

Sea Level Model
Airport Increment (%)

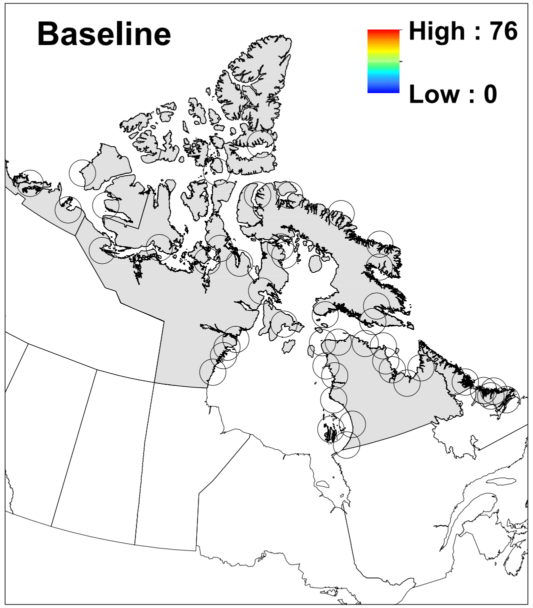

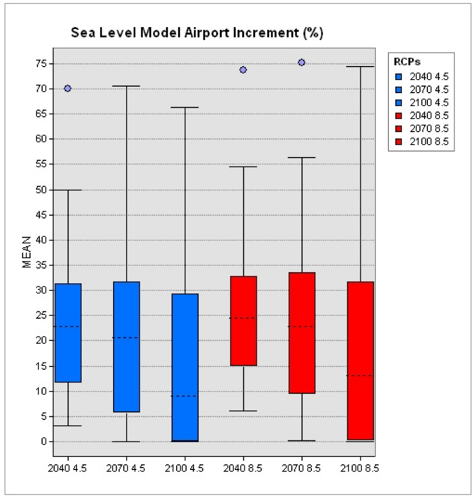

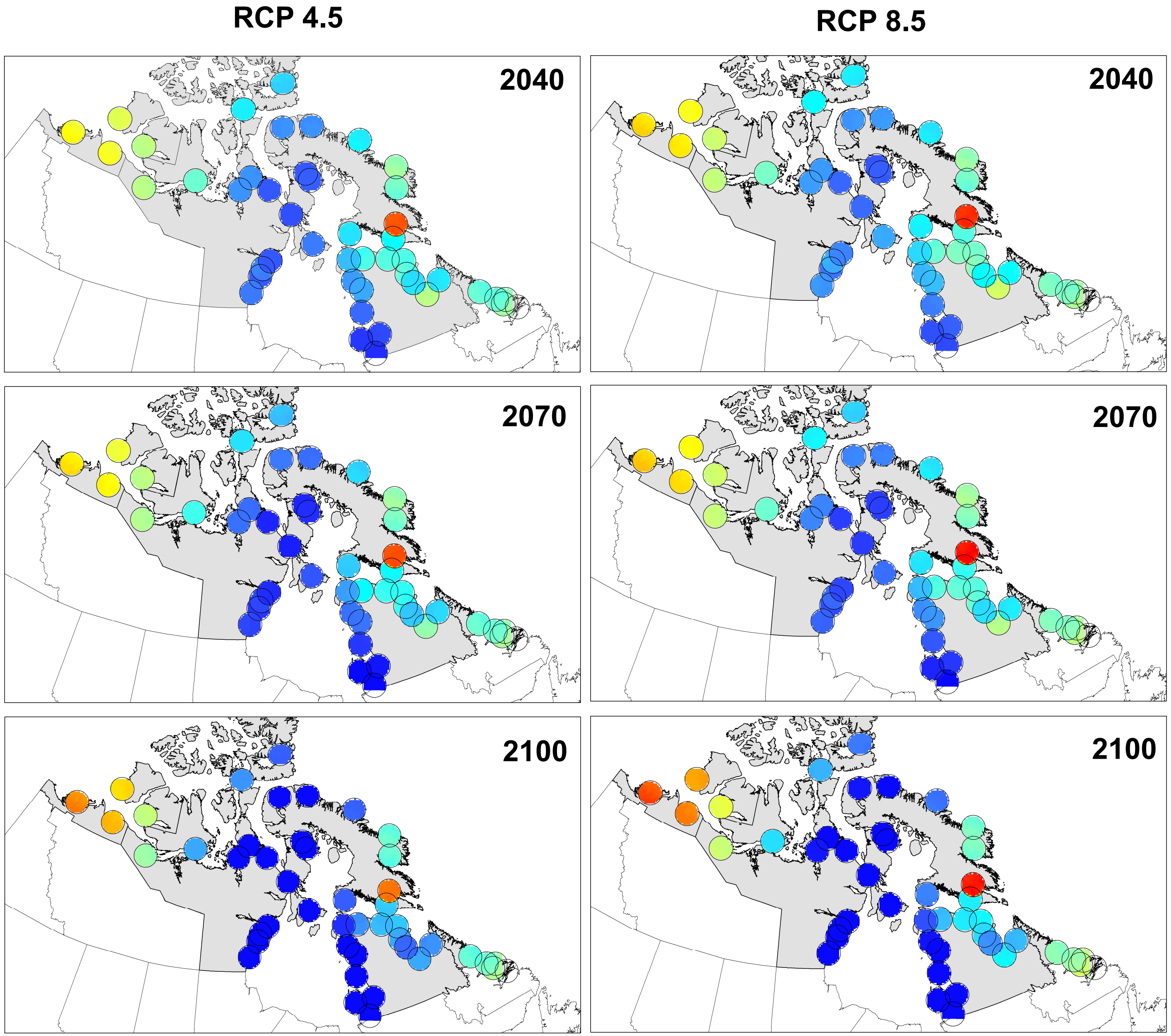

Sea Level Model
Marine Increment (%)

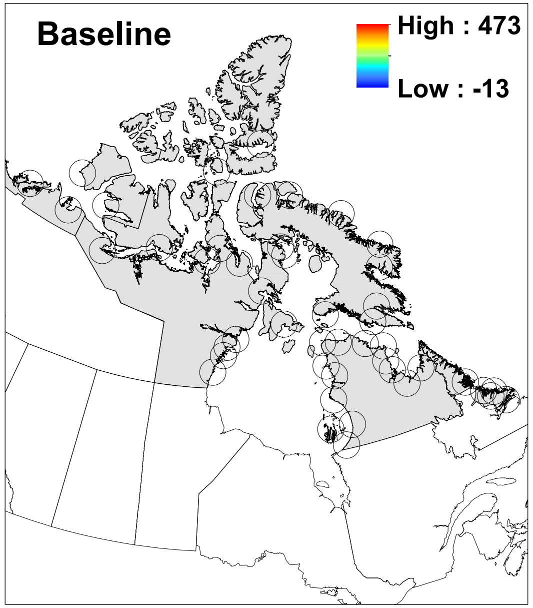

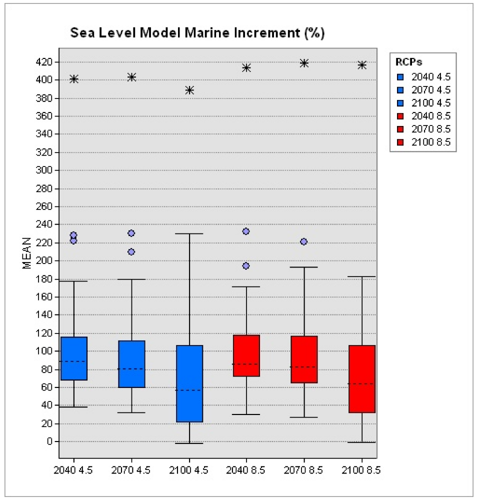

RCP 4.5

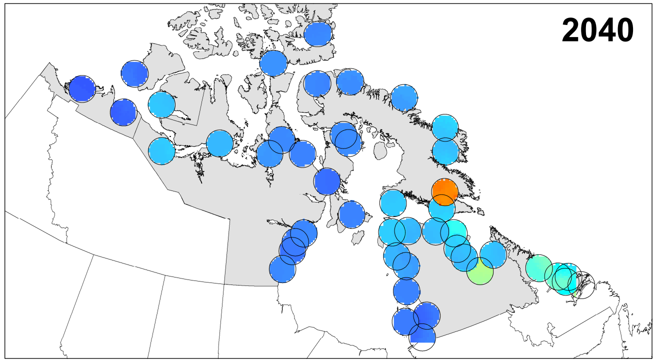

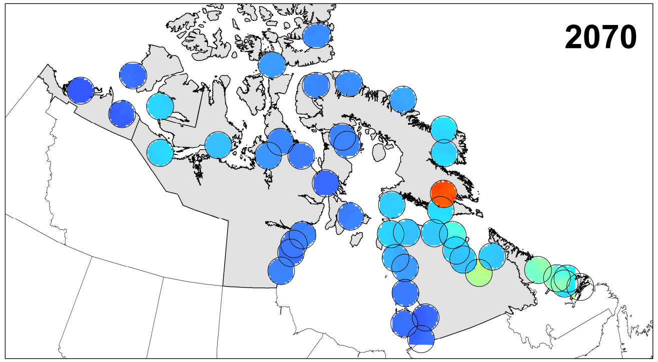

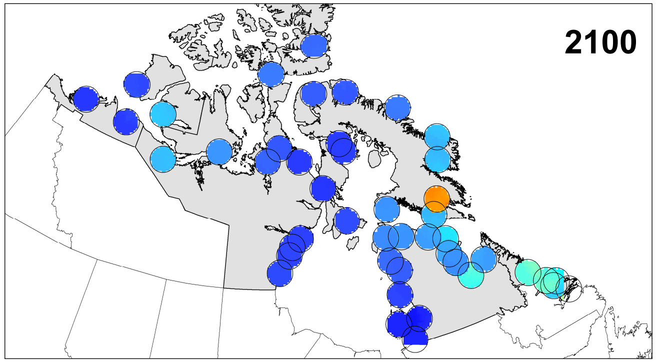

RCP 8.5

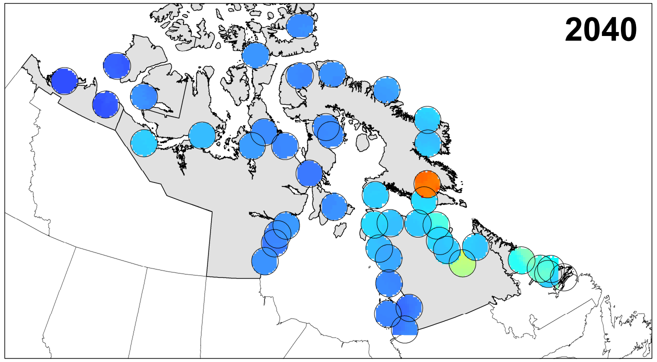

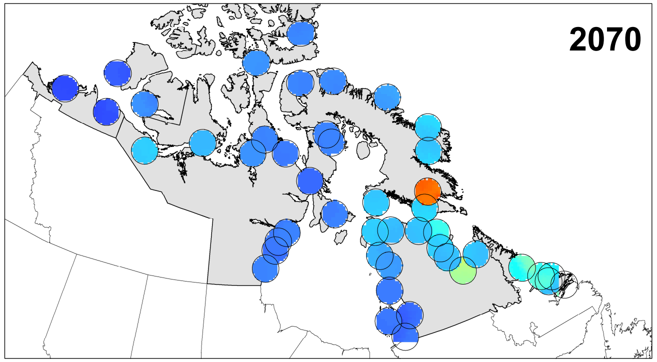

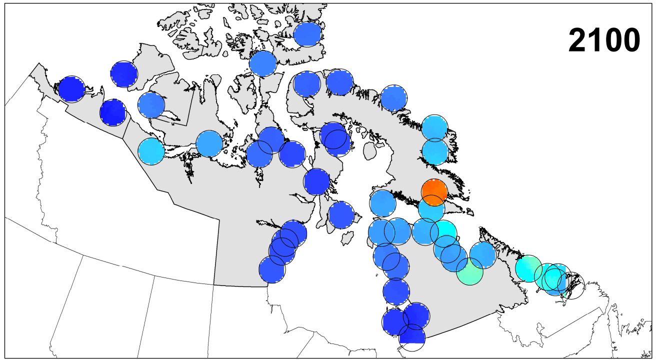

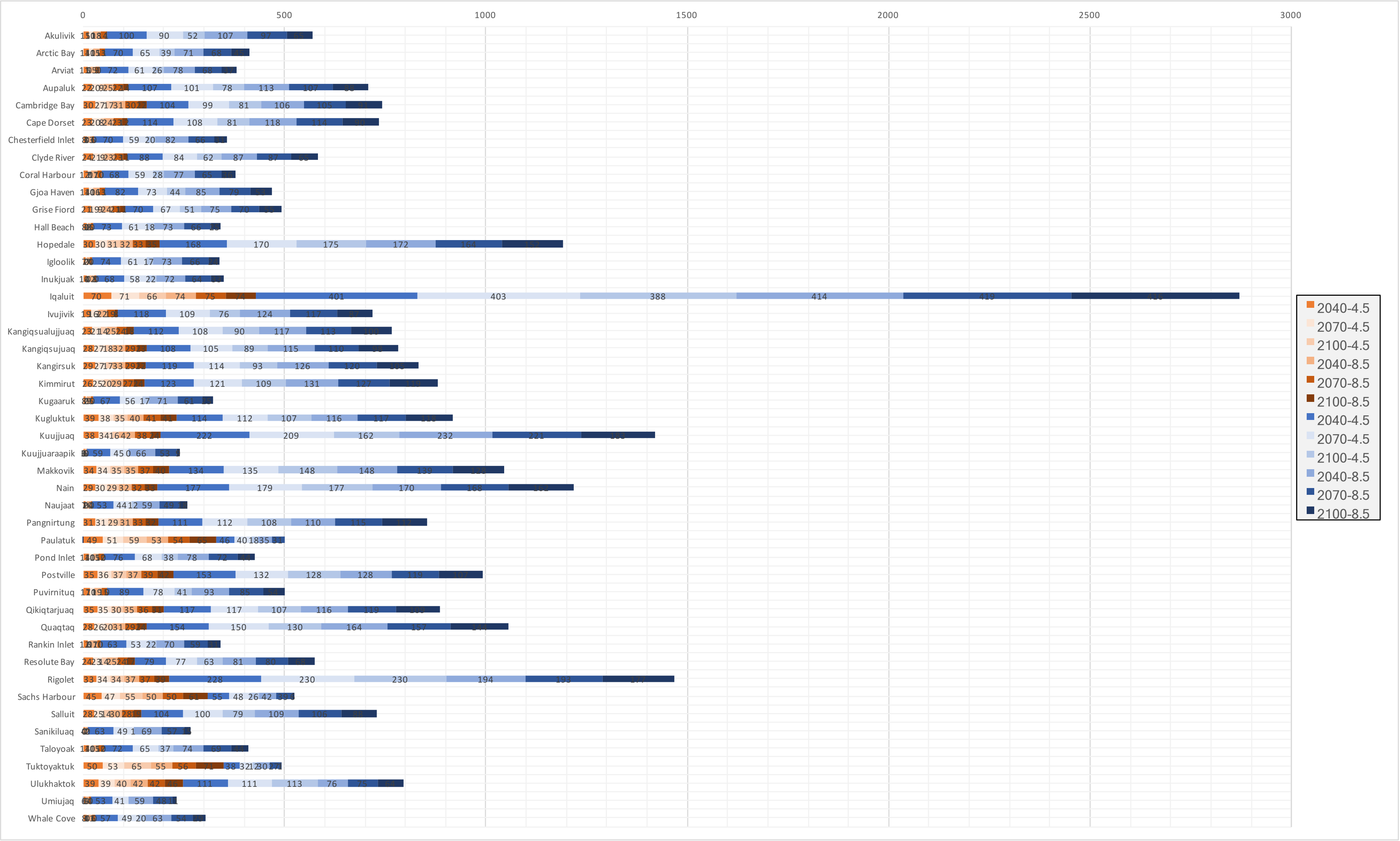
0
500
1000
1500
2000
2500
3000
Akulivik
Arctic Bay
Arviat
Aupaluk
Cambridge Bay
Cape Dorset
Chesterfield Inlet
Clyde River
Coral Harbour
Gjoa Haven
Grise Fiord
Hall Beach
Hopedale
Igloolik
Inukjuak
Iqaluit
Ivujivik
Kangiqsualujjuaq
Kangiqsujuaq
Kangirsuk
Kimmirut
Kugaaruk
Kugluktuk
Kuujjuaq
Kuujjuaraapik
Makkovik
Nain
Naujaat
Pangnirtung
Paulatuk
Pond Inlet
Postville
Puvirnituq
Qikiqtarjuaq
Quaqtaq
Rankin Inlet
Resolute Bay
Rigolet
Sachs Harbour
Salluit
Sanikiluaq
Taloyoak
Tuktoyaktuk
Ulukhaktok
Umiujaq
Whale Cove
2040-4.5
2070-4.5
2100-4.5
2040-8.5
2070-8.5
2100-8.5
2040-4.5
2070-4.5
2100-4.5
2040-8.5
2070-8.5
2100-8.5

Supplementary Figure 5. The network figures illustrate exposure (a), sensitivity (b), and adaptive capacity (c) results for the transportation and infrastructure network analysis. Nodes size is proportional to the total number of network connections (Freeman's degree centrality, C_D). Blue lines are positive relationships (an increase/decrease in x variable causes the same change in y). Black lines are negative relationships (an increase/decrease in x variable causes the opposite change in y). Grey dashed lines are variable-unknown-undetermined relationships (outcome is ambiguous positive-negative).

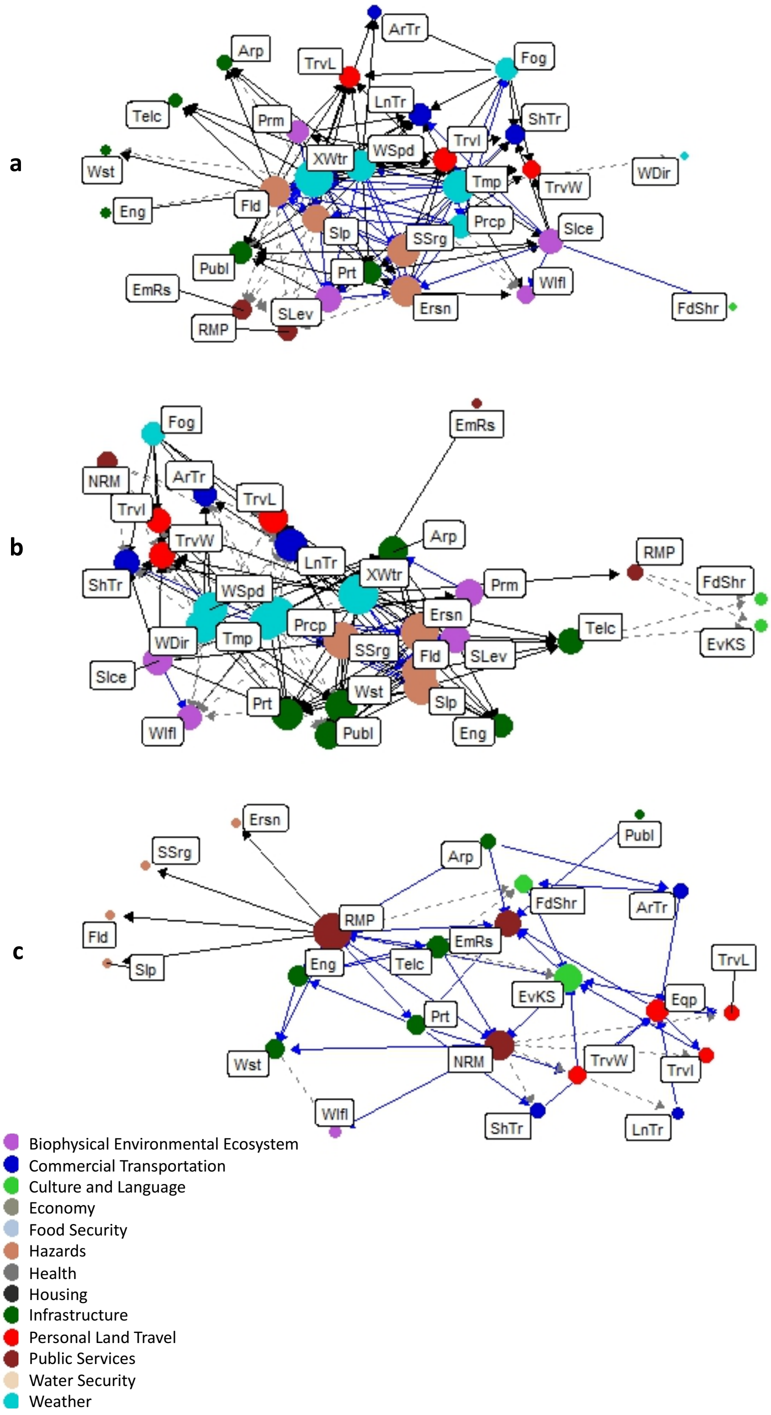

Supplementary Figure 6. Using data from the Canadian Flight Supplement, Canada Airport Charts and CADOR database, we calculated relative sensitivity of all Inuit Nunangat airports. The map above shows relative sensitivity (higher number and darker red is more sensitive) and the amount of traffic based on Statistics Canada CANSIM data (larger the dot the higher the volume).

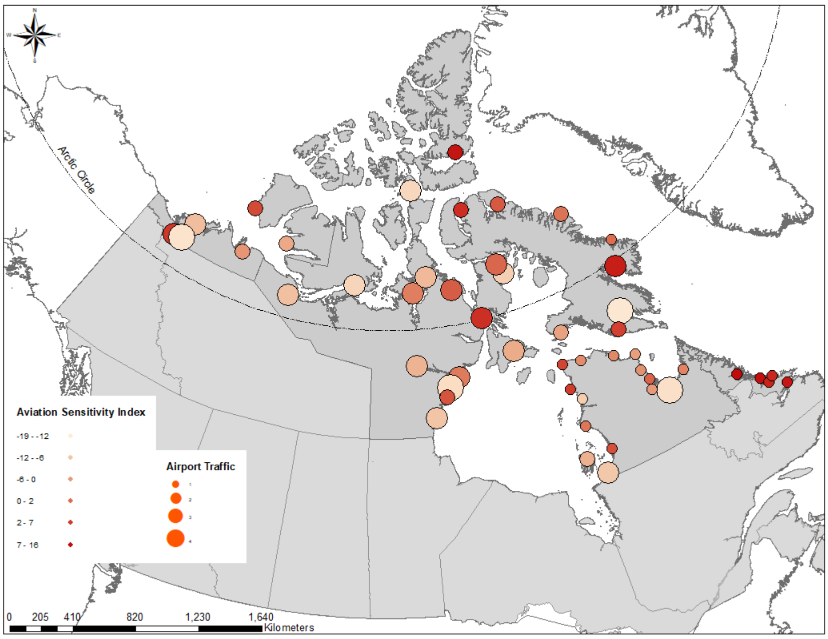

Supplementary Figure 7. The marine sensitivity index was based on variables that represented areas of past risks and the availability of risk reducing information and resources across the region.

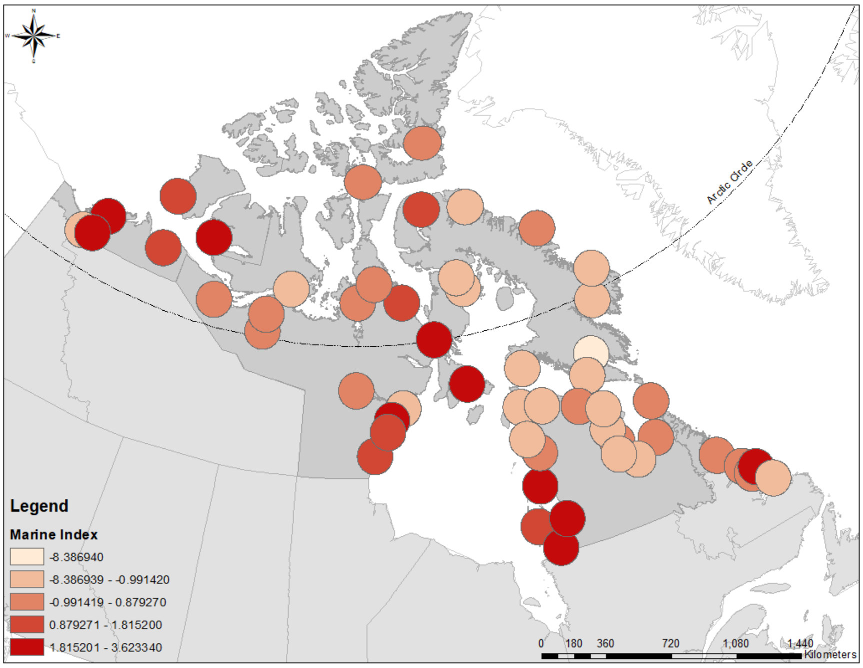

Supplementary Figure 8. Disaster sensitivity was assessed by weighting number of past disasters, RCAF search and rescue response times, and search and rescue incidence rates.

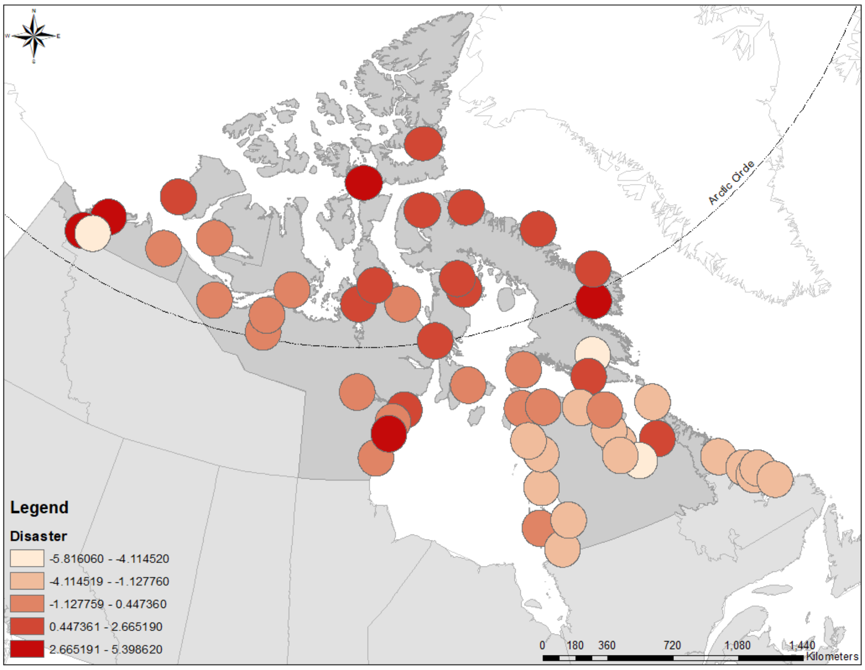

Supplementary Figure 9. Crosswind prevalence at all Inuit Nunangat airports was examined as a component of the Aviation Sensitivity Index. Mean wind directions for the past year were compared with runway heading. The map above shows prominent wind directions and the mean crosswind of each airport. Red, indicates higher degree of crosswind and therefore higher susceptibility. Wind direction was obtained through remotely sensed data (CCMP gridded data) and is likely impacted by local topography, particularly along Baffin Island.

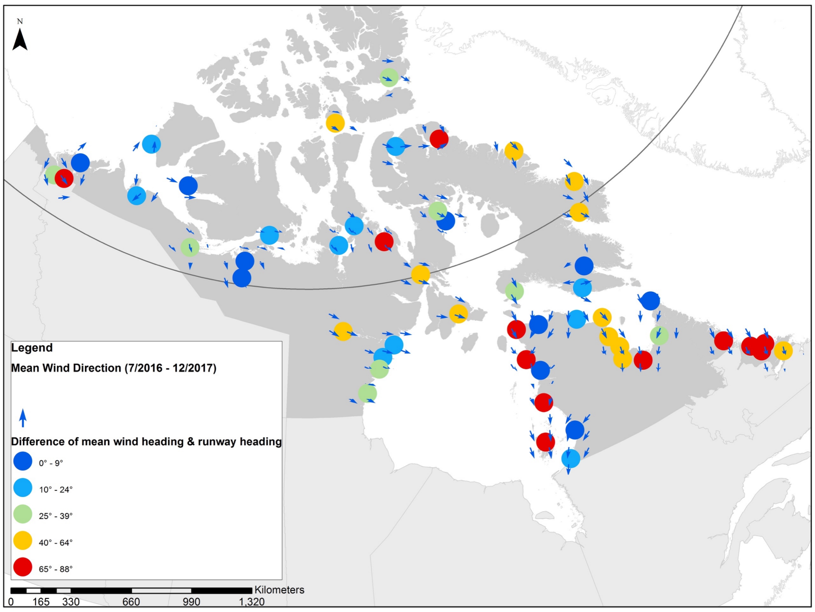

Supplementary Figure 10. Adaptive capacity was estimated using raw census data from Statistics Canada and from INAC's CWI. Estimated adaptive capacity produced spatial trends of lower adaptive capacity (Nunatsiavut) and higher adaptive capacity (Central Arctic).

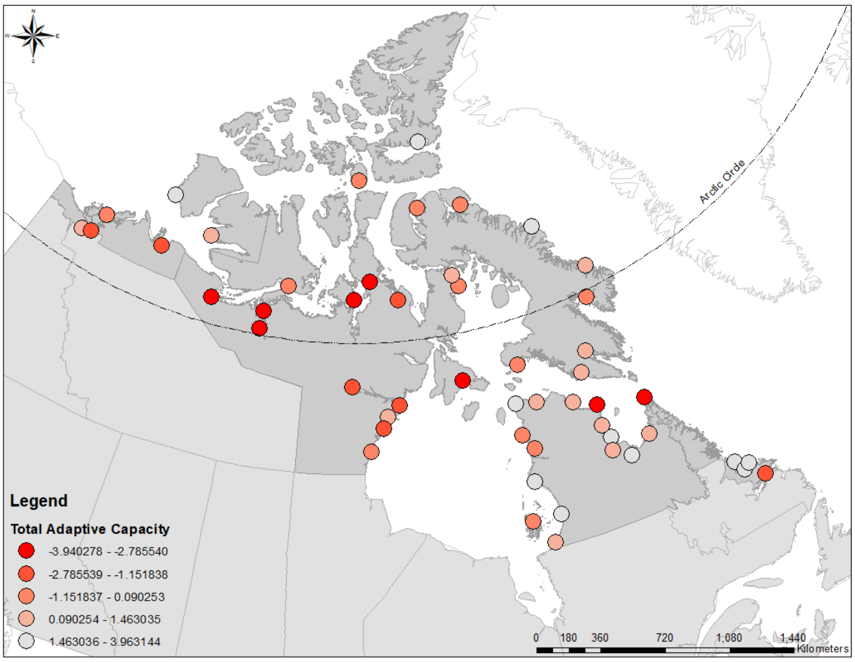

Supplementary Table 1. The four ensembles of simulations used in the study. The name of simulations is given as Institution-RCM-GCM-experiment. ARC-0.22 corresponds to Arctic CORDEX simulations with 0.22 degree of spatial resolution, ARC-0.44 corresponds to Arctic CORDEX simulations with 0.44 degree of spatial resolution, NA-0.22 corresponds to NA CORDEX simulations with 0.22 degree of spatial resolution and NA-0.44 corresponds to NA CORDEX simulations with 0.44 degree of spatial resolution. One asterisk (*) after a simulation indicates that this simulation had available only the variables daily minimum temperature, daily maximum temperature, daily mean temperature and daily total precipitation (and not daily solid precipitation or daily snow depths).

	**Arctic-Canada ensemble**<br><br>**(Institution-RCM-GCM-experiment)**	**Subarctic-Canada ensemble**<br><br>**(Institution-RCM-GCM-experiment)**
**RCP 4.5**	• CCCma-CanRCM4-CanESM2-ARC-0.22<br>• DMI-HIRHAM5-EC-EARTH-ARC-0.44<br>• OURANOS-CRCM5-CanESM2-NA-0.22<br>• SMHI-RCA4-CanESM2-ARC-0.44<br>• SMHI-RCA4-EC-EARTH-ARC-0.44<br>• SMHI-RCA4-MPI-ESM-LR-ARC-0.44<br>• SMHI-RCA4-NorESM1-M-ARC-0.44<br>• UQAM-CRCM5-CanESN2-NA-0.44<br>• UQAM-CRCM5-MPI-ESM-LR-NA-0.44	• CCCma-CanRCM4-CanESM2-NA-0.22<br>• DMI-HIRHAM5-EC-EARTH-NA-0.44<br>• OURANOS-CRCM5-CanESM2-NA-0.22<br>• SMHI-RCA4-CanESM2-NA-0.44<br>• SMHI-RCA4-EC-EARTH-NA-0.44<br>• UQAM-CRCM5-CanESN2-NA-0.44<br>• UQAM-CRCM5-MPI-ESM-LR-NA-0.44
**RCP 8.5**	• AWI-HIRHAM5-MPI-ESM-LR-ARC-0.44 *<br>• CCCma-CanRCM4-CanESM2-ARC-0.22<br>• DMI-HIRHAM5-EC-EARTH-ARC-0.44<br>• MGO-RRCM_MPI-ESM-LR-ARC-0.44<br>• OURANOS-CRCM5-CanESM2-NA-0.22<br>• SMHI-RCA4-CanESM2-ARC-0.44<br>• SMHI-RCA4-EC-EARTH-ARC-0.44<br>• SMHI-RCA4-MPI-ESM-LR-ARC-0.44<br>• SMHI-RCA4-NorESM1-M-ARC-0.44<br>• UQAM-CRCM5-CanESN2-NA-0.44<br>• UQAM-CRCM5-MPI-ESM-MR-NA-0.44	• CCCma-CanRCM4-CanESM2-NA-0.22<br>• DMI-HIRHAM5-EC-EARTH-NA-0.44<br>• IowaState-RegCM4-GFDL-ESM2M-NA-0.22*<br>• IowaState-RegCM4-HadGEM2-ES-NA-0.22*<br>• NCAR-RegCM4-MPI-M-MPI-ESM-LR-NA-0.22*<br>• OURANOS-CRCM5-CanESM2-NA-0.22<br>• SMHI-RCA4-CanESM2-NA-0.44<br>• SMHI-RCA4-EC-EARTH-NA-0.44<br>• UArizona-WRF-GFDL-ESM2M-NA-022*<br>• UQAM-CRCM5-CanESN2-NA-0.44<br>• UQAM-CRCM5-MPI-ESM-MR-NA-0.44

Supplementary Table 2. Climate indicators used in the study[1].

Acronym (Units)	Name	Definition
EDCSC (number of days from 1st August)	End date of continuous snow cover	First day after the SDCSC, when the snow depth < 2 cm for 14 consecutive days for a winter-centred year (from August to July)
FDD (degree days)	Freezing Degree Days	Cumulative sum of daily degrees of daily mean temperature below 0°C, over a winter-centred year (from July to June)
DJF mean Pr (mm/day)	Winter Mean precipitation (DJF)	December, January and February (DJF) average of daily precipitation
JJA mean Pr (mm/day)	Summer Mean precipitation (JJA)	June, July and August (JJA) average of daily precipitation
Annual mean Pr (mm/day)	Annual mean precipitation	Annual mean of daily precipitation
RX1day (mm/day)	Annual maximum 1-day precipitation	Annual maximum of daily precipitation
RX1day Snow (mm/day)	1-day maximum snowfall	Annual maximum of daily solid precipitation in a winter-centred year (from August to July)
RX5day (mm/day)	Annual maximum 5-day precipitation	Annual maximum of 5-day accumulated precipitation
SCD (days)	Snow cover duration	Annual number of days in a winter-centred year (from August to July) with snow depths ≥ 2 cm
SDCSC (number of days from 1st August)	Start date of continuous snow cover	First day of the 14 consecutive-day period with snow depths ≥ 2 cm for a winter-centered year (from August to July)
SDmax (m)	Maximum snow depth	Maximum snow depth during a winter-centred year (from August to July)
DJF mean T (°C)	Winter (DJF) mean temperature	December, January and February (DJF) average daily mean temperature (Tmean)
JJA mean T (°C)	Summer (JJA) mean temperature	June, July and August (JJA) average daily mean temperature (Tmean)
Annual mean T(°C)	Annual mean temperature	Annual average of daily mean temperature (Tmean)
TDD (degree days)	Thawing Degree Days	Cumulative sum of daily mean temperature above 0°C, over a calendar year (from January to December)
TNn (°C)	Annual coldest temperature	Annual minimum value of daily minimum temperature
TXx (°C)	Annual warmest temperature	Annual maximum value of daily maximum temperature

Supplementary Table 3. Soil types were categorized by risk associated with permafrost collapse and degradation potential.

Risk	Vulnerability Range	Soil Type
**-**	**0**	Water/Undivided/Glaciers/Colluvial Sand/Lag (Glacio)Marine/Colluvial Fines/Colluvial Rubble/Colluvial Blocks/Coarse grained (Glacio)Marine/Coarse grained (Glacio)Lacustrine
**Low**	**20**	Glaciofluvial Complex/Glaciofluvial Plain/Marine Mud
**Medium**	**50**	Till Veneer /Alluvial Deposits/ Till Blanket
**High**	**90-100**	Fine grained (Glacio)Marine/Alpine Complexes/Marine Sand/Lacustrine Sand/Organic Deposits

Supplementary Table 4. We used permafrost distribution from the Circum-Arctic map of permafrost and ground ice conditions as a component of the permafrost index.

Risk	Vulnerability Range	Permafrost type
-	0	Continuous Permafrost/No Permafrost
Low	30	Known Subsea Permafrost/Isolated Patches of Permafrost
High	80-100	Extensive Discontinuous Permafrost/Sporadic Discontinuous Permafrost

Supplementary Table 5. Elevation vulnerability was set according to the elevation of the Airports and Ports in the region. The lower the elevation higher the risk.

Altitude (meters)	Risk
0-10	High Risk
20-100…	Low Risk

Supplementary Table 6. Slope vulnerability was calculated from the Elevation Dataset considering degrees of inclination.

Slope (Degrees)	Risk
0	Low Risk
20	High Risk
80-100	Very High Risk

Supplementary Table 7. Water distance was calculated using hydrology maps (available at NRCan) including rivers, streams, lakes and ocean.

Distance km from buffer center to water body	Risk	Water body type
<1km	Very High	Ocean
>1-2km	High	
3-4km	Medium	
5km	Low risk	
<1km	Very High	Rivers
>1-2km	High	
3-4km	Medium	
5km	Low risk	
<1km	High	Lakes
>1-2km	Medium	
3-4km	Low	
5km	Low	

Supplementary Table 8. We conducted a systematic literature review of peer reviewed and grey literature from 1970-2017 related to vulnerability in the Canadian Arctic. Relationships and factors that influenced vulnerability were coded from the selected publications. The resulting 58 variables are listed below. These variables and their relationships were analyzed using multiplex network analysis.

Category	Variable	Abbr.	Category	Variable	Abbr.
**Weather**	extreme weather event	XWtr	**Biophysical Environment (Ecosystems)**	permafrost	Prm
	fog	Fog		eustatic sea level rise	SLev
	precipitation	Prcp		native ice flow animals	WlfI
	temperature	Tmp		native marine animals	WlFM
	wind direction variability	WDir		native plants	Plts
	wind speed	WSpd		native terrestrial animals	WlfT
**Hazards**	coastal erosion	Ersn		sea ice	SIce
	floods	Fld	**Health**	mental health	HthM
	slope failure	Slp		physical health	HthP
	storm surge	SSrg	**Commercial Transportation**	air transportation	ArTr
**Infrastructure**	public buildings and roads	Publ		overland transportation	LnTr
	airport infrastructure	Arp		shipping	ShTr
	energy availability	Eng	**Public Services**	health care	HthC
	ports	Prt		education quality and quantity	Edu
	telecommunications	Telc		emergency response	EmRs
	waste management	Wst		natural resource management	NRM
**Housing**	housing quality	HsQt		risk management and future planning	RMP
	housing quantity	HsQl	**Culture and Language**	arts and traditional equipment/clothing production	Trd
**Economy**	cost of living	Cost		environmental knowledge and skills	EvKS
	fishing (commercial)	FshC		food sharing and social networks	FdShr
	informal income	Inflc		Indigenous language strength	Lang
	nat. resource extraction	NREx	**Water Security**	water access	WtrAc
	relative poverty	Pov		water quality	WtrQl
	tourism	Trsm		water quantity	WtrQt
	wage income	WgIc	**Personal Land Travel**	access to equipment	Eqp
**Food Security**	country food quality	CnQt		travel on the ice	TrvI
	country food quantity	CnQl		travel on the land	TrvL
	food access	FdAc		travel on water	TrvW
	store-bought food quality	StrQl			
	store-bought food quantity	StrQt			

Supplementary Table 9. Variables related to exposure, sensitivity, and adaptive capacity of Northern transportation systems were analyzed using a multiplex network analysis. The most influential variables – those that had the most connections – are listed in the table. Freeman's degree centrality is the sum of ingoing and outgoing connections the variable had in the specified layer.

Exposure Variable	Centrality	Sensitivity Variable	Centrality	Adaptive Capacity Variable	Centrality
Extreme weather (XWtr)	24	Extreme weather (XWtr)	18	Risk mgmt. & future planning (RMP)	19
Temperature (Tmp)<br>Storm surge (SSrg)	18	Coastal erosion (Ersn)	17	Natural resource mgmt. (NRM)	12
Flooding (Fld)	17	Precipitation (Prcp)<br>Storm surge (SSrg)	16	Environmental knowledge and skills (EvKs)	10
Wind speed (WSpd)<br>Coastal erosion (Ersn)	16	Temperature (Tmp)<br>Slope Failure (Slp)	15	Emergency response capacity (EmRs)	9
Slope failure (Slp)	13	Wind Speed (WSpd)<br>Wind Direction (WDir)	13	Access to equipment (Eqp)<br>Telecommunications (Telc)	7

Supplementary Table 10. Based on results from the network analysis (NA), key informant interviews (I), and the document review (DR), we developed a list of characteristics / variables that are likely correlated with exposure, sensitivity, or adaptive capacity of transportation systems in the Canadian Arctic. The column Source denotes where recommendations came from. If the characteristic was described in a reviewed document, citations are provided.

VARIABLE	SOURCE
**EXPOSURE**	
Sea level rise	**DR, I, NA**
Surface wind speed and direction	**DR, I, NA**
Precipitation	**DR, I, NA**
Icing, freezing precipitation, and fog	**DR, I**
Permafrost risk	**DR, I**
Temperature	**DR, NA**
Snow/ snow cover	**DR, I**
Slope/topography	**DR, I, NA**
**SENSITIVITY**	
Number of disasters and infrastructure damaging events	**DR, I**
Quality of emergency management plan and uptake	**DR, I**
Speed by which emergency resources could reach community	**DR, I, NA**
Average annual number of search and rescues per capita	**DR, I**
Rating of health centre capacity and resources	**DR, I**
Community elevation and elevation of critical infrastructure	**I, NA**
Local tidal graph and seabed topography	**DR, NA**
Quantity of marine navigation resources in area	**DR, I**
Number of marked shipping hazards near corridors	**DR, I**
Amount of shipping traffic near communities	**DR, I**
Presence of a small-craft harbour / port	**DR, I**
Power interruptions/dependability	**DR, I**
Presence of available aviation weather forecasts	**DR, I**
Runway characteristics	**DR, I**
Rating of airport building disrepair	**DR, I**
Presence of portable generator for airport and de-icing equipment	**DR, I**
Presence of a gravel crusher in the community	**DR, I**
Aviation navigational aids and minimum conditions	**DR, I**
**ADAPTIVE CAPACITY**	
Number of commercial transportation options to the community	**DR, I**
Internet bandwidth and number of providers	**DR, I**
Food security	**DR, I**
Health of community	**DR, NA**
Socio-economic conditions of households and community	**DR, I, NA**
Strength of Inuit Traditional Knowledge	**DR, I, NA**
Newcomers in community / social cohesion / temporary population	**DR, I**
Quality of representation and governance	**DR, I, NA**
% with high school diploma, trade certificate, or high level of traditional knowledge	**DR, I, NA**
% age between 14 and 65	**DR, I, NA**

Supplementary Table 11. Using data from the Civil Aviation Daily Occurrence Reporting system, we classified 14,608 incidents that occurred between 2000 and 2016 using the search terms listed here. Classifications of a closure due to missing weather, power failure, or for other reasons provided insights into airport operations and sensitivity.

Missing Weather	Power Failure	Closed During Public Hours
did not provide weather	power problem	closed during published hours
provide weather	electricity outage	NOTAM closed during
did not provide aerodrome weather	power failure	CARS closed
no observations	unable to restore full power	due to staff
missing observations	power outage	were closed
missing weather	powering	NOT wx
provide aerodrome radio and weather		NOT weather
missed weather		NOT observations
wx OBS		
wx obs		

Supplementary Note 1. The Canadian Arctic is experiencing numerous, and intersecting, impacts to human wellbeing including climate change, persistent healthcare gaps and health inequities, infrastructure disrepair, housing shortages, and food insecurity[1-4]. Despite a strong body of research about these issues in the Canadian Arctic, there are communities and regions that have received less attention, such as communities in central Nunatsiavut, smaller communities in central Nunavut, and regional hubs[5]. Research into anticipated economic benefits and subsequent trade-offs have been slow to emerge, with research into effects of shipping through the Northwest Passage only emerging recently[6]. Furthermore, few studies have examined future trends, linked climate projections into analyses, or quantified vulnerability at a community or regional scale.

Recent studies have outlined pervasive adaptation and infrastructure gaps as well as emergency management vulnerabilities throughout the Canadian Arctic. Concrete adaptation actions have also been found to be lacking throughout the Canadian Arctic. In Nunavut, the challenge of integrating long-term planning into often short-term budgets has been a noted constraint, while the Northwest Territories lacks any foundational adaptation action strategy[2,3]

Supplementary Note 2. While climate hazards have been affecting transportation infrastructure operations, navigation routes like the Northwest Passage have experienced increased ice free periods, affecting economic activities such as fishing, tourism, resources exploration and cargo transportation[4-8]. These changes also have potential for increased risk of oil spills, pollution, and disturbance of wildlife and ecosystems, ultimately affecting communities subsistence and traditional ways of life[9]. These indirect impacts of transportation on human systems is beyond the scope of this study, though it is not unimportant.

In addition to commercial shipping and aviation, intracommunity transit and travel to cabins and hunting grounds is commonly done on small watercraft, all-terrain vehicles (ATVs), and snowmobiles[10-12]. Termed being ‘on the land’, the ability to safety and regularly access country food, visit culturally significant sites, and access cabins is critical to community health and wellbeing[13,14]. Travel on the land has changed substantially over the past 40 years with transitions from dog-teams and small wooden boats to snowmobiles and 20’ aluminum boats[15]. While modeling vulnerability related to ‘on the land travel’ is beyond the scope of this study, we do examine rates of search and rescue throughout the region, the majority of which are related to non-commercial transportation.

At the time of this article, the only ice road connecting communities within Inuit Nunangat was the Aklavik-Inukvik ice road. The new all-season road connecting Tuktoyaktuk to Inuvik

was finished in November 2017 and is the only permanent road connecting any communities in the region. Despite the lack of roads for intracommunity travel across Inuit Nunangat, movement of people and goods within each community is highly dependent on mainly gravel roads. The community-maintained roads link airports to the hamlet, allow for water delivery to homes, provide a way for supplies to be moved from sea lifts to warehouses and businesses, and are key infrastructure for local economies. In Nunavut alone, there were over 5600 registered road vehicles in 2017[16]. Roads in the region require frequent maintenance throughout the year due to high use and environmental characteristics. While we do not focus explicitly on roads in this report, findings related to permafrost change, precipitation, and sea level rise are highly relevant. Additional important transportation within the Canadian Arctic include sub-arctic rail service, particularly the provision of rail service to Churchill, and ferry service in Nunatsiavut. These play important roles for the surrounding communities and promote low-cost transportation of goods and people[17].

Supplementary Note 3. Transportation systems across the North are highly reliant on community infrastructure, including energy delivery, communications, storage facilities, and airport terminals[18-20]. Many of these infrastructure systems have reached or will soon reach their life cycle[21,22]. In Nunavut, for example, 11 of the 24 airport terminal buildings operated by the Nunavut Department of Economic Development and Transportation need substantial upgrades or immediate replacement[22].

It was beyond the scope of this study to independently assess most of the critical infrastructure systems. However, their importance to the human security of communities should not be overlooked. Further, safe and reliable functioning of shipping and aviation systems is dependent on a wide network of critical infrastructure. These include: satellite systems, power, bridges, dams and levees, water treatment and distribution, sewage distribution, fuel holding tanks, VHF and airband repeaters and radio towers, docks, and gas stations. The lack of redundancy, as compared to southern communities, makes infrastructure systems across the Arctic particularly susceptible to a failure.

Supplementary Note 4. Climate indices used in the study were computed at center *Eau Terre Environment* of the National Institute of Scientific Research (INRS) in collaboration with Ouranos Consortium on Regional Climatology and Adaptation to Climate Change. Models were constructed with an ensemble of regional climate models (RCMs) that used two Coordinated Regional Climate Downscaling Experiment (CORDEX) experiments[23,24]. The Arctic CORDEX, which covers northern Canada, and the North America CORDEX, which covers a large portion of the North America but only southern Canada. The Arctic CORDEX

simulations were used to produce climate projections over the High Arctic, while the North America CORDEX simulations were used for Nunatsiavut and Nunavik regions (Supplementary Table 1).

The RCM simulations include historical runs and projections based on two Representative Concentration Pathways (RCP)[25]:

- RCP 4.5 is a medium-low emission scenario that corresponds to a radiative forcing peaking at 4.5 Wm^{-2} (≈ 650 ppm CO_2 equiv.) by 2100.

- RCP 8.5 is a high emission scenario in which the increasing greenhouse gas emissions lead to a radiative forcing pathway of 8.5 Wm^{-2} (≈1370 ppm CO_2 equiv.) by 2100.

RCMs are driven by coupled climate model simulations from the fifth Coupled Model Intercomparison Project[26]. See Appendix I for list of simulations considered for both regions. Climate indices were computed from daily data on the original grid of each model[27]. Definition of indices are described in Supplementary Table 2.

The baseline period is 1971-2000 and climate change projections were computed for three future periods: 2011-2040, 2041-2070 and 2071-2100. The RCM projected changes were next interpolated on a regular global latitude-longitude grid with a spatial resolution of 0.25° using the nearest neighbor remapping method and the grid-point median, minimum and maximum values were used to represent the most likely changes and the interval of RCP-projected values. Using a regular global grid allowed for both CORDEX RCM ensembles to be joined in the same map and integrated coverage across Inuit Nunangat. More details on the computation of indices and the construction of RCM ensemble over Canada are provided in[1,27].

Supplementary Note 5. Surface Wind Observations: We obtained wind surface data from the Cross-Calibrated Multi-Platform (CCMP) gridded surface vector winds product that triangulates surface wind conditions using satellite, moored buoy, and model wind data[28,29]. The wind data included monthly mean wind heading and wind speed from 2016-2018 at a resolution of 25-km. Average wind speed and heading was calculated for the period.

Sea Level Rise Projections: Relative Sea Level Rise Projections were obtained from Natural Resources Canada (NRCan)[30]. Sea level data was available as point data for 22 locations across the Canadian North, therefore we used exponential kriging calculations to estimate

regional coverage values[31]. Projections were available for RCPs 4.5 and 8.5 from 2007-2100. For the purpose of this analysis we considered the 2007-2010 period as our baseline, and the following projections 2010-2040, 2040-2070 and 2070-2100 as the future scenarios (in agreement with the climate data of this study). Relative sea levels were observed to be increasing in the Beaufort Sea and Labrador Sea, while decreases were noted in James Bay and Baffin Island due to isostatic rebound.

Soils Data: Soil data was obtained from NRCan Surface Material spatial data[32-34]. Surface material was classified based on the assumption that more porous soils retain higher ice content and are of higher risk. Soil types were categorized by risk associated with permafrost collapse and degradation potential. (Supplementary Table 3).

Permafrost Data: Permafrost is affected by air temperature, snow cover, topography, buffering effects of surface vegetation, organic covers and local geology[35-37]. The permafrost dataset accounted for regional permafrost distribution from the Circum-Arctic map of permafrost and ground ice conditions[38]. Data provided general spatial extents of permafrost distribution throughout the Canadian Arctic. Permafrost distribution was ranked for integration into the indices calculations (Supplementary Table 4). Similar procedures have already been developed for Canada[35,36].

Elevation Data: Elevation dataset was obtained from NRCan. The CDEM data set has 20-m of resolution and it is available at
ftp://ftp.geogratis.gc.ca/pub/nrcan_rncan/elevation/cdem_mnec/.
A mosaic of tiles was created to assembly the entire region. The Elevation file was reclassified according to specific vulnerability of Airports and Ports, for instance, the lower the elevation the more vulnerable the infrastructure (Supplementary Table 5).

Slope Data: Slope data was calculated from the NRCan Elevation dataset using the Slope tool in ArcGIS 10.6. Slope vulnerability was categorized from Low to High considering degrees of slope inclination. We considered a high risk for landslides and slope failure starting at 18 degrees of inclination (Supplementary Table 6). A similar classification procedure has already been developed for Canada[39].

Water Distance Data: Water distance was calculated using hydrology maps (available at NRCan) including rivers, streams, lakes and ocean (Supplementary Table 7). The closer the community center to water features higher the probably of water related hazards. Distance was computed using the center of the 100-km buffer (community center) to the nearest water

body (1-5km) types: ocean, rivers or lakes. Calculations were performed using ArcGIS 10.6. This classification procedure was inspired in a similar ranking developed for landslide susceptibility in Canada[39].

Supplementary Note 6. To build the exposure equations we used a multivariate linear regression analysis (MLR). The MLR is a statistical analysis that identifies unknown levels of correlation of involved variables by testing a hypothesis of possible relation of cause and effect between them. If such a relation is found, a mathematical model can be tuned to describe it[40,41]. This technique establishes relations between the thematic maps and the variables listed as important for each model type. However, since not all ratios were linear (soil, slope and permafrost) specific distribution and classification of weight for its characteristics had to be performed (Supplementary Note 5).

Thus, in order to obtain equations applicable to Inuit Nunangat reality, different variables were tested singly and combinations for significance and weights were obtained. The weights comprising the final exposure index were established after a lengthy iterative process. This process was done manually (trial and error) until an ''optimum'' model matching expert knowledge, climate change, risk disasters and Arctic-Canadian literature was achieved. Over 200 combinations of weights between the variables were performed. That is, the weights were not chosen arbitrarily, and therefore, the ''optimum'' model achieved represents the best adhesion between the used variables and the reference database.

The logic used in the exposure equations was that each phenomenon (rain, snow, temperature DJF-JJA and sea level rise) has unique components and characteristics which can produce hazards. If a precipitation pattern (rain or snow) or temperature trend is known for triggering or increasing exposure, this characteristic (which was represented by climate model averages and extreme indices) received a higher weight when compared to others. This concept was retrieved and adapted from recent studies assessing climate change vulnerability through indices at large territorial scales[40-43].

For example, the rain model considered that Rx1-day variable have greater impacts in exposure than Rx5-day or Annual Precipitation. This parameter choice resulted in a rain exposure map that considered singular weights for certain rainfall characteristics which were coupled to physical features of the area such as wind, slope, soil and water distance (see rain example in Supplementary Figure 1). Physical features were combined at the exposure baseline period and kept static at future projections.

To calculate future scenarios, climate model results (future deltas) were added to the baseline. The delta addition indicated the amount of change from the baseline. Therefore, increasing values in the future climate means that the same rainfall extreme considered in the historical period might occur with greater frequency and/or magnitude in the future. This approach was done sequentially for snow, temperature DJF-JJA and sea level rise (considering each model specificities).

When testing variables weight during the calibration phase multiplicative values given to the same variable (for e.g. 3.0 or 8.0) incurred in equal spatial distribution patterns but with extrapolations. For this reason, we decided to maintain multiplicative values between 0.5-3.0. Thus, when overlapping normalized variables (climate and physical features) using map algebra, it was detected that the spatial distribution of these did not completely overlap in some regions, mainly due to the ocean areas surrounding the Canadian Arctic and distinct distribution patterns for each physical variable. Therefore, the indices raw values only consider the natural occurrence of overlapping areas between climate and physical variables (Supplementary Figure 1). When natural occurrence was not possible for sea level rise (for e.g.) we extrapolated pixels values to a mean inside of each community buffer (100-km). This mean was used to overlap inland and ocean values.

We believe that the exposure calculation results had a good agreement with what was described in the literature and at the narrative of policymakers and local's interviews.

Supplementary Note 7. We estimated rain exposure using two extreme rain indices, Rx1-day (maximum rain in 1 day) and Rx5-days (maximum rain in 5 days) and mean annual precipitation. We weighted the indicators based on associated probability of a flood or slope failure, with the 1-day rain being weighted the most and the annual precipitation being weighted the least[44-49].

Type I Model

$$rain_{exposure} = \left(\frac{3.0 * (Rx1\ day) + 1.5 * (Rx5\ day) + 0.5 * \left(pr\ Annual\ precipitation_{mm\ per\ day}\right) + soils + slope + water\ proximity + wind}{9.5}\right)$$

Type II Model

$$rain_{exposure} = \left(\frac{3.0 * (Rx1\ day) + 1.5 * (Rx5\ day) + 0.5 * \left(pr\ Annual\ precipitation_{mm\ per\ day}\right)}{5}\right)$$

Supplementary Note 8. Snow exposure was estimated using values for 1-day maximum snow (Rx1), maximum snow depth, and duration of snow cover. Extreme snow accumulation in one day was weighted more in the equation because it is often accompanied by low visibility which often closes airports, ports, and can increase risk of power failures, making difficult to travel around communities (blizzard episodes). Total snow depth, and the amount of days with snow cover were assumed to cause less disruptions to commercial transportation. It should be noted that while snow cover is beneficial for traditional travel and movement on snowmobiles, this study did not assess these activities and viewed snow cover as having a negative impact[48-52].

Type I Model

$$snow_{exposure} = \left(\frac{3.0 * (Rx1\ snow\ day) + 2.5 * (SDmax\ maximum\ snow\ depth_m) + 1.0 * (SCD\ snow\ cover\ duration_{days}) + slope + wind}{9.5}\right)$$

Type II Model

$$snow_{exposure} = \left(\frac{3.0 * (Rx1\ snow\ day) + 2.5 * (SDmax\ maximum\ snow\ depth_m) + 1.0 * (SCD\ snow\ cover\ duration_{days})}{6.5}\right)$$

Supplementary Note 9. For present sea level exposure and future scenarios (RCP 4.5 and RCP 8.5) we used tabulated mean projected values (in cm) which were available at NRCan reports[30,53-56]. Since data for these regions include positive and negative values, we decided to divided the exposure analysis into airport and marine sectors starting at the exposure analysis step (not necessary for the other models). In the airport exposure model, we rescaled mean sea level values between 0-1 to account for the increase of vulnerability since the decrease of sea level was considered as no impact. For the marine sector we rescaled the mean sea level rise values between -1 and 1 to account for the impacts of decreasing sea levels in many Inuit Nunangat regions. It is important to consider a proper equation to rescale positive and negative while forcing 0. The rescaled results were multiplied by -1 to allow the correct distribution of values.

$$rescale_{-1\ to\ 1} = \text{Con}\left(\text{raster} < 0,\ \frac{(\text{raster} + \text{max value})}{\text{max value}}\right), \text{Con}\left(\text{raster} > 0,\ \frac{(\text{raster- min value})}{\text{min value}},\ \text{raster}\right)$$

The Antarctic sea level rise component available at the NRCan projections was not included at this analysis, since we felt this would bias the model. Though we believe analysis

considering this component should be included or incorporated for more in depth characterization on sea level rise.

Type I Model

$$sea\ level_{exposure} = \left(\frac{3.0 * (current\ sea\ level\ ) + soils + permafrost + wind + water\ proximity}{7}\right)$$

Type II Model

$$sea\ level_{exposure} = \left(\frac{3.0 * (current\ sea\ level)}{3}\right)$$

Supplementary Note 10. Temperature exposure was calculated using winter (December, January, February or DJF) and summer (June, July, August or JJA) mean temperatures, annual mean temperatures, extreme high temperature index (TXx) and thawing degree days (TDD). Winter temperature, summer temperature, and extreme temperature were weighted more as they play a major role influencing permafrost thaw according to the literature[47,57,58].

Type I Model

$$Temperature_{Exposure} = \left(\frac{3.0 * (JJA\ or\ DJF) +\ 2.0 * (TXx) +\ 1.5 * (TDD)\ +\ 1.0 * (tasAnnual\ _{mean}) + soils + permafrost}{10.5}\right)$$

Type II Model

$$Temperature_{Exposure} = \left(\frac{3.0 * (JJA\ or\ DJF) +\ 2.0 * (TXx) +\ 1.5 * (TDD)\ +\ 1.0 * (tasAnnual\ _{mean})}{7.5}\right)$$

Supplementary Note 11. Adaptive capacity was estimated using data from Statistics Canada and the Indigenous and Northern Affairs Canada (INAC) Community Wellbeing Index (CWI). This combination of data sources allowed us to weight socioeconomic conditions, housing conditions, education attainment, traditional knowledge, and demographics.

Developed by INAC, the CWI uses Statistics Canada census data to score communities based on socioeconomic wellbeing, education attainment, labor force activities, income, and housing. Aggregated 2011. CWI scores were used in this study as the 2016 CWI had not yet been published by INAC. The CWIscore was missing for 3 communities, which were given the mean regional score. The average CWI score across the region was 61, while the standard

deviation was 11.7. Iqaluit had the highest CWI score, while Naujaat had the lowest reported CWI score.

To supplement the CWI, we used data from the 2016 Canadian census. We looked at education attainment by using percent of high school diplomas, percent of trades certificates, and percent of the community that spoke Inuktut. In combining western markers of education attainment and one element of Inuit Traditional Knowledge (language), we hoped to better reflect the diverse types of knowledge that build resilience. However, we acknowledge that an individual can have high levels of Traditional Knowledge without speaking Inuktut and someone can have lower levels of Traditional Knowledge and speak Inuktut. We also examined the percent of the community that was new immigrants as a new immigrant population likely has fewer land skills, limited knowledge of the Arctic, and no Inuit Traditional Knowledge. The percent of the population that was younger than 14 and older than 65 was also assessed. Knowing that language is only one of the many aspects of Inuit identity and culture, we strongly agree that future work needs to develop ways of measuring indicators for Inuit Traditional Knowledge.

The average community percent population that had graduated from high school was reported at 10%. The community with the highest graduation rate was Ulukhaktok (19%) and the lowest was Taloyoak (4%). The average community percent population that had a trade certificate was 4%. Postville had the highest percent of individuals with a trade certificate (14%) while five communities reported 0%. Communities with the highest percentage of recent immigrants were Quaqtaq, Iqaluit, Inuvik, and Coral Harbour. Statistics Canada defines recent immigrant as individuals who have immigrated to Canada between 2011 and 2016. Naujaat had the youngest population with the median age of 18 and 43.1% of the population under 14. Postville had the oldest population with an average age of 41.2 and over 17.1% of the population over 65. All data was normalized prior to calculating the Adaptation Capacity Index (Supplementary Figure 10).

Supplementary Note 12. Systematic literature reviews have been increasingly applied in the human dimensions of climate change scholarship and in the Arctic as a means of methodically documenting, characterising, and analyzing knowledge on a topic and synthesizing key trends[59-61]. The process involved applying search parameters to select peer reviewed and grey literature on risks, vulnerability, or adaptive capacity in the Canadian Arctic, selecting relevant documents, and extracting variables and vulnerability relationships discussed[62]. To insure the widest possible understanding of vulnerability this process was not

specific for transportation systems, but all-encompassing vulnerability in the Canadian Arctic[62]. We then focused on transportation through the development of an infrastructure & transportation sub-model[62].

We began the review in Web of Knowledge (WOK) using the following syntaxes during the 1970-2017 period: (a) climat* chang* AND Inuit* (n = 240 records); (b) climat* chang* AND vuln* Inuit* (n = 83 records); (c) climat* chang* AND adapt* Inuit* (n = 87 records); and finally (d) res* AND Inuit* (n = 39 records). We also searched grey literature, including institutional reports, consultant reports, book chapters and conference proceedings. For the grey literature, the following syntaxes were used: (a) Inuit climate change (n = 588 records); (b) Inuit vulnerability (n = 437); (c) Inuit adaptation (n = 274 records); and Inuit resilience (n = 10 records).

Search terms led us to select an initial 1,758 articles, books, book chapters and conference proceedings for review. These documents were screened for relevance leading to a final 155 documents for review. A document review led us to identify 137 variables that are broadly influential in vulnerability across Inuit Nunangat. Many variables were considered sub-sets of larger categories, and therefore merged with the resulting dataset (*n* = 58) (Supplementary Table 8). It should be noted that the variable selection was not specifically for transportation and infrastructure systems, but an all-encompassing lens of Inuit livelihoods and community systems.

Next, we coded relationships for selected variables. Coding of relationships was conducted using three 2-dimensional matrices. Notations indicated a positive, negative, or unknown relationship between variables. Based on framings of contextual vulnerability as well as resilience index literature, we assume that relationships of exposure (E), sensitivity (S), and adaptive capacity (AC) could overlap and are optimally represented by a multiplex network approach. We begin by categorizing the system into E, S, and AC components. Since a given variable can influence multiple components of vulnerability (i.e. socio-economic status may influence both adaptive capacity and sensitivity), each vulnerability layer includes all variable interactions. To represent multiple kinds of interactions among variables (i.e., E, S, and AC) we created a multiplex network model, where variables are represented as nodes and can have multiple relationships, called edges, to other variables (i.e., E, S, and AC in our case). Each category of edge (E, S, and AC) is represented as a layer in the multiplex network and all nodes are present in each layer (though it is possible for a node to have no relationships within a specific layer)[62].

The conceptualization of the system as a multiplex model was important in order to allow each variable to have possible relationships in the E, S, and AC dimensions. We then used these relationships to help select variables for use in this vulnerability index. We considered within-layer freeman's degree centrality which measures all the edges a node has within a given layer of the multiplex network. Our calculations used directed data, meaning a relationship from A to B and B to A counted as two relationships. We also dichotomized the data, meaning that only presence/absence of a relationship was considered, not the kind of affect (e.g., direct or indirect relationships). Although our freeman's degree centrality scores do not account for the magnitude or kind of effect, they indicate the degree of structural influence in the network, and we assumed that variables that were highly influential in the network should be considered in our vulnerability index (Supplementary Table 9).

Exposure results demonstrated that storm surge and extreme weather were highly influential in the vulnerability of transportation and infrastructure in the region. Biophysical sensitivity variables had more connections than did social sensitivity variables. Of social sensitivity variables, the highest number of connections were among overland travel – roads & rail (13), waste management (11), ports (11), travel on the land for harvesting (10), airport infrastructure (10), permafrost (9), public buildings and infrastructure (9), travel on the ice for harvesting (8), shipping (8), and telecommunications (8). Adaptive capacity variables that had the most connections largely included governance and planning variables. Telecommunications (7), energy availability (6), and food sharing (4) were also ranked high. Among the entire network, the most important adaptive capacity variables were socio-economic factors, health, and governance variables (Supplementary Figure 5).

Finally, characteristics of vulnerability and indicator suggestions that came out of the document reviews, key informant interviews, and network analysis were refined to a list of tangible and practical indicators (Supplementary Table 10). When merging the indicators-variables we listed the ones that were directly relevant to the development of an index for aviation, marine, and related infrastructure vulnerability. This final step in selecting variables for the index was not based on the merits of the characteristic – all characteristics in the table below are seen as important – but rather the final selection was based on the availability of representative data.

Supplementary Note 13. Qualitative data was used to select variables for indices and to assist with interpretation of vulnerability model outputs.

Key informant interviews (n = 37) and document reviews were conducted to further inform variable selection and model development. Government and public-sector key informants were selected based on their job duties and affiliation with subject areas relevant to transportation, infrastructure, and climate change in the Canadian Arctic. A list of officials was compiled using public directories, the research team's knowledge of the region, and by participant snowballing. While we worked to conduct interviews in all settlement regions of Inuit Nunangat, research permits were not granted in time for us to conduct interviews in Nunatsiavut. Documents from all regions were reviewed however, and federal and private sector key informants were interviewed that were able to provide insight into all areas of Inuit Nunangat.

Government officials from provincial/territorial (n = 15) and federal levels (n = 18) were interviewed. Interviews were also conducted with industry representatives and regional organizations (n = 4). While the majority of territorial and municipal interviews took place in Nunavut (n = 10), interviews were also conducted with officials in Yukon, Northwest Territories and Quebec (n = 6).

Discussions were conducted in person (32), by email (2), and by phone/skype (3). Interviews were audio recorded or detailed notes were taken depending on the key informants' preference. Interviews were guided by set topics. As all key informants were not themselves the focus of the research and were interviewed in their official government capacity, written consent was not obtained, as per McGill REB standards. Ethics and research approval were obtained from McGill REB, Nunavut Research Institute, and the Aurora Research Institute.

Supplementary Note 14. Key Informants (Ottawa, Kuujjuaq, Iqaluit, Yellowknife, Quebec City)

Goals:

- Understand how policy makers view current vulnerabilities of transportation, infrastructure, and social systems in the North
- Understand how/in what capacity policy makers see policies adapting to climate change for infrastructure and livelihood system in the North
- Highlight what infrastructure and social systems are currently the most vulnerable and in what way
- Highlight what systems are currently least impacted by climate change
- Outline how policy makers think livelihood systems will be impacted by climate change 50 or 100 years from now

- Understand what knowledge gaps exist for policy makers / what do they want to know, but have not been told or there is not data on?
- Gain better insight into how components of vulnerability interact

Supplementary Note 15.

1. What is your job, what are your duties?
2. How long have you been in this position? Any relevant past jobs or work?
3. Do you see climate change having an impact on [select system or area] in the North? If so, in what way?
4. Not thinking about what may happen in the future, are there current impacts that you or others are seeing? If so, in what way?
5. What variables or indicators do you feel best represent the current state of [social or infrastructure area] best?
6. What communities are worst off in this area and which are doing the best? What is causing that difference?
7. If we think of all areas of life in Northern communities – transportation, community infrastructure, food and health security, going on the land and traditional knowledge – what are you most concerned about in terms of current impacts by climate change? Why?
8. Are there things you are doing a part of your job to address climate change impacts on your area? Are there barriers to what you or your department can do? What are they?
9. Is there information that would better help you or your department plan for potential climate change impacts on transportation systems?

Notes and transcripts from interviews were broken into themes of exposure, sensitivity, and adaptive capacity. Within each theme, we extracted described relationships and potential variables or data sources. Data was then compared to results from the document review and the multiplex network analysis. The aim was to gather information about potential data sources, results from previous vulnerability assessments and regional risk assessments, and gather insights about what indicators may be representative of vulnerability across the region. If there was disagreement between key informants and documents, the researchers further investigated the system dynamics with particular attention to location. As the models needed to provide a generalization of the entire region, final decisions were made based on relevance to the largest number of communities.

Documents published by territorial and federal departments and agencies as well as regional private and Indigenous organizations were reviewed. Data from documents supplemented interviews and allowed for broadened spatial coverage. Documents were selected using

government and organization open databases and online libraries as well as by reference from key informants.

We selected and reviewed 18 reports. 8 reports were from federal departments, 5 focused on Nunavut, and 3 were from the Northwest Territories, and 1 from an Inuit organization. The reports ranges from 2009 to 2018, with 6 reports focusing generally on climate change and adaptation, 7 on transportation systems, aviation, or coastal systems, 2 on emergency management, and 2 on climate indices development.

Supplementary Note 16.

- Cox, R. S. (2015). Measuring Community Disaster Resilience: A Review of Current Theories and Practices with Recommendations. Ottawa, ON, Government of Canada,
- Government of Canada (2009). National Round Table on the Environment and Economy. True North: Adapting Infrastructure to Climate Change in Northern Canada. Ottawa, ON.
- Government of Canada (2013). Quadrennial Search and Rescue Review.
- Government of Northwest Territories (2014). Northwest Territories Hazard Identification Risk Assessment. Yellowknife, NT, GNWT.
- Government of Northwest Territories (2018). Climate Change Strategic Framework 2018 - 2030. Draft for Public Comment. Yellowknife, NT, GNWT.
- Government of Nunavut (2008). Ingirrasiliqta: Nunavut Transportation Strategy. E. D. Transportation. Iqaluit, Nunavut.
- Government of Nunavut (2011). Upagiaqtavut: Climate Change Impacts and Adaptation in Nunavut. Iqaluit, Nunavut.
- Healey, G. (2015). Exploring Health-Related Indicators of Climate Change in Nunavut. Iqaluit, Nunavut, Qaujigiartiit Health Research Centre.
- Inuit Tapiriit Kanatami (2016). Inuit Priorities for Canada’s Climate Strategy. A Canadian Inuit Vision for Our Common Future in Our Homelands. Ottawa, ON, ITK.
- Lemmen, D. S., et al. (2016). Canada’s Marine Coasts in a Changing Climate. Ottawa, ON, Government of Canada.
- Nunavut, G. o. (2014). Nunavut Airports 20 Year Infrustructure Needs Assessment 2014 - 2034. Iqaluit, Nunavut, Government of Nunavut.
- Office of the Auditor General (2017). Civil aviation infrustructure in the North - Transport Canada. Reports of the Auditor General of Canada to the Parliament of Canada. Ottawa, ON, Government of Canada.

- Office of the Auditor General (2017). Report of the Auditor General of Canada to the Yukon Legislative Assembly – 2017. Independent auditor's report: climate change in Yukon. Ottawa, ON, Government of Canada.
- Office of the Auditor General (2017). Report of the Auditor General of Canada to the Northwest Territories Legislative Assembly, 2017. climate change in the Northwest Territories: independent auditor's report. Ottawa, ON, Government of Canada.
- Office of the Auditor General of Canada (2018). Climate Change in Nunavut. Report to Northern Legislative Assemblies. Ottawa, ON, Government of Canada.
- Palko, K. G. and D. S. Lemmen (2016). Climate Risks & Adaptation Practices For the Canadian Transportation Sector. Ottawa, ON, Government of Canada.
- Public Safety Canada (2017). An Emergency Management Framework for Canada: Third Edition. M. R. f. E. Management, Government of Canada.
- Stantec, D. C. (2013). Climate Change Adaptation Plan for the GNWT Department of Transportation. Yellowknife, NT, Department of Transportation

Supplementary Note 17. The calibration phase assessed two models. Type II model emphasized climate and physical features over sensitivity and adaptive capacity. In this model climate exposure was calculated independently, and physical features were added to sensitivity (marine or airport). In Type II model climate and physical features were coupled to airport or marine sensitivity before projections were conducted. This placed roughly two-thirds of the weight for climate and physical features and one-third of the weight for marine or airport sensitivity. Since results from Type II model were not representative, only Type I model results are assessed in this article.

Supplementary Note 18. Interpretation of results was informed by qualitative data (key informant interviews, document review, and multiplex network modeling). In reports and interviews where regional hazards or vulnerabilities were discussed across a region, we were able to verify the model results of specific sensitivity indices. These comparisons were made by examining model outputs of current conditions, cross-comparisons of where high and low risk locations were, and drawing from the teams' extensive experience working across the region. In cases where there were discrepancies between qualitative data and results, the research team re-examined both data sets and determined what model variables were likely leading to the discrepancy. If there was a data quality issue (missing data for location), researchers obtained a better data source or developed a method to estimate values (see community wellbeing index in Supplementary Note 11).

Vulnerability calculation for the baseline includes the vulnerability formula where exposure is summed to sensitivity (marine or airport) and then subtracted from adaptive capacity. These operations are illustrated in two different equations with the following formulas:

Supplementary Note 19.

$$\text{Rain ACCVI}_{marine} = \left(\frac{(\text{Rain}_{\text{baseline or projection}} + \text{Sensitivity}_{\text{marine\&disasters}})}{2}\right) * \left(0.50 + \frac{(1 - \text{Adaptive Capacity})}{2}\right)$$

$$\text{Rain ACCVI}_{airport} = \left(\frac{(\text{Rain}_{\text{baseline or projection}} + \text{Sensitivity}_{\text{airport\&disasters}})}{2}\right) * \left(0.50 + \frac{(1 - \text{Adaptive Capacity})}{2}\right)$$

$$\text{Snow ACCVI}_{\text{marine}} = \left(\frac{(\text{Snow}_{\text{baseline or projection}} + \text{Sensitivity}_{\text{marine\&disasters}})}{2}\right) * \left(0.50 + \frac{(1 - \text{Adaptive Capacity})}{2}\right)$$

$$\text{Snow ACCVI}_{\text{airport}} = \left(\frac{(\text{Snow}_{\text{baseline or projection}} + \text{Sensitivity}_{\text{marine\&disasters}})}{2}\right) * \left(0.50 + \frac{(1 - \text{Adaptive Capacity})}{2}\right)$$

$$\text{Temperature ACCVI}_{marine} = \left(\frac{(\text{Temperature}_{\text{DJF or JJA baseline or projection}} + \text{Sensitivity}_{\text{marine\&disaster}})}{2}\right) * \left(0.50 + \frac{(1 - \text{Adaptive Capacity})}{2}\right)$$

$$\text{Temperature ACCVI}_{airport} = \left(\frac{(\text{Temperature}_{\text{DJF or JJA baseline or projection}} + \text{Sensitivity}_{\text{airport\&disaster}})}{2}\right) * \left(0.50 + \frac{(1 - \text{Adaptive Capacity})}{2}\right)$$

$$\text{Sea Level ACCVI}_{marine} = \left(\frac{(\text{Sea Level}_{\text{baseline or projection}} + \text{Sensitivity}_{\text{marine\&disasters}})}{2}\right) * \left(0.50 + \frac{(1 - \text{Adaptive Capacity})}{2}\right)$$

$$\text{Sea Level ACCVI}_{airport} = \left(\frac{(\text{Sea Level}_{\text{baseline or projection}} + \text{Sensitivity}_{\text{airport\&disasters}})}{2}\right) * \left(0.50 + \frac{(1 - \text{Adaptive Capacity})}{2}\right)$$

Supplementary Note 20.

$$\text{Rain ACCVI}_{\text{marine}} = (\text{Rain}_{\text{Baseline or projection}} + \text{MarineDisaster Sensitivity}) - \text{Adaptive Capacity}$$

$$\text{Rain ACCVI}_{\text{airport}} = (\text{Rain}_{\text{Baseline or projection}} + \text{AirportDisaster Sensitivity}) - \text{Adaptive Capacity}$$

$$\text{Snow ACCVI}_{\text{marine}} = (\text{Snow}_{\text{Baseline or projection}} + \text{MarineDisaster Sensitivity}) - \text{Adaptive Capacity}$$

$$\text{Snow ACCVI}_{\text{airport}} = (\text{Snow}_{\text{Baseline or projection}} + \text{AriportDisaster Sensitivity}) - \text{Adaptive Capacity}$$

$$\text{Temperature ACCVI}_{\text{marine}} = (\text{Temperature}_{\text{DJF or JJA Baseline or projection}} + \text{MarineDisaster Sensitivity}) - \text{Adaptive Capacity}$$

$$\text{Temperature ACCVI}_{\text{airport}} = (\text{Temperature}_{\text{DJF or JJA Baseline or projection}} + \text{AirportDisaster Sensitivity}) - \text{Adaptive Capacity}$$

$$\text{Sea Level ACCVI}_{\text{marine}} = (\text{Sea Level}_{\text{Baseline or projection}} + \text{MarineDisaster Sensitivity}) - \text{Adaptive Capacity}$$

$$\text{Sea Level ACCVI}_{\text{airport}} = (\text{Sea Level}_{\text{Baseline or projection}} + \text{AirportDisaster Sensitivity}) - \text{Adaptive Capacity}$$

Supplementary Note 21. Indicators related to missing weather observations and power failures were constructed using data from 2000 to 2016. Incidents and events were selected that were related to an aerodrome or airport in Inuit Nunangat. Of these 14,608 selected events, we conducted word searches of the narratives in order to classify the event as an occurrence of 'missing weather', 'power failure', or 'closed during public hours' (Supplementary Table 11). We also conducted searches for telecommunications failures, however this data was not included in the final aviation index.

We found that 21 airports had reported telecommunication failures between 2000 and 2016, with a mean outage time of 435 minutes per event. 34 airports reported at least one power failure during the period, with an average outage time of 344 minutes. The mean cumulative power outage time for the 16-year period was 1176 minutes or 19.6 hours. 36 airports reported at least one closure due to poor weather conditions, and 41 airports had posted at least one NOTAM due to missing weather observations. The mean time that airports did not have any available weather information was 482 minutes per event, with a mean total outage time during the 16-years of 78,517 minutes – an average of 54 days per airport.

There were noted cases of airports under-reporting closures, missing weather, and power failures. This was particularly noticeable in Nunatsiavut where there were few cases of aerodromes posting Notice to Airmen (NOTAM) for poor weather conditions as noted by examination of historic weather data and comparisons to rates of closures across the region. For airports that had not reported any issues or that were significantly lower than proximal airports of a similar size, the average regional score for airports with a similar volume of traffic was used for that airport.

Additional data for the aviation index was gathered from the Canadian Flight Supplement (CFS) and the Canada Airports Charts publications released on October 12, 2017. Variables included, runway length, width, and heading, and runway surface. Runway length is seen as a limiting factor across the region (the majority of airport runways are less than the Northern Air Transportation Association desired length of 5000')[18,21]. Short runways limit the size and

weight of aircrafts that can land, with subsequent impacts on travel and food costs in the community as well as ability of emergency resources to easily access the hamlet, airlift in fuel, or evacuate people quickly. Runway heading is important when compared to prominent wind direction. The difference between runway heading and wind direction – or crosswind – makes landing and taking off more challenging, risky, and if wind gusts are high enough will restrict access to the community. Finally, runway surfaces are an important factor in sensitivity of airports across the North. There are trade-offs to both asphalt and gravel options, with gravel being easier to maintain, costing less, and being easier to remove ice from, but severely limiting what aircrafts can land at the airport and increasing the operation costs of flying (due to high aircraft wear on the surface). Asphalt on the other hand requires high levels of training and resources to maintain, has high initial costs, and is more susceptible to permafrost changes[18,19]. For this study, we viewed gravel runways as being more sensitive due to the increasing amount of medium-size aircraft that are incapable of landing on gravel, the increased wear to aircraft[63]. Numerous informants expressed concern about increasing lack of jets that could land on gravel runways, given that Boeing 737 gravel packages are no longer being manufactured for newer aircraft[63].

The availability of terminal aerodrome forecast (TAF), hours the terminal is open, listed cautions, IFR approaches available, and IFR minimums were also obtained from the CFS. As described above, it is essential that pilots are able to obtain aviation forecasts for flight planning in the region, given the dependence on airports for medical evacuations, it is important that forecasts and observations are available all operational hours[19,21]. CFS cautions signify unusual approaches, dangerous surrounding terrain, or potential for hazards on the runway. The presence of a caution was seen as decreasing access to the community and increasing risk of flight to the airport. Finally, the presence of navigational aids and related minimums are important markers of how easy it is for planes to access the community, the impact that poor weather conditions have on aviation in the hamlet, and ability of medical evacuations to operate[18].

Runway length varied widely across the region. The shortest runway is in Grise Fiord at 1,675(ft.), while the longest was in Iqaluit at 8,605(ft.). There were 4 paved runways out of the 50 airports, these were Inuvik, Iqaluit, Kuujjuaq, and Rankin Inlet. Runway surfaces were ranked as a binary (gravel 0, paved 1) for the index. TAFs were available 24hours (automatically recorded) at 19 airports, while the average recording time at non-automatic TAF stations was 9 hours. 16 airports did not provide TAFs. A binary scale of the presence of TAF auto observations was developed for the index.

The majority of airports across the region had both NDB and RNAV approaches (*n* = 30), 6 airports only had RNAV approaches, 6 airports only had NDB approaches, 4 airports had RNAV, NDB, and VOR approaches, and 4 precision approaches. To rates the available navigational aids available, we categorized only having NDB approaches as a *1*, having NDB and RNAV approaches was a *2*, an NDB, VOR, and RNAV approach was a *3*, and any ILS approach was a *6*. The scale was chosen based on interviews and vertical and horizontal accuracy of the approaches.

Minimum descent altitude was converted to above ground level of airport surface, with the average (excluding precision approaches which use decision height) of 640 (ft.) AGL. The highest minimum descent altitude was in Grise Fiord at over 3000 (ft.) AGL.

CFS cautions were noted for 11 airports, the majority of cautions were due to surrounding terrain and difficulty of approach, although there were a few cautions due to potential for wildlife on the runway. A binary caution scale was developed for the index. Night flights were allowed at all airports except Nain, NL. A binary night flight scale was developed for the index.

Data on air traffic frequency was collected from Statistics Canada CANSIM database on air transportation. Air traffic movement data between January 2005 to January 2017 for all airports without air traffic control towers and airports with NAVCAN service stations were retrieved. Data was broken down into flight categories of (defense/government, private, and domestic commercial). There was available data for 16 communities.

There was an average of 163 government/DND flight plans per month across the region. The majority of DND and government flights were in Iqaluit (*n* = 47/month), Inuvik (*n* = 25/month), Kuujjuaq (*n* = 15/month), and Resolute (*n* = 14/month). There was an average of 6513 reported commercial flights in the region per month, with the most in Iqaluit (n = 651/month), Inuvik *(n* = 5 53/month), Rankin Inlet (*n* = 520/month), and Kuujjuaraapik (*n* = 479/month). Private flights accounted for 206 flights per month. Data on airport traffic was not used for the index as there was significant collinearity between airport traffic and other variables.

Finally, we examined wind direction prevalence and offsets to runway heading. Wind direction was obtained from the remote sensed Cross Calibrated Multi-Platform gridded surface observations[29]. Runway heading was obtained from the CFS. In the case of multiple runways (Kuujjuaq), the longest runway was selected. All runway headings were converted to

True North headings using declinations calculated by NRCan Declination Calculator (referenced of January 1, 2018 declinations). Wind offsets, or mean degrees of crosswinds, were calculated for each community (Supplementary Figure 9).

This resulted in a score from the highest sensitivity of 15.73 (Nain) to lowest sensitivity of -18.97 (Iqaluit) (Supplementary Figure 6). We observed a negative correlation between the amount of air traffic of an airport and the rated sensitivity. This correlation is likely at least a partial positive feedback loop. There was a large hot spot noted in Nunatsiavut, where by nearly all indicators has more sensitive aviation transportation systems than any other region in the Arctic. Airports across Nunavik were generally ranked as low or medium sensitivity, with the exception of Ivujivik and Akulivik.

Supplementary Note 22. Department of Fisheries and Oceans (DFO) provided data on locations of navigational aids (NAVTEX), electronic navigational chart coverage, reported anchoring grounds, and marine traffic were available across the region and applied. The presence of marine navigational aids and electronic navigational charts was discussed in numerous documents and by key informants as being an important factor in how safe an area is to travel through or off-load cargo in[18,19]. NAVTEX, which transmit information on weather and marine conditions, were seen as reducing susceptibility to incidents in the coverage areas. The amount of marine traffic in an area was seen as both a sensitivity, due to potential shoreline erosion, impacts from pollution or potential spills, and disruptions to local harvesters[53,64]. However, benefits of improved navigational knowledge of the area, potential for economic opportunities through tourism, and increased community access were also mentioned. Data provided by DFO was geospatial, allowing us to sample community values using a 75-km buffer.

Data on marine incidents that occurred between January 2004 and October 2017 were obtained from the Transportation Safety Board of Canada Marine Safety Information System. It was assumed that areas that had experienced previous groundings or incidents were more prone to future incidents and were more hazardous[19].

Using marine incident data, all incidents within 75km of an Inuit Nunangat community were linked to the closest community. Over the period, there were 41 reported incidents in the region. There were 7 incidents near Tuktoyaktuk and 4 near Iqaluit. 36 of the vessels were Canadian flagged ships, 2 were German, 1 was from Panama, 1 from Sweden, and one involved a barge and not flagged. 21 of the incidents were due to grounding or coming in contact with the ship hull. There was pollution reported from 4 of the incidents.

Data indicated the highest sensitivity was in Tuktoyaktuk, while the lowest was in Iqaluit (Supplementary Figure 7). Sensitivity in Nunavik and Baffin Island was generally lower than sensitivity in central and western Arctic. Influenced by an elevated number of shipping incidents in the Inuvialuit, hot spots were observed in the western Arctic. Coverage of navigation infrastructure along Baffin Island and through the Hudson Strait decreased sensitivity in the eastern Canadian Arctic.

Supplementary Note 23. Search and rescue rates were calculated based on data from Public Safety Canada's Knowledge Management System for years 2013 and 2014. More recent data was not available. Using ArcGIS spatial analysis tools, each event was joined with the closest proximal community. Incidence rates were calculated using Statistics Canada 2016 population data. The majority of search and rescue events were related to subsistence hunting and traditional travel on the land[65,66]. High search and rescue rates were seen as being an indicator of sensitivity for a variety of reasons. Research has demonstrated that communities with high search and rescue rates likely place high demands on emergency volunteers in the community and have high volunteer burnout rates[67]. There is also likely a connection between lower traditional knowledge levels and/or higher community hazards and elevated search and rescue rates.

Data on disasters near or in communities was also used as an indicator of sensitivity. Similar to tracking of marine incidents, it was assumed that communities that had previous disasters have more hazards. While it is possible that these communities have learned and adapted to reduce risks, the tracking of past disasters is often used to assess future risks[19,68,69]. Public Safety maintains a database of qualifying disasters across Canada. To qualify an event has to meet one of more of the following criteria: 1) 10 or more people killed; 2) 100 or more people affected/injured/infected/evacuated or homeless; 3) an appeal for national/international assistance; 4) historical significance; or 5) significant damage/interruption of normal processes such as that the community affected cannot recover on its own. Between 1900 and 2018, there were 6 qualifying disasters reported in Inuit Nunangat. Three events were due to flooding, one due to storm surge, one due to an avalanche, and one due to a major aviation disaster.

When the Royal Canadian Air Force (RCAF) does respond to the region, it is usually from its southern air force bases. Canadian Air Force SAR bases are located in out of Greenwood, NS, Gander, NL, Trenton, ON, Winnipeg, MB, and Comox, BC. In terms of Arctic SAR resources, CC-130 Hercules and CH-149 Cormorants are usually used. Additionally, the CH-

146 Griffon and CC-138 Twin Otter are used depending on availability, location of the incident, and performance demands. Canadian Coast Guard are usually only used when icebreakers are North during the summer, however this is changing with prolonged shipping seasons.

Data for aircraft performance was obtained from the RCAF website and reports[70]. Variables used to calculate response time were our best estimates given publicly accessible data. These figures should not be taken as true representations of performance, but simple the best estimates we were able to obtain.

We briefly examine the response time of the expected new SAR fixed wing aircraft C-295, though more research is needed. The methods and variables used present a 'best case' scenario for the one-way response time. Further, we assumed that aircraft were available and serviceable from the closest CFB that is home to SAR fixed or rotor wing. For example, if an incident were to occur in Kuujjuaq, the CH-149 from CFB Gander offers the lowest response time – this is the time registered in the maps below. CFB Trenton was not the closest response base for fixed or rotor wing for any Arctic community, however based on RCAF response area jurisdictions, CFB Trenton is the primary response base for incidents across much of the Central Arctic, potentially making actual responses slower than our estimates. It is important to consider that the estimated response times do not include and time searching, but simply arriving in the vicinity of a disaster or search.

Based on interpolated outputs, each community was given a value for the amount of time we estimated it would take for a CC-130 and RCAF helicopter to reach the location.

Finally, the number of physicians was seen as being important to immediate response to an incident and the preparedness for major events[71]. Although many communities have allied health professionals that operated with an expanded scope of practice, data was not available to independently track number of critical care nurses. The number of physicians per capita was calculated based on Statistics Canada 2016 data. The mode number of physicians residing in an Inuit Nunangat community is 0. There were only three communities where one or more physicians reported residing in the 2016 census, Iqaluit (n = 25), Inuvik (n = 15), and Kuujjuuaq (n = 10). Physicians are regularly in other communities on medium and short-term rotational basis and were not counted in the census.

Data suggested the highest sensitivity to disasters was in Resolute, while the lowest was in Kuujjuaq (Supplementary Figure 8). Sensitivity was generally highest on Baffin Island and

the high Arctic. Due to the RCAF response time component of the Disaster Sensitivity Index, more northern communities were generally considered more sensitive. Hot-spots were observed across Baffin Island, near Whale Cove, and near Tuktoyaktuk.

Supplementary Discussion. Linking human and physical systems across a region as large as the Canadian Arctic has not been frequently done and has never been attempted to our knowledge anywhere in the circumpolar North. There are notable challenges and limitations of joining social and physical data and projecting future change. For example, the adaptive capacity index developed in this study only accounts for a small portion of potentially influential factors, reflecting data availability. Governance and institutional processes, for example, are highly influential in the ability of a community to have the economic resources necessary for transportation adaptation in the Canadian Arctic. Though limited, the adaptive capacity index we developed does correlate to some extent with past work identifying adaptation projects across Nunavut[72]. Noticeable differences between our model and observed adaptation projects are likely attributable to the influence that a single individual that champions adaptation can make in a community[3,72]. In many of the communities across the region, adaptation initiatives that have happened have been the result of a concentrated group of public officials and private citizens that focus resources on initiatives.

There are numerous limitations to this study. Limitations revolve around three areas, qualitative validation, data availability, model assumptions. Although we worked to triangulate model development using key informant interviews, a systematic literature review, and document review, we undoubtedly were not able to and account for the multitude of observations and perspective that would be found in each community throughout the region. Results and model assumptions should be further validated through discussions and input from communities across Inuit Nunangat. Numerous datasets that may have improved model estimates was not available for this study. This includes, but is not limited to ice projection data, data on non-commercial marine incidents, and data on community road quality.

Additionally, though not assessed, there are numerous types of transportation, particularly travel by ATV or snowmobile that are of critical importance in the region. Further, the spatial scale of available restricted our ability of looking at intracommunity or neighborhood adaptive capacity. It is important to account for the high levels of inequality within many Inuit Nunangat communities which would substantially elevate some household's susceptibility in communities like Iqaluit. Indeed, in Inuit Nunangat the median income for Inuit is $17,778, while the median income for non-Indigenous is $77,683[73].

The modeling techniques we applied have numerous assumptions and limitations. Firstly, social change is not accounted for in the models. Additionally, in human systems, there are thresholds by which risk of a disaster may be amplified, further risk of an incident may exponentially increase with incremental social or physical environmental shifts[74,75]. This

vulnerability modeling does not capture these realities. Many of the limitations of this study are not unique to this modeling, but broader challenges of research that quantifies and projects vulnerability. More research is needed to further test the model, refine linked physical and social modeling and assess other important livelihood components, such as health and food security. It would also be beneficial if future research worked to link projections of social changes or adaptation pathways into models.

Supplementary References

1 Diaconescu, E. P., Mailhot, A., Brown, R. & Chaumont, D. Evaluation of CORDEX-Arctic daily precipitation and temperature-based climate indices over Canadian Arctic land areas. *Climate Dynamics*, doi:10.1007/s00382-017-3736-4 (2017).

2 Report of the Auditor General of Canada to the Northwest Territories Legislative Assembly. (Government of Canada, Ottawa, ON, 2017).

3 Ford, J. D., Labbé, J., Flynn, M. & Araos, M. Readiness for climate change adaptation in the Arctic: a case study from Nunavut, Canada. *Climatic Change* **145**, 85-100, doi:10.1007/s10584-017-2071-4 (2017).

4 AMSA. Arctic Marine Shipping Assessment 2009 Report. 194 (Arctic Council 2009).

5 Kajan, E. Arctic Tourism and Sustainable Adaptation: Community Perspectives to Vulnerability and Climate Change. *Scandinavian Journal of Hospitality and Tourism* **14**, 60-79, doi:10.1080/15022250.2014.886097 (2014).

6 Jensen, Ø. Arctic shipping guidelines: towards a legal regime for navigation safety and environmental protection? *Polar Record* **44**, doi:10.1017/s0032247407007127 (2008).

7 Smith, L. C. & Stephenson, S. R. New Trans-Arctic shipping routes navigable by midcentury. *Proc Natl Acad Sci U S A* **110**, E1191-1195, doi:10.1073/pnas.1214212110 (2013).

8 Østreng, W. *et al. Shipping in Arctic Waters: A Comparison of the Northeast, Northwest and Trans Polar Passages*. Vol. 1 381 (Praxis Publishing - Springer, 2013).

9 Ng, A. K. Y., Andrews, J., Babb, D., Lin, Y. & Becker, A. Implications of climate change for shipping: Ports and supply chains. *Wiley Interdisciplinary Reviews: Climate Change* **9**, doi:10.1002/wcc.508 (2018).

10 Laidler, G. J., Elee, P., Ikummaq, T., Joamie, E. & Aporta, C. in *SIKU: Knowing Our Ice* 45-80 (Springer, 2010).

11 Aporta, C. The Trail as Home: Inuit and Their Pan-Arctic Network of Routes. *Human Ecology* **37**, 131-146 (2009).

12 Aporta, C. Routes, trails and tracks: Trail breaking among the Inuit of Igloolik. *Inuit Studies* **28**, 9-38 (2004).

13 Cunsolo Willox, A. *et al.* Climate change and mental health: an exploratory case study from Rigolet, Nunatsiavut, Canada. *Climatic Change* **121**, 255-270, doi:10.1007/s10584-013-0875-4 (2013).

14 Parlee, B. & Furgal, C. Well-being and environmental change in the arctic: a synthesis of selected research from Canada's International Polar Year program. *Climatic Change* **115**, 13-34, doi:10.1007/s10584-012-0588-0 (2012).

15 Wenzel, G. Inuit and Modern Hunter-Gatherer Subsistence. *Inuit Studies* **37**, 181-200 (2013).

16 Canada, S. CANSIM. (Government of Canada, 2017).

17 Lemmen, D. S. Canada’s Marine Coasts in a Changing Climate. (Government of Canada, Ottawa, ON, 2016).

18 Stantec, D. C. Climate Change Adaptation Plan for the GNWT Department of Transportation. (Department of Transportation, Yellowknife, NT, 2013).

19 GNWT. Northwest Territories Hazard Identification Risk Assessment. (Government of Northwest Territories, Yellowknife, NT, 2014).

20 Canada, G. o. in *True North: Adapting Infrustructure to Climate Change in Northern Canada* (Government of Canada, 2009).

21 General, O. o. t. A. Civil aviation infrastructure in the North - Transport Canada, in Reports to the Auditor General of Canada to the Parliament of Canada. (Governmnet of Canada, Ottawa, ON, 2017).

22 Nunavut, G. o. Nunavut Airports 20 Year Infrustructure Needs Assessment 2014 - 2034. (Government of Nunavut, 2014).

23 Giorgi, F., C. Jones & Asrar, G. R. Addressing climate information needs at the regional level: the CORDEX framework. 175 (World Meteorological Organization (WMO), 2009).
24 Gutowski Jr, W. J. WCRP coordinated regional downscaling experiment (CORDEX): a diagnostic MIP for CMIP6. 4087 (2016).
25 van Vuuren, D. P. *et al.* The representative concentration pathways: an overview. *Climatic Change* **109**, 5-31, doi:10.1007/s10584-011-0148-z (2011).
26 Taylor, K. E., Stouffer, R. J. & Meehl, G. A. An Overview of CMIP5 and the Experiment Design. *Bulletin of the American Meteorological Society* **93**, 485-498, doi:10.1175/bams-d-11-00094.1 (2012).
27 Diaconescu, E. P., Gachon, P. & Laprise, R. On the Remapping Procedure of Daily Precipitation Statistics and Indices Used in Regional Climate Model Evaluation. *Journal of Hydrometeorology* **16**, 2301-2310, doi:10.1175/jhm-d-15-0025.1 (2015).
28 Atlas, R. *et al.* A Cross-calibrated, Multiplatform Ocean Surface Wind Velocity Product for Meteorological and Oceanographic Applications. *Bulletin of the American Meteorological Society* **92**, 157-174, doi:10.1175/2010bams2946.1 (2011).
29 Wentz, F. & al., e. *Cross-Calibrated Multi-Platform (CCMP) 6-hourly ocean vector wind analysis product on 0.25 deg grid, Version 2.0*, 2015).
30 James, T. S. *et al.* Relative Sea-level Projections in Canada and the Adjacent Mainland United States. 72 (Geological Survey of Canada, 2014).
31 ArcGIS Descktop (Environmental Systems Research Institute, Redlands, CA, 2014).
32 Fulton, R. J. Quaternary Geology of Canada and Greenland: Supplement. (International Specialized Book Service Incorporated, 1989).
33 Fulton, R. J. Vol. 84 (ed Geological Survey of Canada) (1984).
34 NRCan. in *Atlas of Canada* (Government of Canada, 2017).
35 Smith, S. L. & Burgess, M. M. (ed Geological Survey of Canada) 24 (Government of Canada, Ottawa,ON, 2004).
36 Jorgenson, M. T. *et al.* Resilience and vulnerability of permafrost to climate changeThis article is one of a selection of papers from The Dynamics of Change in Alaska's Boreal Forests: Resilience and Vulnerability in Response to Climate Warming. *Canadian Journal of Forest Research* **40**, 1219-1236, doi:10.1139/x10-060 (2010).
37 Forbes, D. L., Bell, T., James, T. S. & Simon, K. M. Reconnaissance assessment of landscape hazards and potential impacts of future climate change in Arviat, southern Nunavut. 183-192 (Canada-Nunavut Geoscience Office, 2013).
38 Brown, J., Ferrians, J., O.J., Heginbottom, J. A. & Melnikov, E. S. (ed National Snow and Ice Data Center/World Data Center for Glaciology) (Boulder, CO, 2001).
39 Bobrowsky, P. T. & Dominguez, M. J. (Geological Survey of Canada, Ottawa, ON, 2012).
40 Peduzzi, P., Dao, H., Herold, C. & Mouton, F. Assessing global exposure and vulnerability towards natural hazards: the Disaster Risk Index. *Natural Hazards and Earth System Sciences* **9**, 1149-1159 (2009).
41 Debortoli, N. S., Camarinha, P. I. M., Marengo, J. A. & Rodrigues, R. R. An index of Brazil's vulnerability to expected increases in natural flash flooding and landslide disasters in the context of climate change. *Natural Hazards* **86**, 557-582, doi:10.1007/s11069-016-2705-2 (2017).
42 Camarinha, P. I. M., Debortoli, N. S. & Hirota, M. Índice de vulnerabilidade aos desastres naturais relacionados às secas no contexto da mudança do clima: estudo completo. Report No. ISBN: 978-85-7738-312-2, 125 (World Wildlife Fund, Brazil (WWF), Brasília-DF, Brazil, 2017).
43 Menezes, J. A. *et al.* Mapping human vulnerability to climate change in the Brazilian Amazon: The construction of a municipal vulnerability index. *PLoS One* **13**, e0190808, doi:10.1371/journal.pone.0190808 (2018).
44 Sillmann, J., Kharin, V. V., Zhang, X., Zwiers, F. W. & Bronaugh, D. Climate extremes indices in the CMIP5 multimodel ensemble: Part 1. Model evaluation in the

present climate. *Journal of Geophysical Research: Atmospheres* **118**, 1716-1733, doi:10.1002/jgrd.50203 (2013).
45 Sillmann, J., Kharin, V. V., Zwiers, F. W., Zhang, X. & Bronaugh, D. Climate extremes indices in the CMIP5 multimodel ensemble: Part 2. Future climate projections. *Journal of Geophysical Research: Atmospheres* **118**, 2473-2493, doi:10.1002/jgrd.50188 (2013).
46 Bintanja, R. & Andry, O. Towards a rain-dominated Arctic. *Nature Climate Change* **7**, 263-267, doi:10.1038/nclimate3240 (2017).
47 Plummer, D. A. *et al.* Climate and Climate Change over North America as Simulated by the Canadian RCM. *Journal of Climate* **19**, 3112-3132 (2006).
48 Hanesiak, J. M. & Wang, X. L. Adverse-Weather Trends in the Canadian Arctic. *Journal of Climate* **18**, 3140-3156 (2005).
49 Hanesiak, J. *et al.* Storm studies in the Arctic (STAR). *Bulletin of the American Meteorological Society* **91**, 47 (2010).
50 Barnes, E. A. Revisiting the evidence linking Arctic amplification to extreme weather in midlatitudes. *Geophysical Research Letters* **40**, 4734-4739, doi:10.1002/grl.50880 (2013).
51 Stendel, M., Christensen, J. H. & Petersen, D. in *High-Arctic Ecosystem Dynamics in a Changing Climate Advances in Ecological Research* 13-43 (2008).
52 Davis, R. E., Lowit, M. B., Knappenberger, P. C. & Legates, D. R. A climatology of snowfall-temperature relationships in Canada. *Journal of Geophysical Research: Atmospheres* **104**, 11985-11994, doi:10.1029/1999jd900104 (1999).
53 Stewart, E. J. *et al.* Local-level responses to sea ice change and cruise tourism in Arctic Canada's Northwest Passage. *Polar Geography* **36**, 142-162, doi:10.1080/1088937X.2012.705352 (2013).
54 Beveridge, L. *A method for assessing coastal vulnerabilities to climate change within an arctic community: the example of Tuktoyaktuk, Northwester Territories* MSc Marine Management thesis, Dalhousie University, (2013).
55 Smith, L. C. & Stephenson, S. R. New Trans-Arctic shipping routes navigable by midcentury. *Proceedings of the National Academy of Sciences* **110**, E1191 (2013).
56 Bjorkman, A. D., Elmendorf, S. C., Beamish, A. L., Vellend, M. & Henry, G. H. Contrasting effects of warming and increased snowfall on Arctic tundra plant phenology over the past two decades. *Glob Chang Biol* **21**, 4651-4661, doi:10.1111/gcb.13051 (2015).
57 Simpkins, G. Snapshot: Extreme Arctic heat. *Nature Climate Change* **7**, 95-95, doi:10.1038/nclimate3213 (2017).
58 Comiso, J. C. & Hall, D. K. Climate trends in the Arctic as observed from space. *Wiley Interdisciplinary Reviews: Climate Change* **5**, 389-409 (2014).
59 Ford, J. D. *et al.* Adapting to the Effects of Climate Change on Inuit Health. *American Journal of Public Health*, e1-e9 (2014).
60 Ford, J. D., Berrang-Ford, L. & Paterson, J. A systematic review of observed climate change adaptation in developed nations. *Climatic Change* **106**, 327-336, doi:10.1007/s10584-011-0045-5 (2011).
61 Mahood, Q., Van Eerd, D. & Irvin, E. Searching for grey literature for systematic reviews: challenges and benefits. *Res Synth Methods* **5**, 221-234, doi:10.1002/jrsm.1106 (2014).
62 Debortoli, N. S., Sayles, J. S., Clark, D. G. & Ford, J. D. A systems network approach for climate change vulnerability assessment. *Environmental Research Letters* **13**, doi:10.1088/1748-9326/aae24a (2018).
63 Pincus, R. Large-Scale Disaster Response in the Arctic: Are We Ready? , 234 (2015).
64 Dawson, J., Johnston, M. & Stewart, E. Governance of Arctic expedition cruise ships in a time of rapid environmental and economic change. *Ocean & Coastal Management* **89**, 88-99 (2014).
65 Clark, D. G. *et al.* The role of environmental factors in search and rescue incidents in Nunavut, Canada. *Public Health*, 44-49 (2016).

66 Young, S., Tabish, T., Pollock, N. & Young, T. Backcountry Travel Emergencies in Arctic Canada: A Pilot Study in Public Health Surveillance. *International Journal of Environmental Research and Public Health* **13**, 276 (2016).
67 Clark, D. G., Ford, J. D., Pearce, T. & Berrang-Ford, L. Vulnerability to injuries associated with land-use activities in Nunavut, Canada. *Social science & medicine* **169**, 18-26 (2016).
68 Cutter, S. L. The landscape of disaster resilience indicators in the USA. *Natural Hazards* **80**, 741-758, doi:10.1007/s11069-015-1993-2 (2015).
69 Cutter, S. L., Ash, K. D. & Emrich, C. T. The geographies of community disaster resilience. *Global Environmental Change* **29**, 65-77, doi:10.1016/j.gloenvcha.2014.08.005 (2014).
70 Services, C. R. Evaluation of the DND/CAF Contribution to the National Search and Rescue Program. (Governement of Canada, Ottawa, ON, 2015).
71 Cox, R. S. Measuring Community Disaster Resilience: A Review of Current Theories and Practices with Recomendations. (Government of Canada, Ottawa, ON, 2015).
72 Labbé, J., Ford, J. D., Araos, M. & Flynn, M. The government-led climate change adaptation landscape in Nunavut, Canada. *Environmental Reviews* **25**, 12-25, doi:10.1139/er-2016-0032 (2017).
73 Kanatami, I. T. Social Determinants Of Inuit Health In Canada. 44 (Inuit Tapiriit Kanatami, Ottawa, ON, 2014).
74 Field, C. B. *et al. Climate Change 2014: Impacts, Adaptation, and Vulnerability. Part A: Global and Sectoral Aspects. Contribution of Working Group II to the Fifth Assessment Report of the Intergovernmental Panel on Climate Change.* (Cambridge University Press, 2014).
75 Barros, V. R. *et al. Climate Change 2014: Impacts, Adaptation, and Vulnerability. Part B: Regional Aspects. Contribution of Working Group II to the Fifth Assessment Report of the Intergovernmental Panel on Climate Change*. Vol. II (Cambridge University Press, 2014).

}
