# Supplementary material for: An integrative climate change vulnerability index for Arctic aviation and marine transportation
Source: Nat Commun. 2019 Jun 13;10:2596. doi: 10.1038/s41467-019-10347-1 (PMC6565733; doi:10.1038/s41467-019-10347-1)
Supplement: Supplementary file 2 — Description of Additional Supplementary Files [file 41467_2019_10347_MOESM2_ESM.pdf]

Description of Additional Supplementary Information

File Name: Supplementary Data 1
Description: Exposure values for the exposure indices.

File Name: Supplementary Data 2
Description: Values for the Adaptive Capacity, Airport Sensitivity, Marine Sensitivity, and Disaster Sensitivity indices.

File Name: Supplementary Data 3
Description: Vulnerability values for the vulnerability indices.

File Name: Supplementary Data 4
Description: Increment values for the increment/anomalies indices.
